# Are depressive disorders caused by psychosocial stressors at work? A systematic review with metaanalysis

**DOI:** 10.1007/s10654-021-00725-9

**Published:** 2021-02-12

**Authors:** Sigurd Mikkelsen, David Coggon, Johan Hviid Andersen, Patricia Casey, Esben Meulengracht Flachs, Henrik Albert Kolstad, Ole Mors, Jens Peter Bonde

**Affiliations:** 1 Department of Occupational and Environmental Medicine, Bispebjerg and Frederiksberg Hospital, University of Copenhagen, Copenhagen, Denmark; 2 MRC Lifecourse Epidemiology Unit, University of Southampton, Southampton, UK; 3 Department of Occupational Medicine, Danish Ramazzini Centre, University Research Clinic, Herning, Denmark; 4 Department of Psychiatry, Mater Misericordiae University Hospital, Dublin, Ireland; 5 Department of Occupational Medicine, Danish Ramazzini Centre, Aarhus University Hospital, Aarhus, Denmark; 6 Department of Psychosis, Aarhus University Hospital – Psychiatry, Aarhus, Denmark

**Keywords:** Depressive disorders, Psychosocial stressors at work, Causality, Common method bias, Diagnostic misclassification

## Abstract

In the last decade, many studies have examined associations between poor psychosocial work environment and depression. We aimed to assess the evidence for a *causal* association between psychosocial factors at work and depressive disorders. We conducted a systematic literature search from 1980 to March 2019. For all exposures other than night and shift work and long working hours, we limited our selection of studies to those with a longitudinal design. We extracted available risk estimates for each of 19 psychosocial exposures, from which we calculated summary risk estimates with 95% confidence intervals (PROSPERO, identifier CRD42019130266). 54 studies were included, addressing 19 exposures and 11 different measures of depression. Only data on depressive episodes were sufficient for evaluation. Heterogeneity of exposure definitions and ascertainment, outcome measures, risk parameterization and effect contrasts limited the validity of meta-analyses. Summary risk estimates were above unity for all but one exposure, and below 1.60 for all but another. Outcome measures were liable to high rates of false positives, control of relevant confounding was mostly inadequate, and common method bias was likely in a large proportion of studies. The combination of resulting biases is likely to have inflated observed effect estimates. When statistical uncertainties and the potential for bias and confounding are taken into account, it is not possible to conclude with confidence that any of the psychosocial exposures at work included in this review is either likely or unlikely to cause depressive episodes or recurrent depressive disorders.

**Supplementary information:**

The online version of this article (10.1007/s10654-021-00725-9) contains supplementary material, which is available to authorized users.

## Introduction

Depressive disorders are common and have important consequences for affected individuals and society more widely [1]. The 12-month prevalence of one or more depressive episodes in the general population in Western Europe and North America has been estimated as approximately 6% [2–6]. Because of healthy worker selection, the prevalence is generally lower in working populations [7–12]. Estimates of *lifetime* prevalence between 12 and 27% have been reported from epidemiological surveys using structured clinical interviews for diagnosis [3, 13–16], but these may have been spuriously low because of underreporting due to poor recall [17–19].

Psychosocial aspects of work have been linked with various adverse health effects, including depressive symptoms and depression [20]. Most studies have focused on possible harmful effects of high demands, low control, job strain, low support, and effort-reward-imbalance.

A serious methodological problem in many investigations is that measures of exposure and outcome have not been independent, which may have inflated risk estimates [21]. Strategies to avoid this bias have been to use work-unit or job averages of perceived exposures, or to employ expert ratings or objective measures of psychosocial factors at work.

In the last decade there have been six systematic reviews of cohort studies on the associations between “clinical” depression and psychosocial factors at work [20, 22–26]. They included from six to 16 studies and related different measures of depression to one or more exposures. One concluded that methodological limitations precluded causal inference for demonstrated associations [20], but the others made no explicit statements on causality.

Against this background, the Danish Labour Market Insurance and Occupational Diseases Committee commissioned a more comprehensive review to evaluate the strength of evidence that long-lasting stress at work causes the development of depressive disorders. The ultimate purpose was to determine the case for designating depression as a compensable occupational disease. In this report, we present the findings of the review, which examined the evidence for a causal association of depressive episodes with a range of psychosocial factors at work, taking into account the validity of diagnostic methods, adjustment for potential confounders and potential bias from non-independent assessments of exposure and outcome.

## Methods

The review was conducted and is reported in accordance with the preferred reporting method for systematic reviews (PRISMA) [27]. The study protocol was registered at PROSPERO (https://www.crd.york.ac.uk/PROSPERO, identifier CRD42019130266).

### Literature search

We made a systematic search for original peer-reviewed full text papers in English that provided quantitative risk estimates for measures of depression in relation to psychosocial aspects of work. We searched PubMed, PsycNET and Web of Science, from 1980 to March 2019 (Supplementary material, Appendix 1).

Exposures were specified by search terms covering a wide range of psychosocial factors at work: 1) from Karasek’s job strain model[28]: demands, control, decision authority, skill discretion, support, job strain and iso-strain; 2) from Siegrist’s effort-reward-imbalance model[29]: effort, reward, effort-reward-imbalance and overcommitment; 3) procedural and relational injustice[30]; and 4) the following single factors: job insecurity, organizational restructuring, working hours, work load, work with deadlines, shift work, role conflict, conflicts with colleagues, conflicts with superiors, violence and threats, bullying (mobbing) and harassment, emotional strain, caregiving, social capital and meaningful work.

Outcomes were defined by search terms combining depressive disorders and depression with work-related stress, work, occupation, job and employment.

We required a specified study design defined by search terms cohort, prospective, longitudinal, intervention, case-crossover, case–control, cross-sectional, case-only, survey or intervention studies. Studies on depression in relation to post-traumatic stress disorder and pregnancy were excluded.

The systematic electronic search was supplemented by searching reference lists in retrieved papers and reviews. Reports in the “grey” literature were not included.

### Selection of studies

We required that the outcome was a dichotomous measure of depression that could be validated against a depressive episode diagnosed by a semi-structured interview. We excluded studies with mixed diagnoses, subthreshold diagnoses and non-specific diagnoses; studies of depressive episodes attributable to bipolar disorder; use of psychoactive substances or organic disorders; studies of unselected working populations with a point or 12-month prevalence > 10% of depression (see Supplementary material, Appendix 2, Table A2.1), because such a high prevalence of depressive episodes is unlikely in an unselected working population, and suggests serious diagnostic misclassification; and studies of sickness absence and disability-pensioning for depression because these outcomes have a dual set of risk factors, one related to social legislation and culture, and the other to the occurrence of depression. We accepted studies based on hospital diagnoses of affective disorders as a combined group because most such diagnoses are likely to indicate depressive episodes [2, 5, 14, 31]. Inclusion criteria for exposure were defined by exposure search terms. We excluded studies that used job title, occupation, profession or work in specified industries as indicators of workplace stress, with no other measure of exposure.

For cross-sectional and case–control studies we required that the impact of common method bias (i.e. bias because the assessment of exposure and outcome was not independent) was considered to be low. We accepted self-reports of night and shift work and working hours as fulfilling this criterion.

We excluded studies without a reference group and studies that did not provide a relative or absolute risk estimate or data that enabled the computation of such risk estimates. Studies identified from the literature search were screened for eligibility by reading titles and abstracts and those that passed this test progressed to reading of the full text. All titles and abstracts were read independently by SM and JPB. Disagreements on exclusions were resolved by consensus. Full text reading was divided between SM and JPB. Studies were only excluded without discussion if they fulfilled specific objective exclusion criteria (e.g. no case definition, no risk estimates, baseline case prevalence > 10%). This was a deviation from the protocol which specified independent full text reading of all studies by two investigators. The deviation was adopted because we judged that there would be minimal gain in precision from two independent assessments when the criteria for exclusion were specific and objective.

Details of the selection of studies are presented in Supplementary material, Appendix 2. We required at least three studies for causal assessment.

### Data extraction

We extracted information on type of exposure and whether it was assessed by self-report, an average measure for the work-unit, a job-exposure-matrix or a register.

Measures of depression were classified according to the diagnostic methods used to define a case: semi-structured interviews, fully structured interviews, self-administered questionnaire instruments, questionnaire self-reported doctor’s diagnosis, and register information on antidepressant treatment or hospital discharge diagnoses.

We extracted information on control for effects of twelve established potential confounders: age[3, 15, 32, 33]; sex [1, 3, 15, 19, 33, 34]; previous depression [35–37]; current depression at baseline in cohort studies; subthreshold depressive symptoms [33, 37]; genetic disposition as revealed by a family history of depression [38, 39]; predisposing personality traits [34, 40, 41]; marital status or living alone [3, 15, 19]; stressful life events [34, 42]; childhood adversities/distress[33, 37, 41, 43, 44]; low socioeconomic status (SES), assessed from education, income or job position [3, 15, 45, 46]; and somatic disorders/poor self-rated health [32, 47–49].

Control for depressive symptoms was only considered sufficient if based on a continuous score because even low level depressive symptoms at baseline are likely to confound the relation between psychosocial exposures at baseline and incident depressive episodes [12, 26, 50] (see Supplementary material, Appendix 3).

Control for current or previous depressive episodes was considered sufficient if based on structured interviews or questionnaire instruments, and insufficient if based on self-reported doctor’s diagnoses, antidepressant treatment or hospital discharge diagnoses because fewer than half of people with depressive episodes in the general population access the formal health care system for their depressive symptoms [51–54] (see Supplementary material, Appendix 4).

Control for socioeconomic status (SES) was deemed sufficient if a study took into account more than three SES categories defined by one or more of education, occupational grade and income.

We extracted estimates of relative risks, odds ratios and hazard ratios for measures of depression related to the highest exposure contrast, and within that, from the analytical model that was most fully adjusted. We also extracted several other descriptive statistics (see Supplementary material, Appendix 3,Table 3).

### Meta-analyses

Meta-analysis of risk estimates was performed by application of random effects modelling separately to each set of exposure-specific estimates, with weighting by the inverse variance of each estimate [55]. Summary estimates of relative risk were calculated, regardless of tests for heterogeneity since all studies were qualitatively heterogeneous. As the frequency of the outcome was low (< 10%), odds ratios and hazard ratios were treated as proxies for relative risk. Forest plots were used to illustrate risk estimates and their summaries. Funnel plots were used to assess evidence for publication bias. All analyses and plots were done in R v.3.6.0 (2019–04-26).

### Validity of measures of depression

To interpret the risk estimates that were reported from studies included in the review, it was important to understand the validity of the measures of depression that they employed, in terms of the estimated false positive and false negative rates when they were applied in samples selected from the general population. This was explored in separate analyses, using depressive disorders diagnosed by semi-structured diagnostic interviews as the standard. The methods of these supplementary analyses are presented in Supplementary material, Appendix 4. Suitable data were available only for ‘Depressive episode’ and ‘Recurrent depressive disorder’ as defined by the ICD-10 Classification of Mental and Behavioural Disorders codes F32 and F33 [56, 57], ‘Major depressive episode’ and ‘Major depressive disorder’ as defined by the Diagnostic and Statistical Manual of Mental Disorders IV (DSM-IV) codes 296.2 and 296.3 [58, 59] and corresponding diagnoses in previous revisions of these classification systems. For the purpose of the present study, diagnostic differences between the two classification systems were considered unimportant [60]. In what follows, we use the nomenclature of the ICD-10 system.

### Assessment of study quality

Completeness of reporting was assessed by SM and JPB with respect to study design, sampling procedures, inclusions and exclusions, participation rates, exposure assessment, outcome ascertainment and statistical analysis. Each item was scored 1 if satisfactory, and otherwise 0. The sum of item scores (range 0 to 7) was calculated to indicate the overall completeness of reporting as an indirect measure of study quality. The scoring scheme was a slightly modified version of that proposed by Bonzini et al. 2007 [61]. Each of us scored approximately half of the studies after initial calibration based on five studies.

## Results

### Literature search and selection of studies

The database literature search yielded 4206 papers for title/abstract screening and 138 papers for full text reading. After scrutiny of full text, we excluded 86 papers, leaving 52 papers for inclusion in the review. By scrutinizing literature references in these studies, we identified four additional papers that fulfilled the inclusion criteria [26, 62–64].

There were only 1–2 papers on organizational change/downsizing, social capital, managerial quality, iso-strain, meaning of work and role conflict. Five of these papers were not considered any further in this review because they did not include other relevant exposures [65–69]. Among the remaining 51 papers, three each included results from two independent sub-studies in the same report [70–72], and we treated these as separate studies. Altogether, therefore. we present results derived from 54 studies reported in 51 papers [7, 11, 12, 26, 46, 50, 62–64, 70, 72–112]. A flow chart giving further details of the literature search is shown in Supplementary material, Appendix 2.

### Description of the selected literature

Of the 54 studies, 47 were cohort investigations, five were cross-sectional, and two had a nested case–control design. They provided usable data (i.e. risk estimates for measures of depression from at least three studies) on 19 psychosocial exposures at work. Table 1 summarises the methods by which exposures and outcomes were assessed, and the extent of control for important potential confounders.

**Table 1:** Distribution of methods of exposure assessment, of diagnostic methods used to measure depression, and of potential confounders controlled for by exclusion or adjustment in the analyses. Number of studies (N)

Exposure assessment	N
Self-reported	44
Work-unit average	5
Job-exposure-matrix	2
Register information	2
Work-unit and self-report	1

Diagnostic methods^a^	N
*Semi-structured interview*	
SCAN	3
*Fully structured interview*	
CIDI versions, MINI	12
*Questionnaires*	
Established instruments: CES-D, HADS-D, MDI-algorithm, MHI-5, PHQ-9, Zung SDS,	9
Doctor’s diagnosis, self-report	7
*Register based outcomes*	
Antidepressant treatment	16
Doctor’s diagnosis, hospital	4
*Mixed methods*	
SCAN and MDI-algorithm	3

Potential confounders controlled for^b^	N
Previous depression^c^	4
Depression at baseline^c,e^	29
Depressive symptoms at baseline^d,e^	11
Family history of depression	5
Neuroticism/personality	6
Recent life events	10
Living alone/marital status	39
Childhood adversities	4
Socio-economic status^f^	32
Somatic illness/self-rated poor health	19

Number of potential confounders controlled for (excluding age and sex)	N
None	9
One	7
Two	6
Three	8
Four	9
Five	10
Six	2
Seven	3
Eight, nine or ten	0

^a^For references, see Supplementary material, Appendix 3, textbox to forest plots

^b^Age and sex was controlled for in all but one study

^c^Sufficient control by interview or questionnaire instrument cases at baseline

^d^Sufficient control by continuous score

^e^Only relevant for cohort studies

^f^Sufficient if based on more than 3 categories

Exposures were assessed by self-report in 44 studies, by participant-independent methods (work-unit, job-exposure-matrix or register information) in nine studies, and by both methods in one study.

Outcome measures of depression were based on structured diagnostic interviews, questionnaires and antidepressant treatment in 12, 16 and 16 studies, respectively. Only three studies were based exclusively on a semi-structured interview (Schedules for Clinical Assessment in Neuropsychiatry (SCAN)[113]). Three studies on bullying assessed depression partly by SCAN and partly by a questionnaire instrument (Major Depression Inventory (MDI)-algorithm [114]) because they merged data from two different studies.

All but one study controlled for age and sex. Other factors commonly taken into account were current depression at baseline (29 studies) and living alone/marital status (39 studies). The factors most seldom controlled for were previous depression (4 studies), childhood adversities (4 studies), family history of depression (5 studies), neuroticism/personality (6 studies) and recent life events (10 studies). No studies controlled for all the three measures of baseline depression status (current depression, previous depression and depressive symptoms). Ten studies controlled for current depression and for depressive symptoms, and four controlled for current and previous depression [88, 95, 98, 101].

Twenty-four studies controlled for four or more of the potential confounders, and fifteen for five or more.

Further descriptive characteristics, overall and by specific study, are presented in Supplementary material, Appendix 3.

### Risk estimates related to a single exposure assessment

**Fig. 1:**
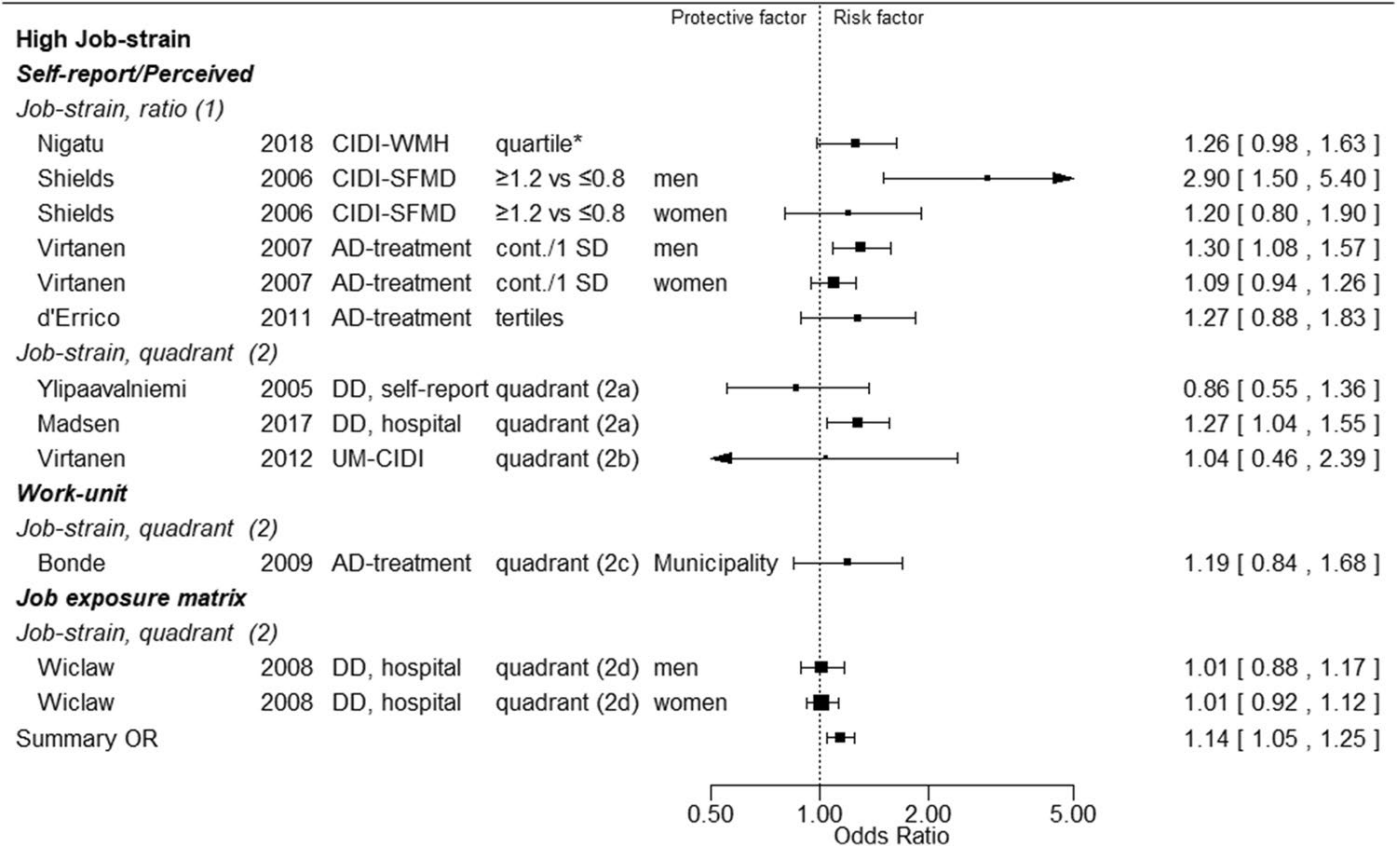
Forest plot of job-strain. * Column 1*: Method of exposure ascertainment and first author. *Column 2*: publication year. *Column 3*: Diagnostic method. *Column 4*: Exposure contrast. *Column 5*: substudy/submaterial. *Column 6 and 7*: risk estimates and their 95% confidence intervals. **Abbreviations**: *Column 1*: job strain ratio: demands scale score (higher score for higher demands) divided by control scale score (higher score for higher control); job strain quadrant: combination of median (or other percentile) split of demands scale score and control scale score: low strain=low demands and high control, passive=low demands and low control, active=high demands and high control, high stress=high demands and low control. *Column 3*: AD: antidepressant; CIDI: Composite International Diagnostic Instrument; CIDI-SFMD: Short Form version of CIDI, Major Depression module; CIDI-WMH: WHO Mental Health version of CIDI; DD, hospital: doctor’s diagnosis from hospital discharge letter; DD, self-report: doctors diagnosis, self-reported; UM-CIDI: University of Michigan version of CIDI. *Column 4*: 2a: high strain quadrant vs the other three quadrants combined, median split; 2b: high strain quadrant vs the low strain quadrant, median split; 2c: high strain quadrant vs the other three quadrants combined, upper quartile split; 2d: high strain quadrant vs the other three quadrants combined, upper tertile split; cont./1SD: continuous, by unit of one scale standard deviation; quartile*: upper quartile versus other three quartiles combined; tertiles: upper tertile versus lowest tertile

**Fig. 2:**
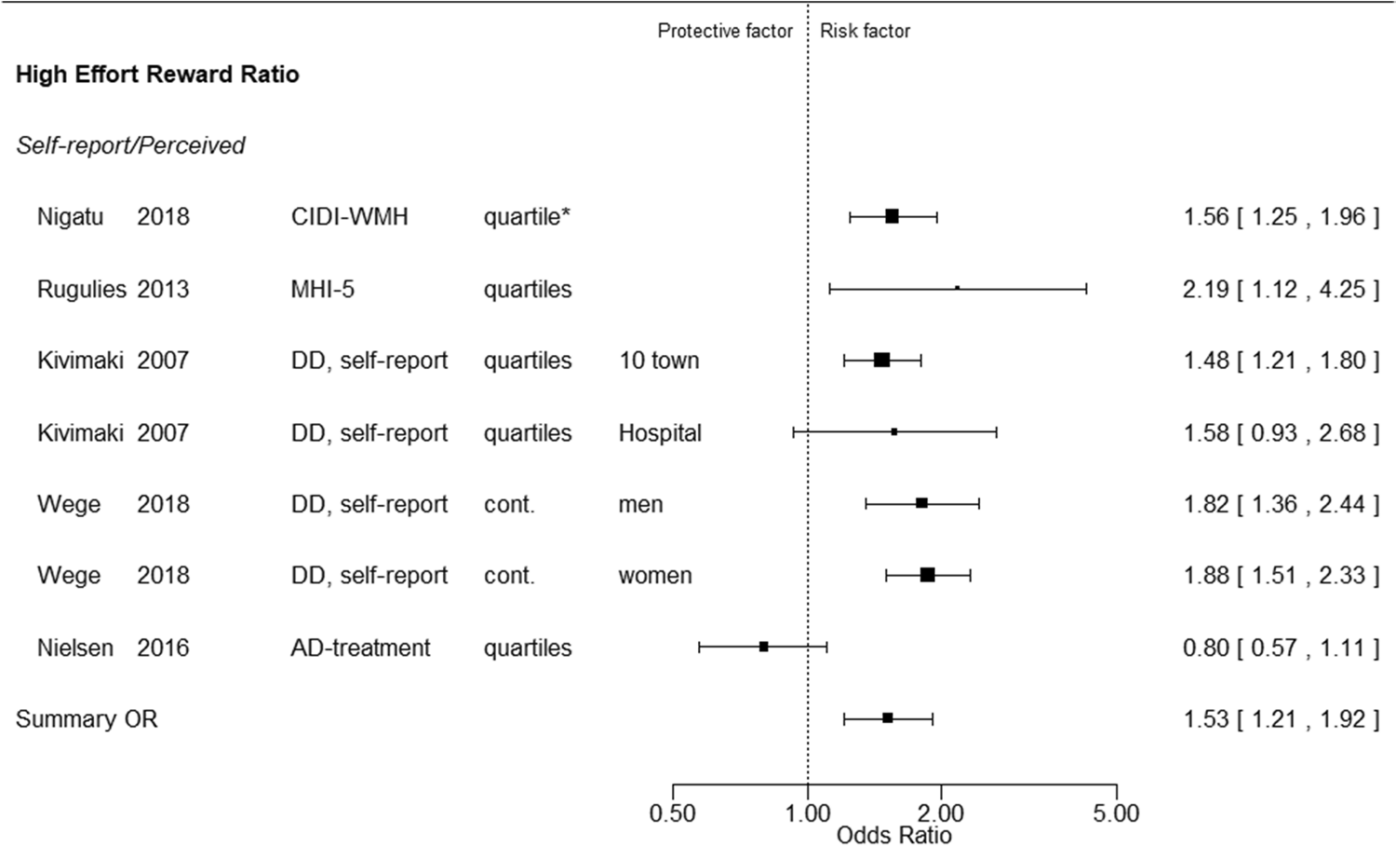
Forest plot of effort-reward-imbalance. Column explanations and abbreviations, see legend to Fig. 1. Additional abbreviations: MHI-5: Mental Health Inventory; quartiles: upper quartile versus lowest quartile; cont.: continuous, by scale unit

Relative risk estimates for the relation between specific exposures and measures of depression are shown in Forest plots, ordered by method of exposure and outcome assessment. We exhibit two example plots (job strain (Fig. 1) and effort-reward-imbalance (Fig. 2)) here, and remaining plots in Supplementary material, Appendix 3 (Forest plots (Figures 1 to11)).

The Forest plots demonstrate that associations between exposures and outcomes were characterized by heterogeneous measures referring to different units of exposure and exposure contrasts. We did not make any statistical tests to assess deviations from homogeneity because heterogeneity was obviously present irrespective of the results of statistical tests.

In this situation meta-analytic summary estimates are less satisfactory as best evidence measures of the associations between exposures and outcome. The summary risk estimate for an exposure cannot be related to any single scale of that exposure and is liable to greater uncertainty than indicated by its 95% confidence limits. Nevertheless, such estimates may convey useful summary information about the direction, size and consistency of associations (see Discussion section).

We therefore opted to include meta-analytic summary risk estimates and their 95% confidence limits in the Forest plots, and to consider them in a cautious assessment of the average divergence from unity and of the consistency of results across individual studies.

We extracted 173 risk estimates for the relation between single exposure measurements and measures of depression (Table 2). Twenty-two were based on analyses with exposure as a continuous variable, 59 were based on the highest exposure category compared to the lowest category with exposures categorized into three of more categories, and 92 were based on a dichotomy of the exposure variable.

**Table 2:** Summary description of forest and funnel plots

Exposure	Number of studies	Number of risk estimates/significant estimates/ exposure-response pattern	Formal summary of risk estimates	95% confidence limits of formal summary of risk estimates	Consistency of risk estimates^a^	Methods used to assess exposure (number of risk estimates)		Methods used to diagnose depression (number of risk estimates)			Adjustment for five or more out of ten potentially important confounders (number of studies/risk estimates)	Funnel plots, possible reporting bias
						Self-report	Work-unit average, job-exposure matrix, register information	Clinical interview or questionnaire	Anti- depressant treatment	Hospital discharge diagnosis		
Demands	16	22/5/2	1.08	0.98–1.19	High	18	4	9	10	3	6 / 7	Negative
Control	10	13/2/1	1.07	1.01–1.13	High	9	4	4	6	3	2 / 2	Positive
Decision authority	7	9/1/0	0.93	0.77–1.13	Moderate	8	1	5	3	1	4 / 5	Negative
Skill discretion	8	11/2/1	1.12	0.98–1.29	Moderate	10	1	5	5	1	4 / 5	No
Job strain	9	12/4/3	1.14	1.05–1.25	High	9	3	5	4	3	0 / 0	Positive
Support, unspecified	5	7/2/1	1.14	0.98–1.32	Moderate	6	1	4	3	0	2 / 3	Positive
Support, colleagues	6	9/3/0	1.37	1.07–1.74	Low	9	0	5	3	1	0 / 0	No
Support, supervisors	6	9/2/0	1.33	1.11–1.60	Moderate	9	0	5	3	1	0 / 0	Negative
Effort-reward-imbalance	6	7/5/4	1.53	1.21–1.92	Low	7	0	6	1	0	2 / 2	Positive
Job insecurity	7	9/5/1	1.35	1.21–1.50	High	9	0	8	1	0	2 / 2	Positive
Emotional demands	6	13/3/0	1.21	1.08–1.36	High	8	5	7	4	2	4 /9	Negative
Procedural injustice	4	4/1/1	1.23	1.02–1.47	Moderate	3	1	4	0	0	1 / 1	No
Relational injustice	4	4/3/3	1.60	1.14–2.24	Low	3	1	4	0	0	1 / 1	Positive
Night work	4	6/0/0	1.15	1.01–1.30	High	6	0	6	0	0	1 / 1	No
Shift work	6	9/0/0	1.10	0,98–1.23	High	9	0	6	3	9	1 / 1	No
Violence	4	8/4/2	1.40	1.28–1.52	High	4	4	0	4	4	0 / 0	Positive
Bullying	4	4/3/2	2.58	1.13–5.93	Low	3	1	4	0	0	1 / 1	No
Work load	3	3/1/1	1.24	0.95–1.61	Low	1	2	1	2	0	1 / 1	No
Working hours^b^	11	14/5/1	1.07	0.98–1.18	High	14	0	16	11	0	2 / 2	Negative

^a^Ratio between highest and lowest confidence limits of formal summary of risk estimates: < 1.30 = high, 1.30- < 1.50 = moderate, > 1.50 = low

^b^Working hours was reported as 27 risk estimates. To compare with other studies, we only considered high vs low as a risk estimate in this table, in all 14 such risk estimates

Among the 173 risk estimates, 51 were statistically significant, and for 22 of those risk estimates there was a monotonic increase of risk with increasing exposure (six risk estimates based on exposure analysed as a continuous variable, and 17 analysed for at least three exposure categories). Among the specific exposures examined demands, job strain, violence and bullying each exhibited an exposure–response relationship for two risk estimates, relational injustice for three, and effort-reward-imbalance for four. However, no specific exposure showed an exposure–response gradient for more than one risk estimate that was based on independent assessment of exposure and outcome.

Table 2 summarises the main findings from Forest and Funnel plots of the 19 exposures that were assessed. The formal summary risk estimates exceeded unity for all exposures except decision authority, and fell between 0.93 and 1.60 except for bullying (2.58). The consistency of individual risk estimates was assessed from the ratio between their upper and lower 95% confidence limits (range 1.12 to 1.96, except 5.25 for bullying) and arbitrarily categorized as high (< 1.30), moderate (1.30–1.50) or low (> 1.50) for nine, five and five exposures, respectively (see Table 2).

Table 2 also shows a summary measure of adjustment for potentially important confounders (excluding age and sex) that were identified a priori, classified according to the number of studies and risk estimates that adjusted for at least five of the factors. Finally, Table 2 shows whether Funnel plots (not presented) indicated bias that favoured publication of risk estimates above or below unity.

### Repeated exposure and changes in exposure

A few studies with several examination rounds examined effects of stable and changing exposures [12, 26, 94, 96, 105], see Table 3. Two studies reported risk increase with the number of examination rounds at which high job strain was present [12, 26]. However, risk estimates for changing job strain levels were inconsistent, and for one study, the risk estimate for a change from low to high job strain was similar to that for repeated high job strain [105], raising doubts about the interpretation of effects of repeated high job strain in the other studies. Effects of repeated or changing levels of demands, control and support were each examined in only one or two studies.

**Table 3:** Effects of repeated and changed exposures

Exposure, first author year	Diagnostic method^a^	Exposure contrast^a^	High level, number of examination rounds	OR	95% CI	
*Job strain*						
Stansfeld 2012 [12]	UM-CIDI	Tertile*	None of the times	1 (ref.)		
			1 time	1.28	0.84	1.95
			2–3 times	1.49^2^	0.98	2.27
Madsen 2017 [26]	DD, hospital	Quadrant (2a)	None of the times	1 (ref.)		
			1 time	1.23	0.88	1.71
			2 times	1.56^c^	0.99	2.45
*Support*						
Stansfeld 2012 [12]	UM-CIDI	Tertile*	None of the times	1 (ref.)		
			1 time	0.97	0.64	1.49
			2–3 times	1.16	0.77	1.74
*Job strain*^d^			**Level/change, first and second examination**			
Stansfeld 2012 [12]	UM-CIDI	Tertile*	Low and low	1 (ref.)		
			High and low	1.55	0.97	2.48
			Low and high	1.67	1.04	2.67
			High and high	1.94^b^	1.22	3.08
Madsen 2017 [26]	DD, hospital	Quadrant (2a)	Low and low	1 (ref.)		
			High and low	1.12	0.66	1.89
			Low and high	1.22	0.77	1.94
			High and high	1.63^c^	0.99	2.68
Wang 2009 [105]	CIDI-SFMD	Job strain ratio, > 1	Low and low	1 (ref.)		
			High and low	0.97	0.61	1.53
			Low and high	1.60	1.00	2.57
			High and high	1.52	1.00	2.30
Smith 2012 [96]	CIDI_SFMD	Job strain ratio change	Unchanged	1 (ref.)		
			Increased	1.24	0.57	2.68
			Decreased	1.17	0.50	2.74
*Demands*						
Smith 2012 [96]	CIDI-SFMD	Demands change	Unchanged	1 (ref.)		
			Increased	2.36	1.14	4.88
			Decreased	1.04	0.12	8.66
*Control*						
Smith 2012 [96]	CIDI-SFMD	Control change	Unchanged	1 (ref.)		
			Increased	1.11	0.60	2.06
			Decreased	0.93	0.52	1.66
*Support*						
Smith 2012 [96]	CIDI-SFMD	Support change	Unchanged	1 (ref.)		
			Increased	1.33	0.76	2.33
			Decreased	0.45	0.17	1.21

^a^Abbreviations: see legends to Figs. 1 and 2

^b^The different risk estimates of repeatedly high job strain in this study is due to different adjustments (the first risk estimate is adjusted for distress symptoms at baseline, the second is not)

^c^The different risk estimates of repeatedly high job strain in this study is due to different materials (the first risk estimate is based on a larger material than the second)

^d^Results from a study of Shield 2006 [94] is not included because of overlap with the study of Wang et al. 2009[105]

### Validity of measures of depression

Analysis of available data indicated that for diagnoses based on fully structured diagnostic interviews and questionnaire instruments, the false positive rate in a general population sample with a 5% prevalence of depressive episodes would be in the order of 60% to 90%, and the false negative rate below 3% when compared to diagnoses based on semi-structured clinical interviews (Supplementary material, Appendix 4).

Corresponding estimates for primary care diagnoses of depression were false positive rates in the order of 80% to 90% and false negative rates of < 5%. However, population studies indicate that fewer than half of people with a depressive episode access the health care system about their depressive symptoms [51, 52], adding to this level of diagnostic misclassification.

The accuracy of self-reported doctor’s diagnosis of depression as a marker for depressive episodes may be further compromised by errors in patient recall.

Antidepressant treatment is mainly prescribed in primary care. Population studies indicate that fewer than half of cases with a depressive episode are treated with antidepressants [53]. and that depressive symptoms are the indication for treatment in fewer than half of primary care patients in whom such medication is prescribed [54].

Only a small proportion of persons with a depressive episode are referred to hospital for assessment and treatment, and routine hospital discharge diagnoses of depressive episodes also suffer from misclassification [115–118].

Thus, available data suggest that self-reports of doctor’s diagnoses of depression, antidepressant treatment and hospital discharge diagnoses of depressive episodes may suffer from substantial inaccuracy as measures of depressive episodes in the general or working population (for further details and documentation, see Supplementary material, Appendix 4).

### Assessment of completeness of reporting

The average sum score for completeness of reporting was 6.3 (range 5–7) for SM and 5.5 (range 3–7) for JPB. We decided not to pursue this difference further, because we agreed on a generally high reporting quality, and other factors (validity of outcome, adjustment for confounding and common method bias) were more important for assessment of quality and interpretation of study results.

## Discussion

The purpose of our review was to evaluate the strength of epidemiological evidence that long-lasting stress at work causes the development of depressive disorders. The assessment was needed to help decide whether depression should be designated as a compensable occupational disease in Denmark, but could also be useful to policy-makers in other countries. It depended not only on the statistical significance of the associations that were found, but also on their strength (in terms of relative risk), consistency across studies, robustness to possible bias and confounding, and the nature of any reported exposure–response relationships.

We identified 54 studies on the relationship between 19 psychosocial factors at work and 11 measures of depression. The studies pertaining to each specific exposure differed substantially in the methods by which exposure was assessed and depression ascertained, and in control of possible confounding. In this situation the use of meta-analytic summary estimates in the interpretation of the results is uncertain. Unfortunately, we have no formal means to define the level of homogeneity for each specific exposure. As a starting point we have interpreted the meta-analytic summary results as if they were potentially useful, taking into account that they are liable to greater uncertainties than are indicated by their confidence intervals. This is a best case scenario since the alternative would be no interpretation in terms of causality.

The summary risk estimates were above unity for all but one of the 19 exposures examined. They varied between 0.93 (low decision authority) and 1.60 (low relational justice) except for one outlier (2.58, bullying), and were below 1.25 for 12 of 19 exposures.

The question to be answered is whether these generally positive associations are likely to reflect causal effects of occupational psychosocial factors on measures of depression or could be explained by a combination of random sampling error, residual confounding and bias.

Relative risks of the magnitude that were observed could arise from fairly minor degrees of bias related to study design and execution, even if established confounders were controlled for.

Random sampling error alone is unlikely to be an explanation where the lower 95% confidence for a risk estimate exceeds one. This was formally the case for 11 of the exposures, but is most likely the case for fewer, because formal confidence limits overestimated the true precision of the risk estimates.

### Dependence between assessment of exposure and outcome

One possible reason for bias is that exposures were self-reported in 44 studies, and perceptions of exposure may have been influenced by personal characteristics that also made an individual more likely to report or present with symptoms of depression. Thus, their risk estimates may have been inflated due to common method bias [119–121].

A few studies tried to reduce this type of bias by temporal separation of the assessment of exposure and outcome [96], by examining exposures defined by job content rather than perceived exposures [99], or by adjustment for aspects of personality [50, 99], or baseline symptoms of distress [12]. However, the effectiveness of such approaches is uncertain.

Many longitudinal studies have demonstrated reverse and reciprocal associations between variables which were first considered to be exposure and outcome variables. Thus, it has been convincingly demonstrated that depressive symptoms at baseline may longitudinally predict poorer level of self-reported psychosocial factors at work [122–124]. Some studies included in this review examined and confirmed the presence of longitudinally reversed temporal relations [26, 50, 83]. The underlying mechanisms are complex and may reflect real changes in the work environment following depression (e.g. selective job change), or within-person changes in perceptions of an unchanged work environment [125]. Such reverse causation may have inflated some risk estimates.

### Exposure

Only one study used an objective measure of exposure (hospital bed occupancy above the norm as a measure of work load in hospital staff), and that was strongly associated with antidepressant treatment [102]. All other exposure measures were based on individual perceptions of the work environment and may not have accurately reflected objective work conditions. This includes work-unit and job-exposure-matrix average exposures, but these measures are independent or nearly independent of individual outcomes, and in this circumstance, the expected effect of the misclassification of exposures would be to bias risk estimates towards the null. This would also be the case for individual self-reported exposures if variation due to common method bias could be controlled. The level of misclassification of self-reported psychosocial exposure cannot be assessed in the absence of objective measures with which to compare, and such data are few or absent.

The heterogeneity of exposure measures, their distribution characteristics and category cut points makes it difficult to compare results from different studies. This problem is further aggravated by very different parameterizations in the analyses (see Forest plots (Figs. 1, 2 and Appendix 3 (Figures A3.1-A3.11)). These limitations in exposure specifications and analyses will have tended to increase the variance of effect estimates and to produce greater inconsistency between studies.

Workers are simultaneously exposed to a multitude of psychosocial factors at work. Most studies considered effects of a single exposure or only a small number of exposures. A few studies examined multiple factors simultaneously in their analyses, but data were too sparse to evaluate the relative strengths and combined effects of specific exposures. Studies on job strain examine the combined effect of demands and control, and studies of effort-reward-imbalance examine the combined effect of efforts and rewards. Unfortunately, the effects of these composite exposures were mostly reported without adjustment for the effects of their components, and their reported combined effects may therefore be biased or due to only one of the two components. For job strain, main effects of demands and control and their multiplicative interaction were sometimes examined in preliminary or sensitivity analyses. The job strain theory of an interaction between the two components was not confirmed in these analyses [11, 26, 77, 126].

### Outcome

Only three studies diagnosed depressive episodes exclusively by a semi-structured diagnostic interview, which we have considered as the standard against which other methods should be assessed in an epidemiological context (see Supplementary material, Appendix 4). In comparison, measures of depression based on fully standardized diagnostic interviews, questionnaires, self-reported doctor’s diagnoses of depression, antidepressant treatment and routine hospital diagnoses are liable to misclassification, leading to both false positives and false negatives. This misclassification could bias the associations between exposures and depressive episodes, especially if the false positives were people with less serious depressive symptoms that could be caused by psychosocial factors at work.

We explored the potential effects of this misclassification in separate analyses. The results indicated that in the general population false positive rates were likely to exceed 45%, often substantially, for all diagnostic methods except routine hospital diagnoses, for which the rate might be close to 25%. False positives are mainly persons with depressive symptoms which do not fully meet the criteria for a depressive episode. False negative rates were below 3% for diagnoses based on fully structured interviews and questionnaire instruments and higher or undefined for other diagnostic methods.

Further analyses showed that under circumstances similar to those of many studies included in this review, it is plausible that relative risks up to 1.50 could occur in the absence of any true association between depressive episodes and the exposure, if the exposure was associated with false positives (Supplementary material, Appendix 4).

Considering that international classifications of mental disorders maintain a clear distinction between depressive episodes and depressive symptoms in different constellations or of less severity, we find it reasonable to consider that depressive episodes could be differently associated with psychosocial factors at work than depressive symptoms that do not fulfil diagnostic criteria for a depressive episode. Self-reported depressive symptoms could, for example, be more sensitive to common method bias than depressive symptoms elicited by psychiatric clinical experts to decide on a diagnosis of a depressive episode. However, we are not aware of data that support or challenge this hypothesis.

We conclude that it is not unlikely that positive associations between psychosocial factors at work and measures of depression with high false positive rates could be inflated and possibly even explained by an association with subthreshold depressive symptoms.

Furthermore, there is evidence that in the general population, fewer than half of people with depressive episodes utilize the formal health care system for their depressive symptoms [51, 52], and even smaller proportions receive antidepressant treatment [53, 54] or are referred for hospital assessment and treatment. Studies based on measures of depression defined by self-reported doctor’s diagnoses, antidepressant treatment or hospital discharge diagnoses may not be representative of depressive episodes in the general population, which could differ in their risk factors. In particular, associations with psychosocial factors at work could reflect an increased tendency to seek professional help because depressive symptoms become worse in the presence of adverse working conditions or because adverse working conditions are experienced as worse in the presence of a depressive episode.

Many studies failed to assess baseline depression status or only adjusted for its effects, which meant that cases identified during follow-up comprised a mixture of first and recurrent depressive episodes. Risk factors for first and recurrent depressive episodes may differ or carry different levels of risk [37, 127–129]. Whether this applies to psychosocial factors at work is uncertain but not implausible.

### Confounding

Previous and current depressive episodes and depressive symptoms are strong risk factors for new depressive episodes and likely to be associated with self-reported psychosocial exposures at baseline [121, 122]. They should therefore be considered strong potential confounders of the relation between exposure at baseline and depressive episodes at follow-up. However, only 14 cohort studies controlled for at least two of the three factors. In the other cohort studies, the population followed is likely to have included a number of participants with current or previous depressive episodes at baseline. Such individuals may be more likely than other participants to report more negatively on the psychosocial work environment at baseline, and also carry a higher risk of subsequently being recorded as having a depressive episode, contributing a significant proportion of all “new” cases during follow-up.

Age and sex were controlled for in all but one study, but fewer than half of studies controlled for at least four of ten other potentially important confounders and fewer than a quarter controlled for five or more. Thus, residual confounding is not unlikely.

One can speculate whether the factors which we have considered as potential confounders could be antecedent to the psychosocial risk factors of interest or mediators of their effects and whether effects might be bi-directional [122]. Since the purpose of this review was to assess whether the evidence supports a causal role for psychosocial factors at work in relation to depressive episodes, we took the conservative approach of considering them as unidirectional potential confounders that should be controlled for by exclusion or adjustment.

### Exposure–response associations and effects of change in exposure intensity

Among studies with exposure measurement at a single point in time most exposure–response relations were liable to common method bias. A few were based on independent assessment of exposure and outcome, but this did not apply to more than a single exposure–response relation for any psychosocial risk factor.

Studies of repeated exposures were few and seemed inconsistent with respect to effects of repeated high exposures compared to changing exposures (Table 3). Other than in the study of Smith et al. [96], the time spans between repeated exposures and measures of outcome covered many years.

Altogether, available data on exposure–response relations for intensity of single exposures or repeated exposures did not contribute significantly to the interpretation of causal relationships between specific exposures and depressive episodes.

### Publication bias

Funnel plots suggested publication bias for control, job strain, support (unspecified), effort-reward-imbalance, job insecurity and relational injustice (Table 2). In addition, there is a possibility that in some cases, researchers selectively reported findings that were more positive. For example, one study examined effects of different job strain parametrizations and chose to report results for those showing the strongest association with the outcome [112]. Without further information, it is difficult to gauge the extent of such bias, which will have tended to inflate risk estimates.

### Sensitivity to exclusion of studies with high prevalence of depression

We excluded studies in which the point or 12-month period prevalence of depression, as measured, exceeded 10%, because we judged that such a high prevalence of true depressive episodes was unlikely in an unselected working population, and would suggest serious diagnostic misclassification. A total of eight studies were excluded only for this reason, of which six provided risk estimates in relation to exposures listed in Table 2. To check whether the exclusion might have impacted importantly on our conclusions, we examined these six studies in more detail. For each of seven exposures on which they presented findings, risk estimates tended to be a little higher than the corresponding summary risk estimate in Table 2. However, in four of the studies, including that with the highest risk estimates, the prevalence of depression was close to 25% (see Supplementary material, Appendix 2), which seems implausibly high for working populations, and suggests a high rate of false positive diagnoses. As already discussed, such misclassification could lead to spurious inflation of risk estimates. Thus, we do not think that the findings call into question the balance of evidence from the studies that met our inclusion criteria.

### Conclusion on causality for specific exposures

Considering that our formal meta-analytic risk estimates are liable to greater uncertainty than their 95% confidence limits indicate, and that there is a potential for inflationary bias due to lack of independent assessment of exposure and measures of depression, we cannot conclude with confidence that an exposure for which the lower 95% confidence limit of the formal summary risk estimate was below or close to unity is a likely cause of depressive episodes or recurrent depressive disorders. This conclusion applies to *demands, decision authority, skill discretion, support (unspecified), shift work, work load and work-hours* (below unity)*,* and to *control, job strain, procedural injustice and night work* (close to unity).

Among the remaining exposures, the consistency of risk estimates was low for *support (colleagues), effort-reward-imbalance, relational injustice and bullying.* For *support (colleagues)* and *effort-reward-imbalance,* exposures were all self-reported and the level of confounder control was low. For *relational justice*, there were only four risk estimates, of which three were based on self-reported exposure. One risk estimate based on work-unit average was statistically significant, based on a semi-structured clinical interview, and had a high level of confounder control. However, more than one high quality study is needed for causal inference. For *bullying,* there were only four risk estimates, of which three were based on self-reported exposure, finding high and statistically significant risk estimates. However, control of confounding was low in these studies, and the results in one of the studies could have been due to a cross-sectional association because bullying at follow-up was included in the exposure assessment [83]. One study was based on work-unit average exposure, had a high level of confounder control and found no association between bullying and measures of depression. A study of self-reported negative acts, which may be related to bullying, had a high level of confounder control and found no association with depression.

For *support (supervisors)* and *job insecurity*, exposures were all self-reported and the level of confounder control was low.

For *emotional demands,* eight out of 13 risk estimates were based on self-reported exposure, out of which four studies used antidepressant treatment as their outcome. Three estimates were based on work-unit average exposure, had a high level of confounder control and found no association. Two estimates (men and women) from one study were based on exposure assessed from a job-exposure-matrix and hospital discharge diagnoses of affective disorders. The level of confounder control was low. These risk estimates were higher than unity, and because of the size of this study their results had a strong influence on the formal summary risk estimate for all studies. It should be noted also, that six out of 13 estimates were from the same study.

For *violence/threats of violence,* exposure was based on self-report for four risk estimates, two of which were from the same study, although referring to violence and threats of violence, respectively. Risk estimates for these two violence modalities are unlikely to be independent. Four risk estimates, all from the same study, were based on a job exposure matrix and the outcome was a hospital discharge diagnosis of affective disorders, with estimates for violence and threats of violence for each sex. The level of confounder control was low. These risk estimates were higher than unity, and because of the size of the study they had a strong influence on the formal summary risk estimate.

Exposure–response associations of depression with exposure intensity, stability or change were relatively few and did not contribute to the assessment of causality since most of them could be inflated by common method bias or the pattern for stable and changed exposures was inconsistent.

The limitations mentioned above in relation to each exposure were not the only ones. Misclassification of outcome, poor confounder control or indications of potential publication bias could be added for several exposures. The net effect of all these limitations is that there were too few studies for which it seemed unlikely that increased risk estimates could not be explained by chance, bias or residual confounding.

The evidence for a causal association between any of these exposures and depressive episodes, whether first or recurrent episodes, is therefore limited, and for the same reason a causal association cannot be excluded.

### Practical implications and suggestions for future research

The level of support for a causal association has practical implications. In the clinical context, it must be taken into account when communicating with patients about the nature of their illness and forming recommendations on sick leave or job change. In a political/administrative context it is important for decisions on preventive strategies, compensation of illnesses as occupational disorders, and the prioritisation of further research.

We suggest that future studies use methods that enable independent assessment of exposure and depressive episodes; that the outcome be measured by a semi-structured clinical interview; and that exposure–response relations be studied across increasing levels of exposure rather than simply dichotomizing exposures. We further suggest that follow-ups be carried out at short time intervals in order to catch all new cases of depressive episodes and that the study designs clearly accommodates the need to distinguish first-onset from recurrent depressive episodes. Important potential confounders should all be assessed as accurately as possible and feasible.

#### Strengths and limitations

The main strengths of this review are its broad coverage of the peer-reviewed literature on potential psychosocial risk factors for depression in the workplace, and its attention to uncertainties from the possibility of bias and residual confounding. Limitations include its restriction to English language papers and exclusion of “grey” literature. However, it seems unlikely that these could importantly compromise its conclusions.

## Conclusion

Taking statistical uncertainties and the potential for bias and confounding into account, it is not possible to conclude with confidence that any of the psychosocial exposures at work included in this review is a likely cause of depressive episodes and recurrent depressive disorders. Nor is there sufficient evidence to conclude that a causal relationship for any of the exposures is unlikely.

## Supplementary information

Below is the link to the electronic supplementary material.

Supplementary file1 (PDF 1270 kb)

## Data Availability

References cited are available and accessible to the public.

## Abbreviations

CES-D: Center for Epidemiologic Studies Depression Scale
CIDI: Composite International Diagnostic Instrument
CIDI-SFMD: Short Form version of CIDI
CIDI-WMH: WHO Mental Health version of CIDI
HADS_D: Hospital Anxiety and Depression Scale, Depression subscale
MINI: Mini-International Neuropsychiatric Interview
MDI: Major Depression Inventory
MHI-5: Mental Health Inventory-5 items
PHQ-9: Patient Health Questionnaire-9 items
SCAN: Schedules for Clinical Assessment in Neuropsychiatry
UM-CIDI: University of Michigan version of CIDI
Zung SDS: Zung Selfrating Depression Scale
