# Supplementary material for: Are depressive disorders caused by psychosocial stressors at work? A systematic review with metaanalysis
Source: Eur J Epidemiol. 2021 Feb 12;36(5):479–96. doi: 10.1007/s10654-021-00725-9 (PMC8159794; doi:10.1007/s10654-021-00725-9)
Supplement: Supplementary file 1 — Supplementary file1 (PDF 1270 kb) [file 10654_2021_725_MOESM1_ESM.pdf]

Supplementary material

Are depressive disorders caused by psychosocial stressors at work? A systematic review with metaanalysis

Sigurd Mikkelsen[1], David Coggon[2], Johan Hviid Andersen[3], Patricia Casey[4], Esben Meulengracht Flachs[1], Henrik Albert Kolstad[5], Ole Mors[6] and Jens Peter Bonde[1]

[1] Department or Occupational and Environmental Medicine, Bispebjerg and Frederiksberg Hospital, University of Copenhagen, Copenhagen, Denmark

[2] MRC Lifecourse Epidemiology Unit, University of Southampton, Southampton, UK

[3] Department of Occupational Medicine, Danish Ramazzini Centre, University Research Clinic, Herning, Denmark.

[4] Department of Psychiatry, Mater Misericordiae University Hospital, Dublin, Ireland

[5] Department of Occupational Medicine, Danish Ramazzini Centre, Aarhus University Hospital, Aarhus, Denmark

[6] Department of Psychosis, Aarhus University Hospital – Psychiatry, Aarhus, Denmark

Corresponding author:
Sigurd Mikkelsen, ORCID: 0000-0003-0854-8276, Department or Occupational and Environmental Medicine, Bispebjerg and Frederiksberg Hospital, University of Copenhagen, Copenhagen, Denmark.
E-mail: Sigurd.Mikkelsen.01@regionh.dk

**Appendix 1**	**Literature search**	**page 2**
**Appendix 2**	**Flow chart and selection of studies**	**page 8**
**Appendix 3**	**Description of studies - tables and forest plots**	**page 16**
**Appendix 4**	**Validity of measures of depression**	**page 42**
**References**		**page 46**

Appendix 1. Literature search

1. PubMed 15.03.2019 (1,740 hits)

Search	Query	Items found
#5	#4 AND Filters:<br>Journal Article; Abstract; Publication date from 1980/01/01 to 2019/12/31; Humans; English; Adult: 19+ years	1740
#4	#1 AND #2 AND #3	2098
#3	**DESIGN ASPECTS**	3871179
	(“cohort studies”[MeSH Terms]) OR (“prospective studies”[MeSH Terms]) OR (“longitudinal studies”[MeSH Terms]) OR (“cross over studies”[MeSH Terms]) OR (“controlled clinical trial”[Publication Type]) OR (“cohort”[Title/Abstract]) OR (prospective[Title/Abstract]) OR (longitudinal[Title/Abstract]) OR (“cross sectional studies”[MeSH Terms]) OR (case-control studies[MeSH Terms]) OR (cross-sectional[Title/Abstract]) OR (cross sectional[Title/Abstract]) OR (case-control[Title/Abstract]) OR (case control[Title/Abstract]) OR (case-referent[Title/Abstract]) OR (case referent[Title/Abstract]) OR (“surveys and questionnaires/epidemiology”[MeSH Terms]) OR (survey[Title/Abstract]) OR (case-crossover[Title/Abstract]) OR (case crossover[Title/Abstract]) OR (“case-only”[Title/Abstract]) OR (intervention[Title/Abstract])	
#2	**ALL EXPOSURES**	4826310
	((job strain[Title/Abstract]) OR (job-strain[Title/Abstract]) OR (demand*[Title/Abstract]) OR (control[Title/Abstract]) OR (decision latitude[Title/Abstract]) OR (decision authority[Title/Abstract]) OR (skill discretion[Title/Abstract]) OR (isostrain[Title/Abstract]) OR (iso-strain[Title/Abstract]) OR (support[Title/Abstract]) OR (Karasek[Title/Abstract])) OR ((effort*[Title/Abstract]) OR (reward*[Title/Abstract]) OR (imbalance*[Title/Abstract]) OR (("effort-reward imbalance"[Title/Abstract]) OR "effort-reward-imbalance"[Title/Abstract]) OR (overcommitment[Title/Abstract]) OR (Siegrist[Title/Abstract])) OR ((organization*[Title/Abstract]) OR (organisation*[Title/Abstract]) OR (justice[Title/Abstract]) OR (injustice[Title/Abstract])) OR ((job insecurity[Title/Abstract]) OR (job security[Title/Abstract]) OR (downsizing[Title/Abstract]) OR (organisational change[Title/Abstract]) OR (organizational change[Title/Abstract]) OR (organisational restructur*[Title/Abstract]) OR (organizational restructur*[Title/Abstract])) OR ((work time[Title/Abstract]) OR (worktime[Title/Abstract]) OR (working time[Title/Abstract]) OR (work hours[Title/Abstract]) OR (workhours[Title/Abstract]) OR (working hours[Title/Abstract]) OR (work load[Title/Abstract]) OR (workload[Title/Abstract]) OR (working load[Title/Abstract]) OR (overtime[Title/Abstract]) OR (overload[Title/Abstract]) OR (time pressure[Title/Abstract]) OR (deadline[Title/Abstract])) OR ((conflict*[Title/Abstract]) OR (violence[Title/Abstract]) OR (threat*[Title/Abstract]) OR (bully*[Title/Abstract]) OR (harassment[Title/Abstract]) OR (mobbing[Title/Abstract])) OR ((emotional demands[Title/Abstract]) OR (emotional strain[Title/Abstract]) OR (social capital[MeSH Terms]) OR (social capital[Title/Abstract]) OR	

	(meaning*[Title/Abstract]) OR (resource*[Title/Abstract]) OR (person-related[Title/Abstract]) OR (caregivers[MeSH Terms]) OR (caregiver*[Title/Abstract])) OR (("shift work schedule"[MeSH Terms]) OR (shift*[Title/Abstract]))	
**#1**	**DEPRESSION AND (OCCUPATIONAL STRESS OR WORK-RELATEDNESS)**	7891
	((((("depression/classification"[MeSH Major Topic]) OR ("depression/diagnosis"[MeSH Major Topic]) OR ("depression/etiology"[MeSH Major Topic]) OR ("depression/prevention and control"[MeSH Major Topic]) OR ("depression/psychology"[MeSH Major Topic]) OR ("depression/statistics and numerical data"[MeSH Major Topic]) OR ("depression/epidemiology"[MeSH Major Topic]) OR ("depressive disorder/classification"[MeSH Major Topic]) OR ("depressive disorder/diagnosis"[MeSH Major Topic]) OR ("depressive disorder/epidemiology"[MeSH Major Topic]) OR ("depressive disorder/etiology"[MeSH Major Topic]) OR ("depressive disorder/prevention and control"[MeSH Major Topic]) OR ("depressive disorder/statistics and numerical data"[MeSH Major Topic]) OR ("depressive disorder/psychology"[MeSH Major Topic]))) NOT ("stress disorders, post traumatic"[MeSH Major Topic])) AND (((depression[Title/Abstract]) OR (depressive[Title/Abstract]) OR (antidepress*[Title/Abstract]) OR (anti-depress*[Title/Abstract])) NOT ((posttraumatic[Title/Abstract]) OR (post-traumatic[Title/Abstract]) OR (postpartum[Title/Abstract]) OR (pregnancy[Title/Abstract]) OR (perinatal[Title/Abstract]) OR (maternal[Title/Abstract])))) AND ((("occupational stress/analysis"[MeSH Terms]) OR ("occupational stress/classification"[MeSH Terms]) OR ("occupational stress/complications"[MeSH Terms]) OR ("occupational stress/diagnosis"[MeSH Terms]) OR ("occupational stress/epidemiology"[MeSH Terms]) OR ("occupational stress/etiology"[MeSH Terms]) OR ("occupational stress/prevention and control"[MeSH Terms]) OR ("occupational stress/psychology"[MeSH Terms]) OR ("occupational stress/statistics and numerical data"[MeSH Terms])) OR (("workplace/psychology"[MeSH Terms]) OR ("stress, psychological/adverse effects"[MeSH Terms]) OR ("stress, psychological/epidemiology"[MeSH Terms]) OR ("stress, psychological/psychology"[MeSH Terms]) OR ("work/adverse effects"[MeSH Terms]) OR ("work/psychology"[MeSH Terms]) OR ("occupations/adverse effects"[MeSH Terms]) OR ("occupations/psychology"[MeSH Terms]) OR ("occupations/epidemiology"[MeSH Terms]) OR (employment[MeSH Terms]) OR (work*[Title/Abstract]) OR (occupation*[Title/Abstract]) OR (job*[Title/Abstract]) OR (employment[Title/Abstract])))	

2. PsycNet 19.03.2019
(1,227 hits)

Search	Query	Items found
	**Depression AND (occupational stress OR work-relatedness) AND all exposures AND design aspects (with filters)**	1227
	((((((AnyField:(((Index Terms: (major depression)) OR (MeSH: (depressive disorder)) OR (MeSH: (depression))) AND -((MeSH: (stress disorders, post traumatic)) OR (Index Terms: (posttraumatic stress disorder)))))) AND (((((title:(depression)) OR (title:(depressive)) OR (title:(antidepress*)) OR (title:(anti-depress*))) OR ((abstract:(depression)) OR (abstract:(depressive)) OR (abstract:(antidepress*)) OR (abstract:(anti-depress*)))) AND - (((title:(posttraumatic)) OR (title:(post-traumatic)) OR (title:(postpartum)) OR (title:(pregnancy)) OR (title:(perinatal)) OR (title:(maternal))) OR ((abstract:(posttraumatic)) AnyField:(OR) (abstract:(post-traumatic)) OR (abstract:(postpartum)) OR (abstract:(pregnancy)) AnyField:(OR) (abstract:(perinatal)) OR (abstract:(maternal))))))) AND (((MeSH:(occupational stress))) OR ((IndexTerms:(occupational stress))) OR ((MeSH:(workplace))) OR ((IndexTerms:(psychological stress))) OR ((MeSH:(psychological stress))) OR ((MeSH:(work))) OR ((MeSH:(occupations))) OR ((MeSH:(employment))) OR ((title:(work*))) OR ((abstract:(work*))) OR ((title:(occupation*))) OR ((abstract:(occupation*))) OR ((title:(job*))) OR ((abstract:(job*))) OR ((title:(employment))) OR ((abstract:(employment))))))<br>AND<br>(((((title:(job strain)))) OR (((abstract:(job strain)))) OR (((title:(job-strain)))) OR (((abstract:(job-strain)))) OR (((title:("effort-reward imbalance")))) OR (((abstract:("effort-reward imbalance")))) OR (((title:("effort-reward-imbalance")))) OR (((abstract:("effort-reward-imbalance")))) OR (((title:(effort*)))) OR (((abstract:(effort*)))) OR (((title:(reward*)))) OR (((abstract:(reward*)))) OR (((title:(imbalance*)))) OR (((abstract:(imbalance*)))) OR (((title:(overcommitment)))) OR (((abstract:(overcommitment)))) OR (((title:(Siegrist)))) OR (((abstract:(Siegrist))))) OR ((((title:(organization*))) OR ((title:(organisation*)))) OR (((abstract:(organization*))) OR ((abstract:(organisation*)))) OR (((title:(justice))) OR ((title:(injustice)))) OR (((abstract:(justice))) OR ((abstract:(injustice)))) OR (((title:("job insecurity"))) OR ((title:("job security")))) OR (((abstract:("job insecurity"))) OR ((abstract:("job security")))) OR (((title:(downsizing)))) OR (((abstract:(downsizing))))) OR ((((title:("worktime"))) OR ((title:(worktime))) OR ((title:("working time"))) OR ((title:(workhours))) OR ((title:("working hours"))) OR ((title:("work load"))) OR ((title:(workload)))) OR (((abstract:("worktime"))) OR ((abstract:(worktime))) OR ((abstract:("working time"))) OR ((abstract:(workhours))) OR ((abstract:("working hours"))) OR ((abstract:("work load"))) OR ((abstract:(workload)))) OR (((title:(overtime))) OR ((title:(overload))) OR ((title:("time pressure"))) OR ((title:(deadline)))) OR (((abstract:(overtime))) OR ((abstract:(overload))) OR ((abstract:("time pressure"))) OR ((abstract:(deadline)))) OR (((title:(conflict*))) OR ((title:(violence))) OR ((title:(threat*))) OR ((title:(bully*))) OR ((title:(harassment))) OR ((title:(mobbing)))) OR (((abstract:(conflict*))) OR ((abstract:(violence))) OR ((abstract:(threat*))) OR ((abstract:(bully*))) OR ((abstract:(harassment))) OR ((abstract:(mobbing))))) OR ((((title:(emotional demands))) OR ((title:("emotional strain"))) OR ((title:("social capital")))) OR	

	(((abstract:(emotional demands))) OR ((abstract:("emotional strain"))) OR ((abstract:("social capital")))) OR (((title:(meaning*))) OR ((title:(resource*))) OR ((title:("person-related")))) OR (((abstract:(meaning*))) OR ((abstract:(resource*))) OR ((abstract:("person-related")))) OR (((title:(caregiver*)))) OR (((abstract:(caregiver*)))) OR (((title:(shift*)))) OR (((abstract:(shift*)))) OR (((IndexTerms:("social capital")))))))<br>AND<br>((((MeSH: (cohort studies))) OR ((title: (cohort))) OR((abstract: (cohort))) OR ((MeSH: (prospective studies))) OR ((Index Terms: (prospective studies))) OR ((title: (prospective))) OR ((abstract: (prospective))) OR((MeSH: (longitudinal studies))) OR ((Index Terms: (longitudinal studies))) OR((title: (longitudinal))) OR ((abstract: (longitudinal))) OR ((MeSH: (cross over studies))) OR ((MeSH: (controlled clinical trials)))) OR (((MeSH: (cross sectional studies))) OR ((MeSH: (case-control studies))) OR ((title: ("cross sectional")) OR(title: ("cross-sectional"))) OR<br>((abstract: ("cross sectional")) OR (abstract: ("cross-sectional"))) OR ((title: ("case-control")) OR (title: ("case control"))) OR ((abstract: ("case-control")) OR (abstract: ("case control"))) OR ((title: ("case-referent")) OR(title: ("case referent"))) OR ((abstract: ("case-referent")) OR (abstract: ("case referent"))) OR ((MeSH: (surveys)) AND (MeSH: (questionnaires))) OR ((title: (survey))) OR ((abstract: (survey))) OR ((title: ("case-crossover")) OR (title: ("case crossover"))) OR ((abstract: ("case-crossover")) OR (abstract: ("case crossover")))OR ((title: ("case-only"))) OR ((abstract: ("case-only"))) OR ((title: (intervention)))OR ((abstract: (intervention)))))<br>AND<br>Any Field: "Peer Reviewed Journal" AND Population Group: Human AND Age Group: Adulthood (18 yrs & older) AND Year: 1980 To 2019	

3. Web of Science Core Collection 21.03.2019, (2,025 hits)

# 26	(#21 AND #20) *AND* **LANGUAGE:** (English) *AND* **DOCUMENT TYPES:** (Article)<br>**Refined by: WEB OF SCIENCE CATEGORIES:** (PUBLIC ENVIRONMENTAL OCCUPATIONAL HEALTH)<br>*Indexes=SCI-EXPANDED, SSCI, A&HCI Timespan=1980-2019*	**2,025**
# 25	(#21 AND #20) *AND* **LANGUAGE:** (English) *AND* **DOCUMENT TYPES:** (Article)<br>*Indexes=SCI-EXPANDED, SSCI, A&HCI Timespan=1980-2019*	**10,078**
# 24	#21 AND #20<br>**Refined by: DOCUMENT TYPES:** (ARTICLE) AND **LANGUAGES:** (ENGLISH)<br>*Indexes=SCI-EXPANDED, SSCI, A&HCI Timespan=1900-2019*	**10,078**
# 23	#21 AND #20<br>**Refined by: DOCUMENT TYPES:** (ARTICLE)<br>*Indexes=SCI-EXPANDED, SSCI, A&HCI Timespan=1900-2019*	**10,475**
# 22	#21 AND #20 DEPRESSION AND EXPOSURES AND DESIGN ASPECTS<br>*Indexes=SCI-EXPANDED, SSCI, A&HCI Timespan=1900-2019*	**11,501**
# 21	#19 AND #10<br>*Indexes=SCI-EXPANDED, SSCI, A&HCI Timespan=1900-2019*	**23,793**
# 20	**TOPIC:** (cohort) *OR* **TOPIC:** ("controlled clinical trial") *OR* **TOPIC:** (prospective) *OR* **TOPIC:** (longitudinal) *OR* **TOPIC:** ("cross-sectional") *OR* **TOPIC:**("cross sectional") *OR* **TOPIC:** ("case-control") *OR* **TOPIC:** ("case control") *OR* **TOPIC:** ("case-referent") *OR* **TOPIC:** ("case referent") *OR* **TOPIC:** (survey) *OR* **TOPIC:** ("case-crossover") *OR* **TOPIC:** ("case crossover") *OR* **TOPIC:** ("case-only") *OR* **TOPIC:** (intervention)<br>*Indexes=SCI-EXPANDED, SSCI, A&HCI Timespan=1900-2019* DESIGN ASPECTS	**3,092,569**
# 19	#18 OR #17 OR #16 OR #15 OR #14 OR #13 OR #12 OR #11 EXPOSURES<br>*Indexes=SCI-EXPANDED, SSCI, A&HCI Timespan=1900-2019*	**9,364,301**
# 18	**TOPIC:** (shift*)<br>*Indexes=SCI-EXPANDED, SSCI, A&HCI Timespan=1900-2019*	**796,947**
# 17	**TOPIC:** ("emotional demands") *OR* **TOPIC:** ("emotional strain") *OR* **TOPIC:** ("social capital") *OR* **TITLE:** (meaning*) *OR* **TOPIC:** (resource*) *OR* **TOPIC:**("person-related") *OR* **TOPIC:** (caregiver*)<br>*Indexes=SCI-EXPANDED, SSCI, A&HCI Timespan=1900-2019*	**777,059**
# 16	**TOPIC:** (conflict*) *OR* **TOPIC:** (violence) *OR* **TOPIC:** (threat*) *OR* **TOPIC:** (bully*) *OR* **TOPIC:** (harassment) *OR* **TOPIC:** (mobbing)<br>*Indexes=SCI-EXPANDED, SSCI, A&HCI Timespan=1900-2019*	**614,189**
# 15	**TOPIC:** ("work time") *OR* **TOPIC:** (worktime) *OR* **TOPIC:** ("working time") *OR* **TOPIC:** ("work hours") *OR* **TOPIC:** (workhours) *OR* **TOPIC:** ("working hours") *OR* **TOPIC:** ("work load") *OR* **TOPIC:** (workload) *OR* **TOPIC:** ("working load") *OR* **TOPIC:** (overtime) *OR* **TOPIC:** (overload) *OR* **TOPIC:** ("time pressure") *OR* **TOPIC:** (deadline)<br>*Indexes=SCI-EXPANDED, SSCI, A&HCI Timespan=1900-2019*	**120,380**

# 14	**TOPIC:** ("job insecurity") *OR* **TOPIC:** ("job security") *OR* **TOPIC:** (downsizing) *OR* **TOPIC:** ("organizational change") *OR* **TOPIC:** ("organisational change") *OR* **TOPIC:** (organizational restructur*) *OR* **TOPIC:** (organisational restructur*) *Indexes=SCI-EXPANDED, SSCI, A&HCI Timespan=1900-2019*	**16,059**
# 13	**TOPIC:** (organization*) *OR* **TOPIC:** (organisation*) *OR* **TOPIC:** (justice) *OR* **TOPIC:** (injustice) *Indexes=SCI-EXPANDED, SSCI, A&HCI Timespan=1900-2019*	**779,452**
# 12	**TOPIC:** (effort*) *OR* **TOPIC:** (reward*) *OR* **TOPIC:** (imbalance*) *OR* **TOPIC:** ("effort-reward imbalance") *OR* **TOPIC:** ("effort-reward-imbalance") *OR* **TOPIC:**(overcommitment) *OR* **TOPIC:** (Siegrist) *Indexes=SCI-EXPANDED, SSCI, A&HCI Timespan=1900-2019*	**633,810**
# 11	**TOPIC:** ("job strain") *OR* **TOPIC:** ("job-strain") *OR* **TOPIC:** (demand*) *OR* **TOPIC:** (control) *OR* **TOPIC:** ("decision latitude") *OR* **TOPIC:** ("decision authority") *OR* **TOPIC:** ("skill discretion") *OR* **TOPIC:** (isostrain) *OR* **TOPIC:** ("iso-strain") *OR* **TOPIC:** (support) *OR* **TOPIC:** (Karasek) *Indexes=SCI-EXPANDED, SSCI, A&HCI Timespan=1900-2019*	**6,877,466**
# 10	#9 AND #8 DEPRESSION AND WORK-RELATEDNESS NOT (PTSD OR PREGNANCY) *Indexes=SCI-EXPANDED, SSCI, A&HCI Timespan=1900-2019*	**40,941**
# 9	#4 NOT #7 DEPRESSION NOT (PTSD OR PREGNANCY) *Indexes=SCI-EXPANDED, SSCI, A&HCI Timespan=1900-2019*	**464,481**
# 8	**TOPIC:** (work*) *OR* **TOPIC:** (occupation*) *OR* **TOPIC:** (job*) *OR* **TOPIC:** (employment) *Indexes=SCI-EXPANDED, SSCI, A&HCI Timespan=1900-2019*	**3,143,575**
# 7	#6 OR #5 *Indexes=SCI-EXPANDED, SSCI, A&HCI Timespan=1900-2019*	**717,959**
# 6	**TOPIC:** (postpartum) *OR* **TOPIC:** (pregnancy OR pregnant) *OR* **TOPIC:** (perinatal) *OR* **TOPIC:** (maternal) *Indexes=SCI-EXPANDED, SSCI, A&HCI Timespan=1900-2019*	**646,835**
# 5	**TOPIC:** (post-traumatic) *OR* **TOPIC:** (posttraumatic) *Indexes=SCI-EXPANDED, SSCI, A&HCI Timespan=1900-2019*	**73,272**
# 4	#3 OR #2 OR #1 DEPRESSION *Indexes=SCI-EXPANDED, SSCI, A&HCI Timespan=1900-2019*	**508,689**
# 3	**TOPIC:** (anti-depress*) *OR* **TOPIC:** (antidepress*) *Indexes=SCI-EXPANDED, SSCI, A&HCI Timespan=1900-2019*	**74,712**
# 2	**TOPIC:** (depressive) *Indexes=SCI-EXPANDED, SSCI, A&HCI Timespan=1900-2019*	**126,988**
# 1	**TOPIC:** (depression) *Indexes=SCI-EXPANDED, SSCI, A&HCI Timespan=1900-2019*	**426,983**

Appendix 2. Flow chart and selection of studies

This Appendix includes a flow chart of the literature search and information on reasons for excluding studies that were selected for full text reading. Some studies could have been excluded for more than one reason, but we only report one reason for each. We list excluded studies by reason for exclusion, and also by first author.

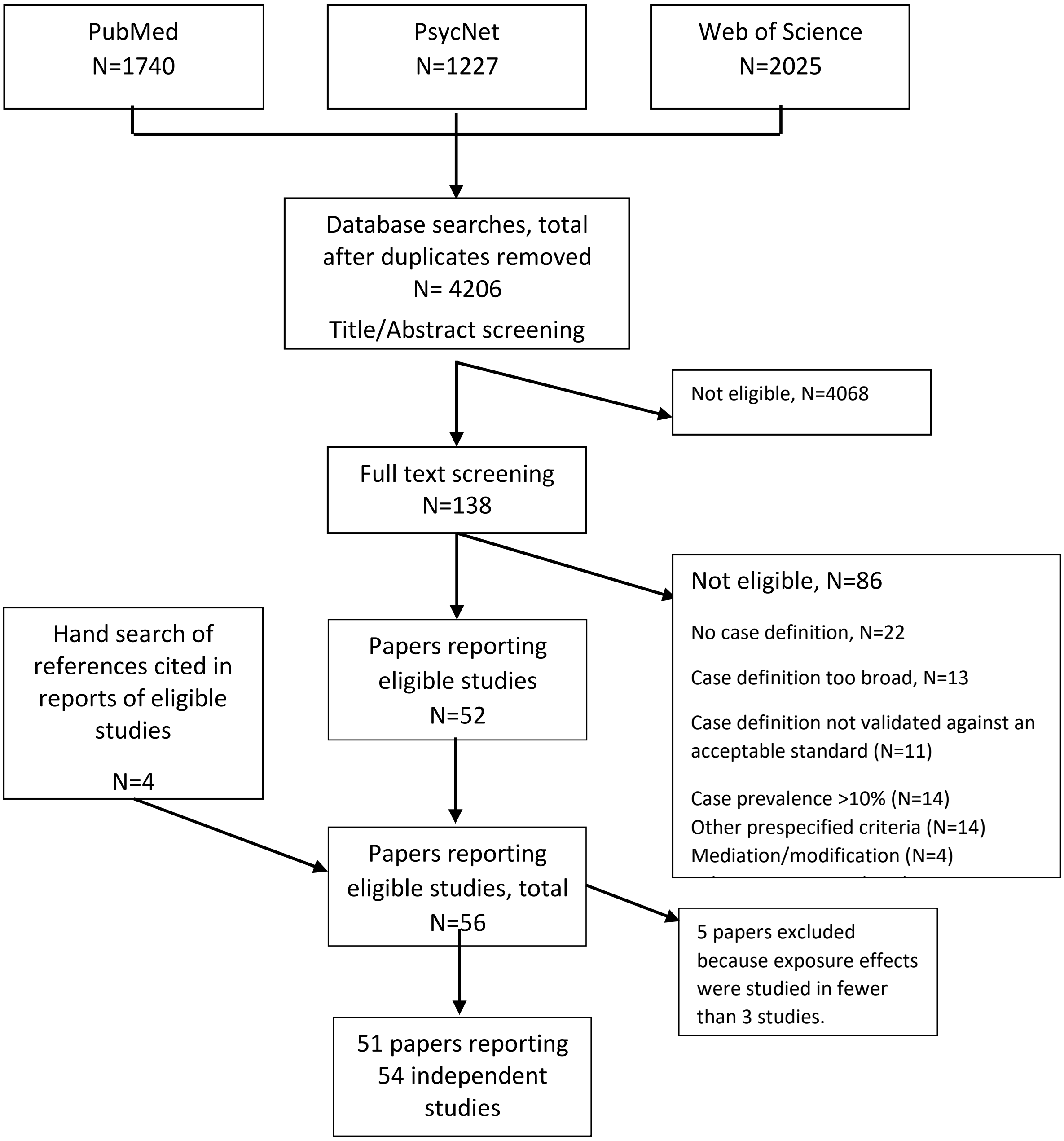

Flow-diagram for literature search

Excluded studies by reason for exclusion

1. *No case definition*

Twenty-two studies were excluded because there was no case definition indicative of a depressive disorder [1-22].

2. *Case definition too broad*

Thirteen studies were excluded because the case definition included subthreshold cases of depression (e.g. minor depression), mixed diagnoses (e.g. major depression *and* anxiety disorders), or non-specific case definitions (e.g. mental distress, use of psychotropic drugs) [23-35].

We accepted affective disorders (ICD F30-F39) or subsets thereof as a combined outcome because depressive episodes account for the large majority of cases in this diagnostic group [36, 37]. This was a post hoc decision and applied to four studies [38-41].

3. *Case definition not validated against an acceptable standard*

Eleven studies were excluded because their diagnostic instruments had not been validated against an acceptable standard method (semi-structured interview) for diagnosing depressive episode.

Seven studies used the Symptom Check List Core Depression (SCL-CD6) instrument to define depression cases [42-48].

One study used the General Well-being Schedule, subscale of depression [49].

One study used a single non-standard questionnaire item and the WHO Health and Work Performance Questionnaire) to define depression [50].

One study used a single non-standard questionnaire item (yes/no) to define depression [51].

One study used Spielberger's State-Trait Depression Scales [52].

4. *Case prevalence above 10% or unreported*

We excluded 14 studies of unselected working populations with depression prevalence at baseline above 10% because such a high prevalence in an unselected working population is unlikely (see Introduction section) and suggests a high rate of false positive diagnoses. If the baseline prevalence was not reported, we used the follow-up prevalence. Some characteristics for these studies are presented in Table A2.1. Of the 14 studies excluded because of high prevalence rates, six would have been excluded also for other reasons: overlap with other studies included in the review (Griffin 2002 [70], Niedhammer 1998 [78], and Virtanen 2015 [80]); mediation study or no usable risk estimates reported (Ahola 2007 [67], Amagasa 2012 [69]); or relating only to a risk factor (organizational change) for which there were too few studies for meaningful assessment (Netterstrom 2010 [77]).

5. *Other studies which did not fulfil predefined inclusion criteria.*

We excluded 14 other studies which did not fulfil inclusion criteria:

Three studies were excluded because they focused on exposures that were not included this review (social capital, but not work-related [53], weekend work [54], no relevant exposure specification [55]).

Table A2.1. Studies excluded from the review because of high baseline prevalence (%) of measures of depression. Prevalence rates in brackets are calculated or estimated from available information or cross-sectional follow-up panels if not specifically reported at baseline. M=male, F=Female.

First Author Year Reference	Diagnostic Instrument	Cut-off	Population	Prevalence
Ahola 2007 [67]	BDI SF form 13	5+	Dentists	27%
Amagasa 2012 [68]	CES-D 20	16+	Clerical workers	Not reported, most likely >10%[a]
Clays 2007 [69][f]	CES-D SF 11	80th percentile (score ≥19)	Middle-aged workers	25%
Griffin 2002 [70]	GHQ30, 4 items on depression	Upper quartile	Civil servants	(25%)[b]
Godin 2005 [71][f]	SCL-90, 16 items	Upper quartile	Workers in four enterprises	(25%)[b]
Kato 2014 [72][f]	CES-D 20	16+	Workers in two factories	24%
Kim 2017 [73][f]	CES-D 11	16+ (adjusted to CES-D 20)	General working population	(11%)[c]
Kim and Kim 2018 [74][f]	BDI II	20+	Automobile sales workers	16%
Meneton 2017 [75][f]	CES-D 20	M:17, F:23	Workers in a large company	24%
Nakata 2011 [76][f]	CES-D 20	16+ and 25+	Full-time employees	16+: 31%, 25+: 12%
Netterstrom 2010 [77]	MDI	20	Civil servants	(12 %)[d]
Niedhammer 1998 [78]	CES-D 20	M:17, F:23	Workers in a large company	(M: 25%, F: 28%)[e]
Tomioka 2011 [79][f]	CES-D 20	16+	Physicians	Total: 28%
Virtanen 2015 [80]	CES-D 20	16+	Civil servants	14%

[a] Prevalence of CES-D 20 score 16+ among employees in 20 randomly selected companies in Japan was 45%. [81]. All other studies but one in the present review, using the same criterion, had prevalence above 10%
[b] According to the quartile cut-off level.
[c] Based on information on supposedly successive exclusions (414/3790), page 664.
[d] Based on information on supposedly successive exclusions (82/685), page 446.
[e] 1-year follow-up prevalence, no information at baseline. The material overlaps with Meneton et al. 2017.
[f] Study excluded only because of high prevalence of depression

Two studies were excluded because their outcome measures were not included in this review (sickness absence [56] and administrative health claims [57]).

Seven studies were excluded because they were cross-sectional investigations of exposures other than shift work or work hours ([58-64].

One study was excluded because risk estimates were not reported with confidence limits or information allowing the calculation of such limits [65].

One intervention study was excluded because the intervention aimed to improve individual coping with work stress and not to reduce work stress [66].

6. ***Studies on effect mediation and effect modification***

We excluded four studies which specifically aimed to study whether the relation between an exposure and a measure of depression was mediated [82, 83] or modified by a third factor [84, 85]. We consider that such studies are seldom made or published if the exposure and the outcome are not associated in the absence of the third factor. The main associations between exposure and outcome may therefore be considered as selected results that are likely to inflate the overall association in reviews of main effects. The decision to exclude these studies was taken during the selection process. Two other studies examined whether there was effect modification by gender (Wege et al. 2018 [86]) and by occupational group (Rugulies et al. 2013 [87]). These studies were not excluded because we considered that the additional modification analyses followed naturally from the analyses of the main effects.

7. ***Selective reporting***

We excluded three studies due to selective reporting [88-90]. In the study by Siegrist et al. 2012 [88] on the association of depression with job strain variables and effort-reward-imbalance, the authors state: "With regard to the job strain model, the measurement was restricted to the control dimension. This decision was based on evidence that the predictive power of control exceeded the power of demand in several studies", supplied with a reference to an internal report on results in one of the sub-studies of that study. The reason for excluding effects of demands from the study indicates a prior knowledge of positive associations of depression with control and effort-reward-imbalance. We therefore excluded this study. The other two studies were based on the same material. The decision to exclude these studies was taken during the selection process.

8. ***Overlapping studies***

We excluded five studies because their material, exposure and outcome variables were partly overlapping with another study and contained no additional information of relevance for this review. One study by Oksanen et al. 2010 [91] was excluded due to overlap with a study by Kouvanen et al. 2008 [92]; one study by Sinokki et al. 2009 [93] was excluded due to overlap with another study by Sinokki et al. 2009 [94]; one study of Virtanen et al 2011 [95] was excluded due to overlap with another study by Virtanen et al. 2012 [96];one study by Wang et al. 2011 [97] was excluded due to overlap with a study by Shields 2006 [98] (see below); one study of Stoetzer et al. 2009 [99] was excluded due to overlap with a study by Fandino-Losada et al. 2013 [100].

We did not exclude overlapping studies if each of them contributed different relevant information. Where that occurred, we chose the results which in our view gave the most reliable information on the relation between exposure and the measure of depression.

Studies on bullying

We included overlapping studies on bullying [101-103] because exposure variables differed between these studies (bullying at work-unit level [101] or individual level [102, 103], bullying assessed by a general definition [101, 103] or by specific aspects of bullying [102].

Studies from the Canadian National Population Health Survey (CNHPS)

We included six partly overlapping studies from the longitudinal Canadian National Population Health Survey (CNHPS) [97, 98, 104-107]. The CNPHS has had seven examination cycles (Cycle 1:1994/1995, Cycle 2: 1996/97, Cycle 3: 1998/1999, Cycle 4:2000/01, Cycle 5:2002/3, Cycle 6: 2004/5, Cycle 7: 2006/07). Exposure was assessed by an abbreviated job content questionnaire (JCQ 12), which was administered at cycles 1, 4, 5 and 6. Depression during the previous 12 months was assessed by CIDI-SFMD interview at all cycles.

Two studies examined effects of support [98, 105]. We used results from Shields et al. 2006 [98] because support was specified as colleague and supervisor support, and for men and women separately.

Three of the studies examined effects of job strain on depression [97, 98, 104]. One reported the two-year incidence from cycle 1 to cycle 2 [104]. One reported on two pooled sets of baseline follow-ups (cycle 1 to 2 and cycle 4 to 5) [98]. One reported baseline job strain for cycle 4 and incident depression at cycle 5, 6 and 7 [97]. In all the three studies, adjustments were made for large sets of partly overlapping but not identical potential confounders. The results for the relation between job strain and depression were somewhat different for the three studies (Table A2.2). Neither of the two latter studies referred to any of the previous studies or discussed the different results. We chose the study of Shields et al. 2006 [98] to be included in this review because it had more observations than the Shields 1999 study [104] and a shorter time span between exposure assessment and the occurrence of the outcome than the study by Wang et al. 2011 [97].

Table A2.2 Associations between job strain at baseline and depression at follow-up (CIDI-SFMD) in three overlapping studies from the longitudinal Canadian National Population Health Survey (CNHPS). Odds Ratios (OR) and 95% Confidence Interval (CI)

**Association**	**Gender**	**Shields 1999** [104] **OR (95% CI)**[a]	**Shields 2006** [98] **OR (95% CI)**[b]	**Wang et al. 2011** [97] **OR (95% CI)**[c]
**Crude**	Men	not reported	3.3 (1.9-5.8)	not reported
	Women	not reported	2.0 (1.3-3.0)	not reported
**Adjusted**	Men	3.3 (1.3-8.5)	2.9 (1.5-5.4)	1.34 (0.87-2.06)
	Women	2.1 (1.1-4.0)	1.2 (0.8-1.9)	1.54 (1.17-2.03)

[a] Job-strain ratio upper quartile (>1.18) vs the three lower quartiles.
[b] Job-strain ratio high (>1.2) vs low (<0.8)
[c] Job-strain ratio >1.0 vs ≤1.0

Two CNPHS studies reporting on effects of stable and changing exposures had a similar design and were based on overlapping materials (Shields 2006 [98] and Wang et al. 2009 [106]). We report results only from the latest study [106].

Studies from the Canadian Alberta Cohort
One 1-year follow-up study of Wang et al., 2012 [108] overlapped another study on the same material but with four annual follow-ups (Nigatu et al 2018 [109]). We used the results for support and job insecurity from Wang et al., 2012 [108], because they were not reported in the study by Nigatu et al. [109], and results for job strain and effort-reward-imbalance from the study by Nigatu et al. [109] because of more annual follow-ups.

Studies from the Danish DWECS cohort
Use of antidepressants in relation to emotional demands was reported by Madsen et al. 2010 [110] for DWECS 2000, and by Hanson et al. 2013 [111] in a combined study of the Swedish SLOSH cohort and the DWECS 2005 cohort. We report DWECS-results on emotional demands only for the DWECS 2005 cohort.

Studies from the Whitehall II cohort
Virtanen et al (2012) [96] reported associations of job strain (quadrant analysis), social support and work hours measured in 1991-1993 (phase 3) with depression (UM-CIDI) in 1997-1999 (phase 5), adjusted for age and sex. Stansfeld et al 2012 [112] reported associations of job strain (difference analysis) and social support measured 1988-1993 (phase 1, 2 and 3) with depression (UM-CIDI) in 1997-1999 (phase 5), adjusted for a large set of potential confounders, including psychological distress (GHQ-30) at baseline. We

therefore included results for job strain and social support from the study by Stansfeld et al 2012 [112] and not from the study of Virtanen et al 2012 [96].

The study of Virtanen et al 2012 [96] reported results for the job strain quadrants “active jobs” (high demands in the absence of low control) and “passive jobs” (low control in the absence of high demands), adjusted for age and sex. These results reflect the mutually adjusted effects of high demands and low control (median splits) if they do not interact. We included these results because they have not been reported elsewhere. From this study we also report effects of work hours. It should be noted that the results for job strain differed markedly for these two Whitehall studies. In the study by Stansfeld et al. 2012 [112] job strain (upper tertile of the difference between demands and control compared to the lower tertile) in phase 3 was associated with UM-CIDI depression in phase 5 with an OR=1.96 (95% CI: 1.28 -3.00), adjusted for age and sex (Table 2 in that study). In the study of Virtanen et al. 2012 [96] the quadrant of high strain compared to low strain in phase 3 was associated with UM-CIDI depression in phase 5 with an OR=1.04 (95% CI: 0.46-2.39), adjusted for age and sex (Table 1 in that study).

Excluded studies by author
Table A2.3 shows reasons for exclusions (n=86) ordered by first author. Exclusion category refers to subheadings (numbered 1 to 8) specified above.

Table A2.3 List of studies excluded after full text reading (n=86). Reasons for exclusion. Sorted by first author. Exclusion category, see text and table footnote

	First author, year	Exclusion category	Reason for exclusion
1	Ahlin, 2018 [43]	3	Case definition not validated against an acceptable standard
2	Ahlin, 2018 [42]	3	Case definition not validated against an acceptable standard
3	Ahola, 2007 [67]	4	Case prevalence above 10%
4	Ahola, 2012 [66]	5	Intervention study to increase coping capability
5	Airila, 2014 [23]	2	Case definition too broad
6	Albrecht, 2017 [44]	3	Case definition not validated against an acceptable standard
7	Alterman, 2008 [58]	5	Cross-sectional, exposure not shift work or work hours
8	Amagasa, 2012 [68]	4	Case prevalence above 10%
9	Amagasa, 2013 [49]	3	Case definition not validated against an acceptable standard
10	Andrea, 2009 [24]	2	Case definition too broad
11	Andreeva, 2015 [45]	3	Case definition not validated against an acceptable standard
12	Andreeva, 2017 [46]	3	Case definition not validated against an acceptable standard
13	Barrech, 2017 [1]	1	No case definition
14	Berthelsen, 2015 [25]	2	Case definition too broad
15	Brenner, 2014 [47]	3	Case definition not validated against an acceptable standard
16	Burns, 2016 [26]	2	Case definition too broad
17	Butterworth, 2016 [27]	2	Case definition too broad
18	Clays, 2007 [69]	4	Case prevalence above 10%
19	de Lange, 2002 [2]	1	No case definition
20	DeSanto Iennaco, 2010 [57]	5	Outcome: administrative health claims
21	Dragano, 2008 [59]	5	Cross-sectional, exposure not shift work or work hours

22	Dragano, 2011 [89]	7	Selective reporting
23	Driesen, 2011 [50]	3	Case definition not validated against an acceptable standard
24	Einarsen, 2015 [28]	2	Case definition too broad
25	Emdad, 2013 [29]	2	Case definition too broad
26	Ferrie, 2005 [3]	1	No case definition
27	Fujiwara, 2008 [53]	5	No relevant exposure
28	Geiger-Brown, 2007 [30]	2	Case definition too broad
29	Godin, 2005 [71]	4	Case prevalence above 10%
30	Godin, 2009 [56]	5	Outcome sickness absence
31	Greenglass, 2001 [4]	1	No case definition
32	Griffin, 2002 [70]	4	Case prevalence above 10%
33	Hanson, 2015 [48]	3	Case definition not validated against an acceptable standard
34	Ibrahim, 2009 [65]	5	Risk estimates without confidence limits
35	Idris, 2014 [5]	1	No case definition
36	Kato, 2014 [72]	4	Case prevalence above 10%
37	Kim, 2013 [51]	3	Case definition not validated against an acceptable standard
38	Kim, 2017 [73]	4	Case prevalence above 10%
39	Kim, 2018 [74]	4	Case prevalence above 10%
40	Kivimaki, 2007 [31]	2	Case definition too broad
41	Lamy, 2013 [6]	1	No case definition
42	Lee, 2015 [54]	5	No relevant exposure
43	Lee, 2016 [7]	1	No case definition
44	Lunau, 2013 [90]	7	Selective reporting
45	Lunau, 2018 [85]	6	Study specifically aimed to examine effect modification
46	Madsen, 2014 [84]	6	Study specifically aimed to examine effect modification
47	Magnusson Hanson, 2014 [8]	1	No case definition
48	Magnusson Hanson, 2016 [82]	6	Study specifically aimed to examine effect mediation
49	Magnusson Hanson, 2017 [83]	6	Study specifically aimed to examine effect mediation
50	Mausner-Dorsch, 2000 [60]	5	Cross-sectional, exposure not shift work or work hours
51	Melchior, 2007 [61]	5	Cross-sectional, exposure not shift work or work hours
52	Meneton, 2017 [75]	4	Case prevalence above 10%
53	Mezuk, 2011 [62]	5	Cross-sectional, exposure not shift work or work hours
54	Moore, 2004 [9]	1	No case definition
55	Muntaner, 2006 [10]	1	No case definition
56	Nakao, 2006 [63]	5	Cross-sectional, exposure not shift work or work hours
57	Nakata, 2011 [76]	4	Case prevalence above 10%
58	Netterstrom, 2010 [77]	4	Case prevalence above 10%
59	Niedhammer, 1998 [78]	4	Case prevalence above 10%
60	Nyberg, 2017 [11]	1	No case definition
61	Nyberg, 2018 [32]	2	Case definition too broad
62	Oksanen, 2010 [91]	8	Overlap with Kouvonen et al. 2008 [92]
63	Paterniti, 2002 [12]	1	No case definition

64	Peter, 2016 [13]	1	No case definition
65	Sen, 2010 [55]	5	No relevant exposure
66	Shirangi, 2013 [14]	1	No case definition
67	Siegrist, 2012 [88]	7	Selective reporting
68	Sinokki, 2009 [93]	8	Overlap with Sinokki et al. 2009 [93]
69	Stansfeld, 2003 [33]	2	Case definition too broad
70	Stansfeld, 2008 [64]	5	Cross-sectional, exposure not shift work or work hours
71	Stoetzer, 2009 [99]	8	Overlap with FandinoLosada et al. 2013 [100]
72	Takahashi, 2012 [15]	1	No case definition
73	Taris, 1999 [16]	1	No case definition
74	Theorell, 2014 [17]	1	No case definition
75	Tomioka, 2011 [79]	4	Case prevalence above 10%
76	Tornroos, 2015 [18]	1	No case definition
77	Uchida, 2018 [19]	1	No case definition
78	Virtanen, 2008 [35]	2	Case definition too broad
79	Virtanen, 2011 [95]	8	Overlap with Virtanen et al. 2012 [96]
80	Virtanen, 2015 [80]	4	Case prevalence above 10%
81	Waldenstrom, 2008 [34]	2	Case definition too broad
82	Wang, 2011 [97]	8	Overlap with Shields et al. 2006 [98]
83	Weigl, 2012 [52]	3	Case definition not validated against an acceptable standard
84	Woodward, 1999 [20]	1	No case definition
85	Waage, 2014 [21]	1	No case definition
86	Özdemir, 2013 [22]	1	No case definition

Appendix 3. Description of studies – tables and forest plots

Table A3.1 Summary characteristics of included studies page 16
Table A3.2 Characteristics of 54 individual studies (51 original papers) included in review page 18
Table A3.3 Control for potential confounders by study page 26
Forest plots, figures A3.1 to A3.11 and textbox with column and abbreviation explanations page 30

**Table A3.1 Summary characteristics of included studies**	
**Source population**	N
Employed general population	30
Public employees	10
Hospital employees	5
Union members	4
Private company employees	3
Elder care workers	1
Mixed population	1
**Study population, Country**	N
Denmark	20
Finland	9
Canada	8
USA	3
Germany, Norway, Sweden, United Kingdom, each two studies	8
France, Italy, Japan, Korea, Netherlands, each one study	5
Several countries	1
**Publication year**	N
1999-2009	19
2010-2014	21
2015-2018	14
**Follow-up years** (cohort studies)	N
1 - <2 years	5
2 years	13
>2 - 4 years	16
>4 years	10
Varying, 11 and 15 years	3

**Table A3.1** (continued)	
**Follow-up times** (cohort studies)	N
1 time	30
2-3 times	5
4-6 times	2
Registers, continuous (e.g. antidepressants, hospital admissions	10
**Participants, cohort studies**	N
1365 - <5000	30
5000 - < 10000	11
10000 – 27461	6
**Participants, cross-sectional studies**	
1385 - <5000	1
5000 - < 10000	2
10000 – 11450	2
**Participants, nested case-referent studies**	
Cases: 14166 / referents: 58060 in each of the two studies (same material)	2
**Participation (%)**	N
≥80%	19
≥60-80%	27
≥40-60%	2
<40	1
No information	5
**Sex (percent male)**	N
0-39	19
40-59	28
60-78	6
No information	1
**Age distribution**	N
Too heterogeneously reported to be summarized (employed adults, 18-75 years old)	

**Table A3.1** (continued) **Baseline prevalence of outcome (%)**				
**Prevalence period**	N	Median	Minimum	Maximum
0 to 9 months	18	2.7	1.3	8.7
12 months	8	3.9	2.5	9.1
1 to 6 years	9	4.9	2.3	10.5
Many years or lifetime	11	8.0	2.6	18.0
Varying by country (0 to lifetime)	1	0.6	-	-
Not reported (5) or not applicable (2)	7	-	-	-

Table A3.2 Characteristics of 54 individual studies (51 original papers) included in review (nr: not reported)

First author, Year Substudy, study (label) or acronym, Country	Population	Design	Follow-up years/times	Baseline Participants N	% Male	Age range, mean (SD) or other	Follow-up participants: percentage of baseline participants	Exposures	Exposure source	Measure of depression
**Bonde 2009 [113] County Denmark**	Public employees	Cohort	4/1	13335	21	16-71	≥60-80	Demands Control Job-strain Support	Work-unit average	AD treatment
**Bonde 2009 [113] Municipality Denmark**	Public employees	Cohort	4/1	4815	23	17-67	≥60-80	Decision authority Skill-discretion Work-load	Work-unit average	AD treatment
**Bonde 2016 [103] PRISME/WBH Denmark**	Public employees	Cohort	4/2	7502	26	>45: 42%	≥60-80	Bullying	Self-report, perceived	SCAN and MDI
**Dembe 2016 [114] NLSY79 USA**	Employed population	Cross-sectional	-	7492	51	46-51	Nr	Work hours	Self-report	Doctor's diagnosis, self-report
**Dement 2014 [115] DHSSS USA**	Hospital employees	Cohort	6/1	9884	21	<30-60+	≥80	Violence, threats	Notified claims Registry records	AD treatment
**d'Errico 2011 [116] CGIL Italy**	Trade union members	Cohort	6/1	2046	51	15-45+	≥40-60	Demands Control Job-strain Work hours Shift work	Self-report, perceived	AD treatment
**Fandino-Losada 2013 [100] PART Sweden**	Employed population	Cohort	3/1	4427	45	20-64	≥80	Demands Control Job-strain	Self-report, perceived	MDI-algorithm

Table A3.2 (continued) **Characteristics of 54 individual studies (51 original papers) included in review** (nr: not reported)

First author, Year Substudy, study (label) or acronym, Country	Population	Design	Follow-up years/times	Baseline Participants N	% Male	Age range, mean (SD) or other	Follow-up participants: percentage of baseline participants	Exposures	Exposure source	Measure of depression
**Grynderup 2012 [117] PRISME[109] Denmark**	Public employees	Cohort	2/1	3046	21	<35-55+	≥60-80	Demands Control Job-strain	Work-unit average	SCAN
**Grynderup 2013 [118] PRISME Denmark**	Public employees	Cohort	2/1	3047	21	<35-55+	≥60-80	Procedural and relational justice	Work-unit average	SCAN
**Gullander 2014 [101] PRISME/WBH Denmark**	Public employees	Cohort	4/2	5198	25	<35-55+	≥60-80	Bullying	Work-unit average	SCAN and MDI
**Hall 2018 [119] NSWHN Canada**	Nurses	Cross-sectional	-	11450	5	<35-55+	≥80	Night work, Shift work	Self-report	CIDI-SFMD
**Hannerz 2016 [120] (COPSOQ/DANES/ DWECS) Denmark**	Employed population	Cohort	2-5/1	19259	55	21-59	≥60-80	Work hours	Self-report	AD treatment
**Hanson 2013 [111] SHLOSH Sweden**	Employed population	Cohort	1/1	3661	49	20-59	≥60-80	Demands Decision authority Skill discretion Emotional demands	Self-report, perceived	AD treatment
**Hanson 2013 [111] DWECS Denmark**	Employed population	Cohort	1/1	6418	50	20-59	≥60-80	Demands Decision authority Skill discretion Emotional demands	Self-report, perceived	AD treatment

Table A3.2 (continued) **Characteristics of 54 individual studies (51 original papers) included in review** (nr: not reported)

First author, Year Substudy, study (label) or acronym, Country	Population	Design	Follow-up years/times	Baseline Participants N	% Male	Age range, mean (SD) or other	Follow-up participants: percentage of baseline participants	Exposures	Exposure source	Measure of depression
**Hogh 2016 [102]<br>WBH<br>Denmark**	Company employees	Cohort	2/2	1455	30	47 (9.4)	≥40-60	Bullying (negative behaviour, Direct harassment, isolation, intimidation)	Self-report, perceived	SCAN and MDI
**Joensuu 2010 [38]<br>Still Working Study<br>Finland**	Employed forest industry	Cohort	15/cont.	13868	77	<35-65	≥60-80	Decision authority<br>Skill discretion<br>Support, colleagues<br>Support, supervisors	Self-report, perceived	Doctor's diagnosis, Hospital records[a]
**Kim 2016 [121]<br>KOWEPS<br>Korea**	Employed population	Cohort	4/3	2733	64	20-59	?	Work hours	Self-report	CES-D 11 (≥16)
**Kivimaki 2003 [122]<br>Hospital personnel study<br>Finland**	Hospital employees	Cohort	2/1	5432	11	18-63	≥60-80	Bullying	Self-report, perceived	Doctor's diagnosis, self-report
**Kivimaki 2007 [123]<br>Ten town study<br>Finland**	Employed population	Cohort	2-4/1	15995	11	19-62	≥60-80	Effort-reward-imbalance<br>Procedural and relational justice	Self-report, perceived	Doctor's diagnosis, self-report
**Kivimaki 2007 [123]<br>Hospital personnel study<br>Finland**	Hospital employees	Cohort	2-4/1	4445	22	20-60	≥60-80	Effort-reward-imbalance<br>Procedural and relational justice	Self-report, perceived	Doctor's diagnosis, self-report
**Kleppa 2008 [124]*<br>HUSK<br>Norway**	Employed population	Cross-sectional	-	10442	59	40-45	≥60-80	Work hours<br>Night work<br>Shift work	Self-report	HADS-D (≥8)

Table A3.2 (continued) **Characteristics of 54 individual studies (51 original papers) included in review** (nr: not reported)

First author, Year Substudy, study (label) or acronym, Country	Population	Design	Follow-up years/times	Baseline Participants N	% Male	Age range, mean (SD) or other	Follow-up participants: percentage of baseline participants	Exposures	Exposure source	Measure of depression
**Laaksonen 2012 [125] (City of Helsinki study) Finland**	Public employees	Cohort	5/1	5786	78	40-60	≥60-80	Demands Control, Support, Work hours Shift work	Self-report, perceived	AD treatment
**Madsen 2010 [110] DWECS Denmark**	Employed population	Cohort	5/1	4958	52	40 (nr)	≥60-80	Violence, threats	Self-report, perceived	AD treatment
**Madsen 2017 [126] IPD-Work Several countries**	Mixed empl. Populations	Cohort	Varying/cont.	27461	41	43 (9.6)	≥80	Job-strain	Self-report, perceived	Doctor's diagnosis, Hospital records
**Niedhammer 2015 [127] SIP France**	Employed population	Cohort	4/1	4717	51	20-74	≥60-80	Demands Control Support Reward Emotional demands Job-insecurity Work hours Night work Shift work	Self-report, perceived Self-report	MINI
**Nielsen 2016 [128] DWECS-2000 Denmark**	Employed population	Cohort	5/cont.	4541	52	40 (10)	≥80	Effort-reward-imbalance	Self-report, perceived	AD treatment
**Nigatu 2018 [109] (Alberta cohort study) Canada**	Employed population	Cohort	4/3	4200	45	45 (10)	≥60-80	Job-strain Effort-reward-imbalance	Self-report, perceived	CIDI-WMH-Auto 2.1

**Table A3.2** (continued) **Characteristics of 54 individual studies (51 original papers) included in review** (nr: not reported)										
**First author, Year Substudy, study (label) or acronym, Country**	**Population**	**Design**	**Follow-up years/times**	**Baseline Participants N**	**% Male**	**Age range, mean (SD) or other**	**Follow-up participants: percentage of baseline participants**	**Exposures**	**Exposure source**	**Measure of depression**
**Oyane 2013 [129] SUSSH Norway**	Nurses	Cross-sectional	-	2035	9	21-63	<60	Night work Shift work	Self-report	HADS-D (≥8)
**Plaisier 2007 [39] NEMESIS Netherlands**	Employed population	Cohort	2/1	3048	58	18-65	≥80	Demands Control Job-insecurity	Self-report, perceived	CIDI[b]
**Rugulies 2006 [130] DWECS Denmark**	Employed population	Cohort	5/1	4133	52	<35-55+	≥80	Demands Decision authority Skill discretion Support Job-insecurity	Self-report, perceived	MHI-5 (≤52)
**Rugulies 2010 [131] (Danish Longitudinal Study on Work, Unemployment and Health) Denmark**	Employed population	Cohort	3.5/cont.	5142	47	37-56	≥80	Job-insecurity	Self-report, perceived	AD treatment
**Rugulies 2012 [132] (Eldercare sector study) Denmark**	Eldercare workers	Cohort	1.7/1	4435	0	46 (9)	≥60-80	Bullying	Self-report, perceived	MDI-algorithm
**Rugulies 2013 [87] DWECS Denmark**	Employed population	Cohort	5/1	2701	49	40 (9)	≥60-80	Effort-reward-imbalance	Self-report, perceived	MHI-5 (≤52)
**Shields 1999 [104] CNPHS Canada**	Employed population	Cohort	2/1	3830	57	25-54	≥80	Work hours Shift work	Self-report	CIDI-SFMD

Table A3.2 (continued) **Characteristics of 54 individual studies (51 original papers) included in review** (nr: not reported)

First author, Year Substudy, study (label) or acronym, Country	Population	Design	Follow-up years/times	Baseline Participants N	% Male	Age range, mean (SD) or other	Follow-up participants: percentage of baseline participants	Exposures	Exposure source	Measure of depression
**Shields 2006 [98]**<br>**CNPHS**<br>**Canada**	Employed population	Cohort	2/1	12011	51	18-75	≥80	Job-strain<br>Support, colleagues<br>Support, supervisors	Self-report, perceived	CIDI-SFMD
**Sinokki 2009 [94]**<br>**Health 2000 study**<br>**Finland**	Employed population	Cohort	3/cont.	3429	49	30-64	≥80	Support, colleagues<br>Support, supervisors	Self-report, perceived	AD treatment
**Smith 12 [107]**<br>**CNPHS**<br>**Canada**	Employed population	Cohort	2/1	3735	54	26-60	Nr	Demands<br>Control<br>Job-strain<br>Support	Self-report, perceived	CIDI-SFMD
**Stansfeld 2012 [112]**<br>**Whitehall II**<br>**United Kingdom**	Public employees	Cohort	2/1	3942	74	35-55	≥60-80	Job-strain<br>Support	Self-report, perceived	UM-CIDI
**Thielen 2011 [133]**<br>**(Danish Longitudinal Study on Work, Unemployment and Health)**<br>**Denmark**	Employed population	Cohort	3.5/cont.	4661	52	40, 50	≥80	Demands<br>Skill discretion (low work variance)<br>Support, colleagues<br>Support, supervisors<br>Emotional demands	Self-report, perceived	AD treatment
**Tokuyam 2003 [134]**<br>**(Insurance company study)**<br>**Japan**	Insurance company	Cohort	4/4	1365	47	20-73	≥60-80	Work hours<br>Work-load	Self-report<br>Self-report, perceived	Zung SDS (>40)

Table A3.2 (continued) **Characteristics of 54 individual studies (51 original papers) included in review** (nr: not reported)

First author, Year Substudy, study (label) or acronym, Country	Population	Design	Follow-up years/times	Baseline Participants N	% Male	Age range, mean (SD) or other	Follow-up participants: percentage of baseline participants	Exposures	Exposure source	Measure of depression
**Vammen 2016 [135] PRISME Denmark**	Public employees	Cohort	2/1	3125	22	<35->55	≥60-80	Emotional demands	Self-report, perceived Self-report, content Work-unit average	SCAN
**Varma 2012 [136] (Senior Consultant Study) Denmark**	Senior Medical Consultants	Cohort	1.6/cont.	2790	71	55 (6.6)	≥80	Work hours	Self-report	AD treatment
**Virtanen 2007 [137] Health 2000 study Finland**	Employed population	Cohort	3/cont	3366	49	M:44.2 (8.4), F:45.0 (8.2)	≥80	Demands Control Job-strain	Self-report, perceived	AD treatment
**Virtanen 2008 [138] Hospital personnel study Finland**	Hospital employees	Cohort	5/cont	7340	8	39 (10)	≥80	Work-load (hospital bed occupancy higher than the norm)	Objective measurements, register information	AD treatment
**Virtanen 2012 [96] Whitehall II United Kingdom**	Public employees	Cohort	3.8-7.2/1	2123	77	41-61	≥60-80	Job-strain Social support Work hours	Self-report, perceived	UM-CIDI
**Wang 2004 [105] CNPHS Canada**	Employed population	Cohort	6/4	7371	nr	18+	Nr	Demands Decision authority Skill discretion Job-insecurity	Self-report, perceived	CIDI-SFMD
**Wang 2009 [106] CNPHS Canada**	Employed population	Cohort	11/6	4866	54	36 (nr)	≥80	Job-strain	Self-report, perceived	CIDI-SFMD

Table A3.2 (continued) **Characteristics of 54 individual studies (51 original papers) included in review** (nr: not reported)

First author, Year Substudy, study (label) or acronym, Country	Population	Design	Follow-up years/times	Baseline Participants N	% Male	Age range, mean (SD) or other	Follow-up participants: percentage of baseline participants	Exposures	Exposure source	Measure of depression
**Wang 2012 [108] (Alberta cohort study) Canada**	Employed population	Cohort	1/1	2752	56	25-65	≥60-80	Support, colleagues Support, supervisors Job-insecurity	Self-report, perceived	CIDI-WMH-Auto 2.1
**Wege 2017 [139] GSOEP Germany**	Employed population	Cohort	2/1	7073	53	44 (11)	≥60-80	Job-insecurity	Self-report, perceived	Doctor's diagnosis, self-report
**Wege 2018 [86] GSOEP Germany**	Employed population	Cohort	271	6693	51	44 (11)	≥60-80	Effort-reward-imbalance	Self-report, perceived	Doctor's diagnosis, self-report
**Wieclaw 2006 [41] (Danish Empl. Study) Denmark**	Employed population	Nested case-referent	-	14166 cases/ 58060 referents	38	18-65	≥80	Violence, threats of violence	Job-exposure-matrix	Doctor's diagnosis, Hospital records[c]
**Wieclaw 2008 [40] (Danish Empl. Study) Denmark**	Employed population	Nested case-referent	-	14166 cases/ 58060 referents	38	18-65	≥80	Demands Control Job-strain Emotional demands	Job-exposure-matrix	Doctor's diagnosis, Hospital records[c]
**Wirth 2017 [140] NHANES USA**	Employed population	Cross-sectional	-	7434	49	47 (nr)	Na	Shift work	Self-report	PHQ-9 (≥10)
**Ylipaavalniemi 2005 [141] Hospital personnel study Finland**	Hospital employees	Cohort	2/1	4815	11	44 (nr)	≥80	Demands Control Job-strain Procedural and relational justice	Self-report, perceived	Doctor's diagnosis, self-report

[a] Includes all affective disorders with unipolar depression; [b] Includes dysthymia; [c] Includes all affective disorders

Table A3.3 Control for potential confounders by study. (0=no, 1=yes; na: not applicable for non-cohort studies)

First author, publication year (substudy)	Previous depression[d]	Depression at baseline[d,f]	Depressive symptoms at baseline[e,f]	Family history of depression	Neuroti-cism/ Persona-lity	Living alone/ Marital status	Recent life-events	Childhood adversity	Socioeconomic status [g]	Somatic illness/ self-rated health	Number of confoun-ders controlled for
Bonde 2009 (County) [113]	0	0	0	0	0	1	0	0	0	0	1
Bonde 2009 (Municipality) [113]	0	0	0	0	0	1	0	0	0	0	1
Bonde 2016[103]	0	1	0	0	0	0	0	0	0	0	1
Dembe 2016 [114][a]	0	na	na	0	0	0	0	0	0	0	0
Dement 2014 [115]	0	0	0	0	0	0	0	0	0	0	0
d'Errico 2011 [116]	0	0	0	0	0	0	0	0	0	0	0
Fandino-Losada 2013 [100]	0	1	1	0	0	1	1	0	1	0	5
Grynderup 2012 [117]	0	1	0	1	1	1	1	0	1	0	6
Grynderup 2013 [118]	0	1	1	1	1	1	1	0	1	0	7
Gullander 2014 [101]	0	1	0	1	1	1	1	0	0	0	5
Hall 2018 [119][a]	0	na	na	0	0	1	0	0	0	1	2
Hannerz 2016 [120]	0	0	0	0	0	0	0	0	1	0	1
Hanson 2013 (DWECS) [111]	0	1	1	0	0	1	0	0	1	1	5
Hanson 2013 (SLOSH) [111]	0	1	1	0	0	1	0	0	1	1	5
Hoegh 2016 [102]	0	1	1	0	1	1	0	0	1	0	5
Joensuu 2010 [38]	0	0	0	0	0	0	0	0	0	1	1
Kim 2016 [121]	0	1	0	0	0	1	0	0	1	1	4
Kivimaki 2003 [122]	0	0	0	0	0	0	0	0	0	1	1
Kivimaki 2007 (10 town) [123]	0	0	0	0	0	0	0	0	0	0	0

**Table A3.3** (continued) **Control for potential confounders by study.** (0=no, 1=yes; na: not applicable for non-cohort studies)											
**First author, publication year (substudy)**	**Previous depression[d]**	**Depression at baseline[d,f]**	**Depressive symptoms at baseline[e,f]**	**Family history of depression**	**Neuroti-cism/ Persona-lity**	**Living alone/ Marital status**	**Recent life-events**	**Childhood adversity**	**Socioeconomic status [g]**	**Somatic illness/ self-rated health**	**Number of confoun-ders controlled for**
Kivimaki 2007 (Hospital personnel) [123]	0	0	0	0	0	0	0	0	0	0	0
Kleppa 2008 [124][a]	0	na	na	0	0	0	0	0	0	0	0
Laaksonen 2012 [125]	0	0	0	0	0	0	0	0	0	0	0
Madsen 2010 [110]	0	1	0	0	0	1	0	0	1	0	3
Madsen 2017 [126]	0	0	0	0	0	1	0	0	0	0	1
Niedhammer 2015 [127]	0	1	0	0	0	1	1	1	1	0	5
Nielsen 2016 [128]	0	1	1	0	0	1	0	0	1	1	5
Nigatu 2018 [109]	1	1	0	0	0	1	0	0	1	0	4
Oyane 2013 [129][a]	0	na	na	0	0	1	0	0	1	0	2
Plaisier 2007 [39]	0	1	0	0	0	0	0	0	0	1	2
Rugulies 2006 [130]	0	1	1	0	0	1	0	0	1	0	4
Rugulies 2010 [131]	0	1	1	0	0	1	0	0	0	0	3
Rugulies 2012 [132]	0	1	0	0	0	1	0	0	0	0	2
Rugulies 2013 [87]	0	1	1	0	0	1	0	0	1	1	5
Shields 1999 [104]	0	1	0	0	0	1	0	0	1	0	3
Shields 2006 [98]	0	1	0	0	0	1	0	0	1	1	4
Sinokki 2009 [94]	0	1	0	0	0	1	0	0	1	0	3
Smith 2012 [107]	0	1	0	1	0	1	0	0	1	1	5
Stansfeld 2012 [112]	0	0	1[h]	0	0	1	0	0	1	1	4

**Table A3.3** (continued) **Control for potential confounders by study.** (0=no, 1=yes; na: not applicable for non-cohort studies)											
**First author, publication year (substudy)**	**Previous depression[d]**	**Depression at baseline[d,f]**	**Depressive symptoms at baseline[e,f]**	**Family history of depression**	**Neuroti-cism/ Persona-lity**	**Living alone/ Marital status**	**Recent life-events**	**Childhood adversity**	**Socioeconomic status [g]**	**Somatic illness/ self-rated health**	**Number of confoun-ders controlled for**
Thielen 2011 [133]	0	1	1	0	0	1	0	0	0	1	4
Tokuyama 2003 [134][b]	1	1	0	0	1	0	1	1	0	1	6
Vammen 2016 [135][b]	0	1	0	1	1	1	1	0	1	1	7
Varma 2012 [136]	0	0	0	0	0	1	0	0	1	0	2
Virtanen 2007 [137]	0	1	0	0	0	1	0	0	1	0	3
Virtanen 2008 [138][c]	0 (na)	0 (na)	0 (na)	0 (na)	0 (na)	0 (na)	0 (na)	0 (na)	0 (na)	0 (na)	0 (na)
Virtanen 2012 [96]	0	0	0	0	0	1	0	0	1	1	3
Wang 2004 [105]	0	1	0	0	0	1	1	1	1	0	5
Wang 2009 [106]	1	1	0	0	0	1	1	1	1	1	7
Wang 2012 [108]	1	1	0	0	0	1	0	0	1	0	4
Wege 2017 [139]	0	0	0	0	0	1	0	0	1	1	3
Wege 2018 [86]	0	0	0	0	0	1	0	0	1	1	3
Wieclaw 2006 [41][a]	0	na	na	0	0	1	0	0	1	0	2
Wieclaw 2008 [40][a]	0	na	na	0	0	1	0	0	1	0	2
Wirth 2017 [140][a]	0	na	na	0	0	1	0	0	1	1	3
Ylipaavalniemi 2005 [141]	0	0	0	0	0	0	0	0	0	0	0
**Total**	**4**	**29**	**11**	**5**	**6**	**39**	**10**	**4**	**32**	**19**	na

[a] Cross-sectional or nested case-control studies;

[b] Some potential confounders excluded from most adjusted model after confounder selection procedure;

[c] Not relevant for this study because the potential confounders mentioned in the table are unlikely to be associated with the exposure in this study (objectively registered hospital bed occupancy higher than the norm);

[d] Sufficient control by interview or questionnaire instrument cases at baseline

[e] Sufficient control by continuous score

[f] Only relevant for cohort studies

[g] Sufficient if based on more than 3 categories

[h] GHQ-30 in some analyses

Forest Plots (Fig A3.1 to Fig A3.11)

(Textbox with column and abbrevation explanations at the end of this Appendix)

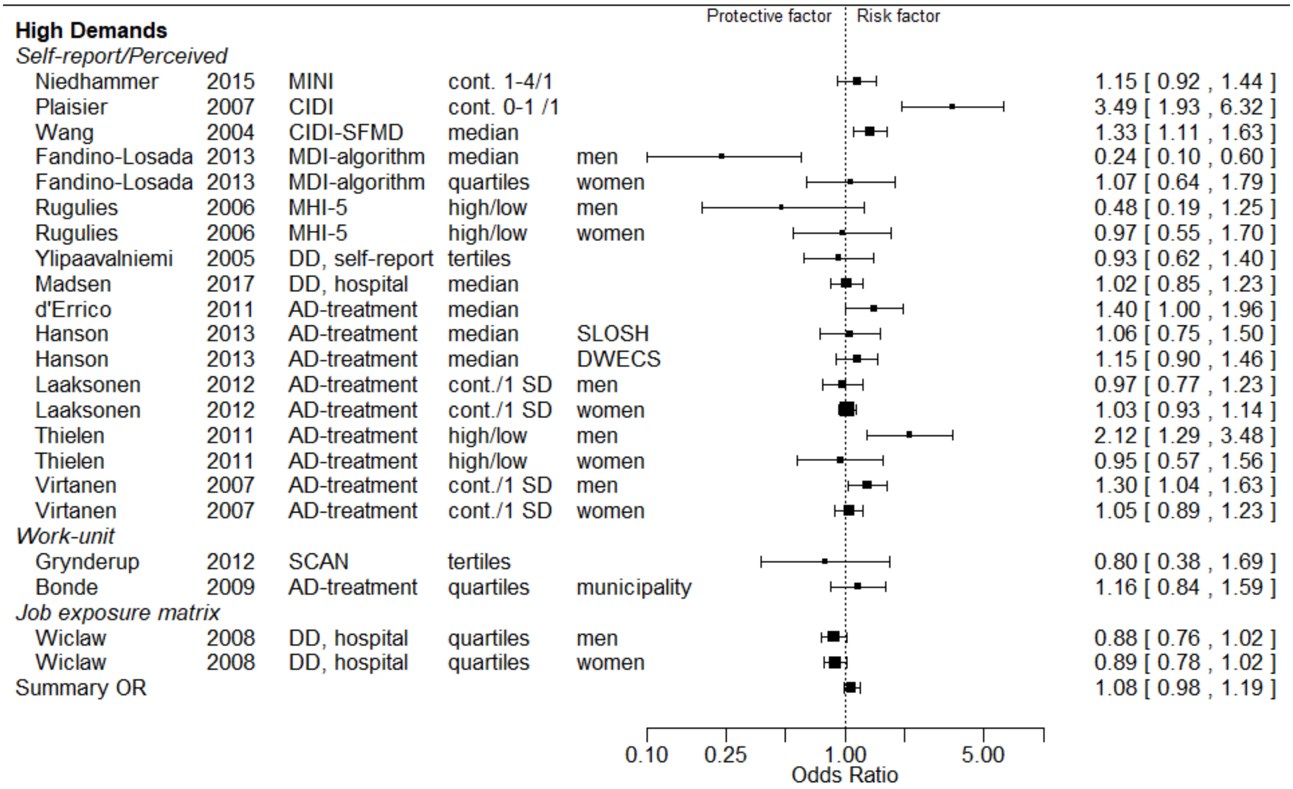

Fig A3.1 Forest plot for demands (abbreviations, see textbox after last forest plot)**.**

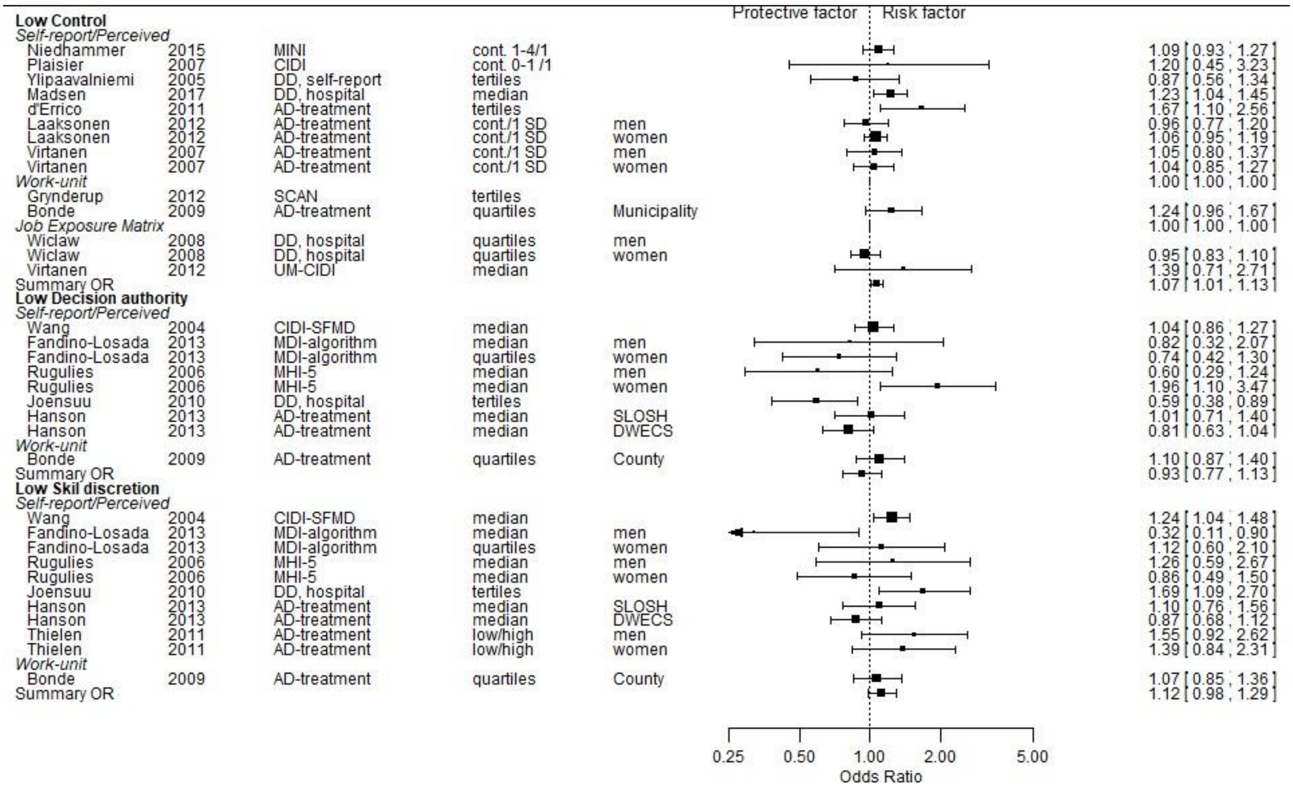

Fig A3.2 Forest plot for control, decision authority and skill discretion (abbreviations, see textbox after last forest plot)**.**

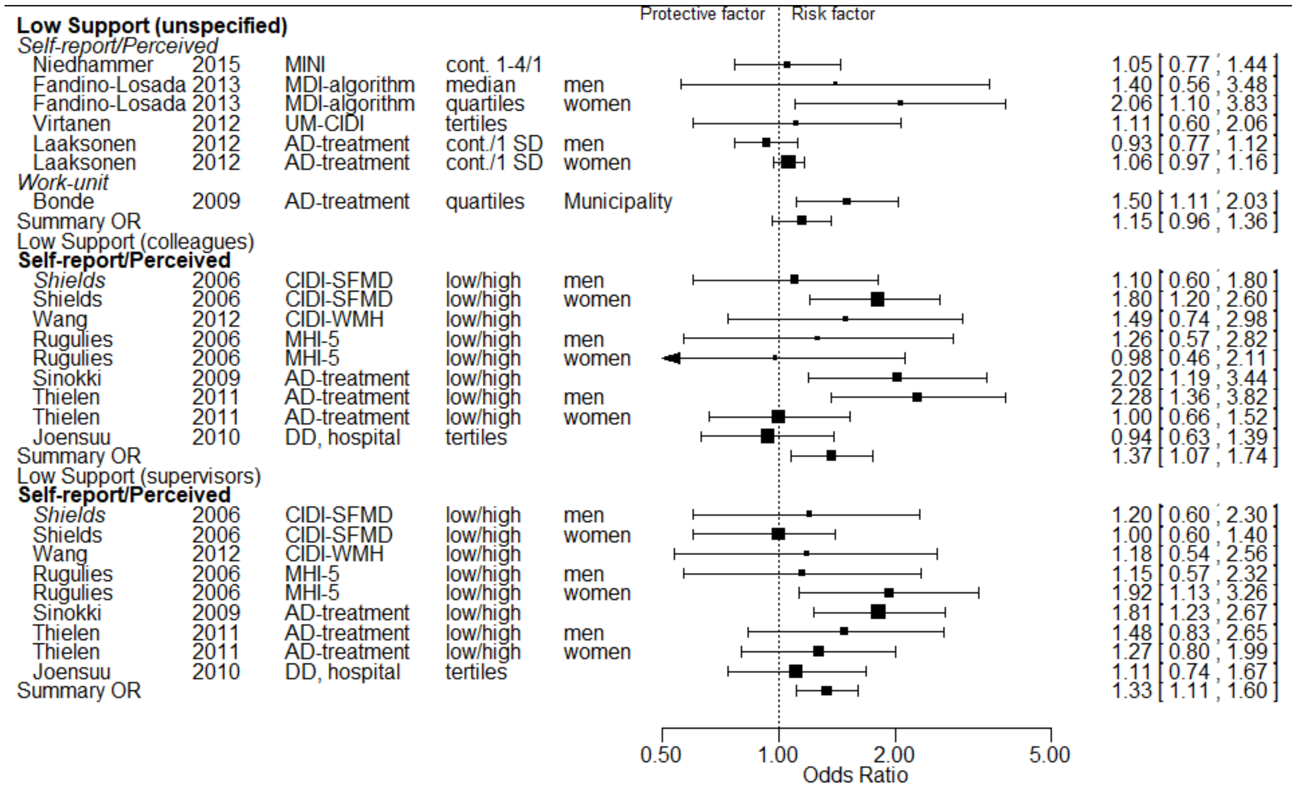

Fig A3.3 Forest plot for support (abbreviations, see textbox after last forest plot).

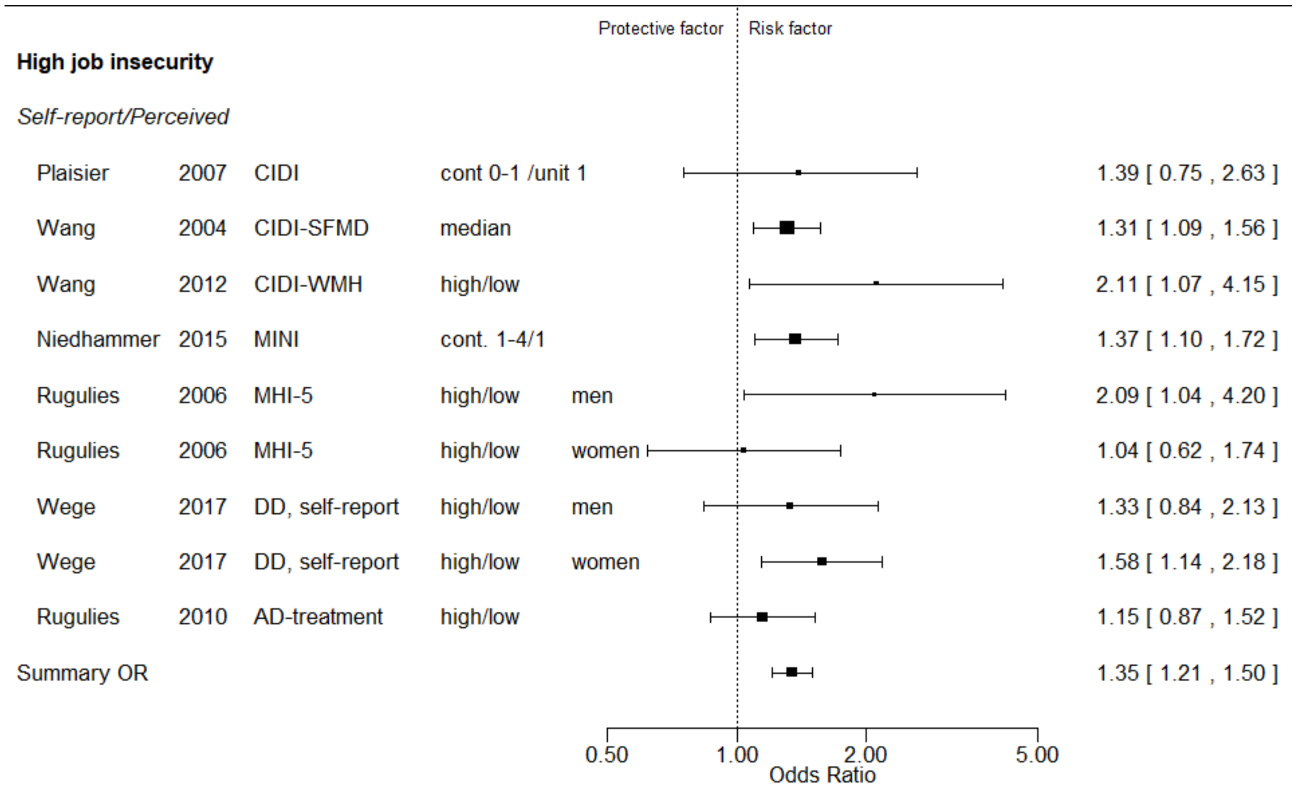

Fig A3.4 Forest plot for job-insecurity (abbreviations, see textbox after last forest plot)**.**

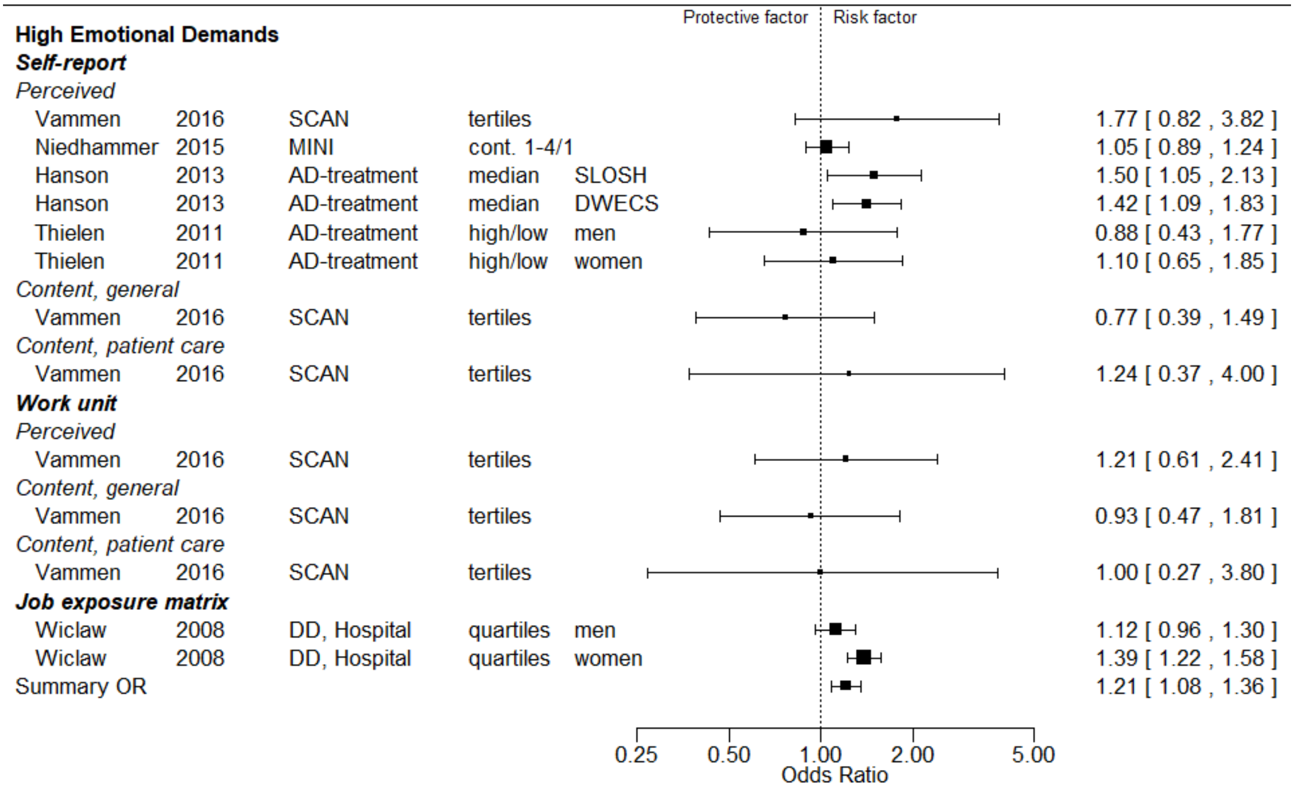

Fig A3.5 Forest plot for emotional demands (abbreviations, see textbox after last forest plot)**.**

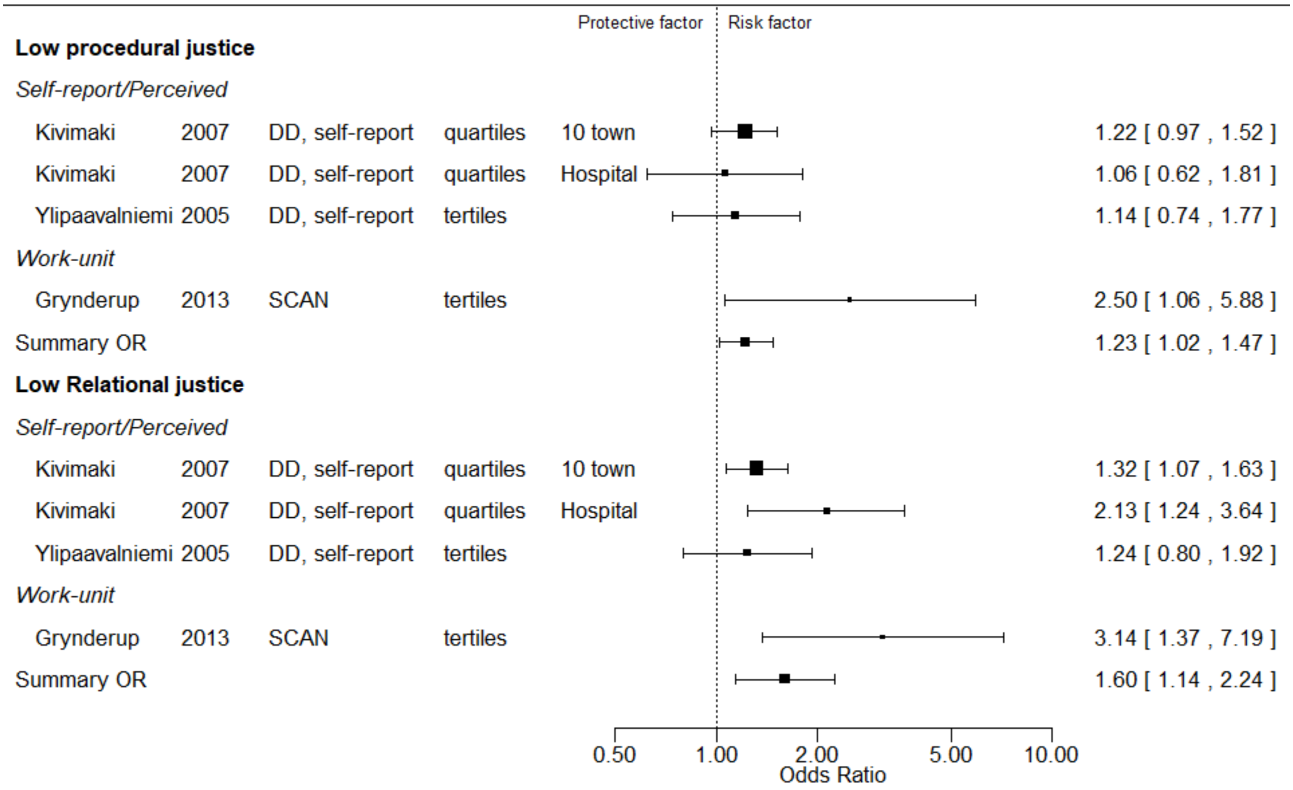

Fig A3.6 Forest plot for procedural and relational justice (abbreviations, see textbox after last forest plot)**.**

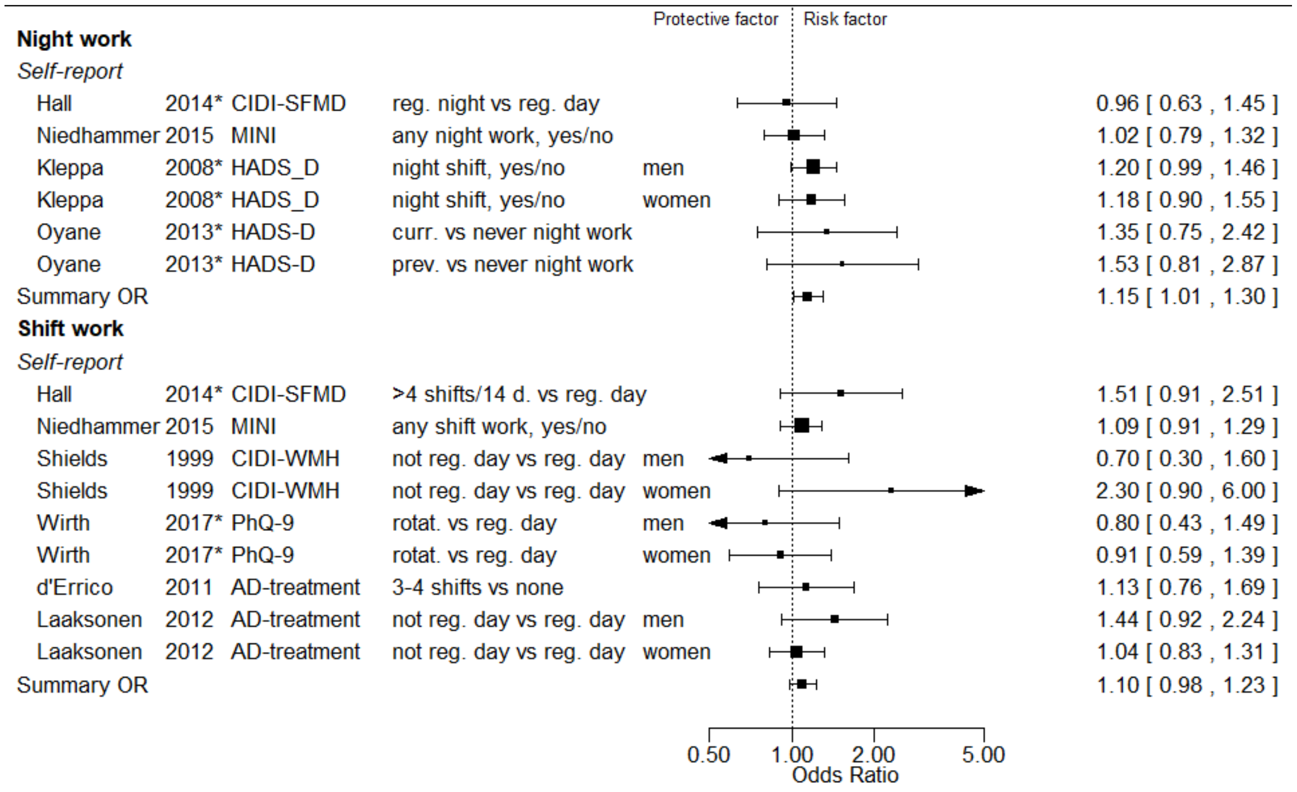

Fig A3.7 Forest plot for night work and shift work (abbreviations, see textbox after last forest plot)

'reg.' means regular, 'curr.' means current, 'd.' means day, 'rotat.' means rotating.

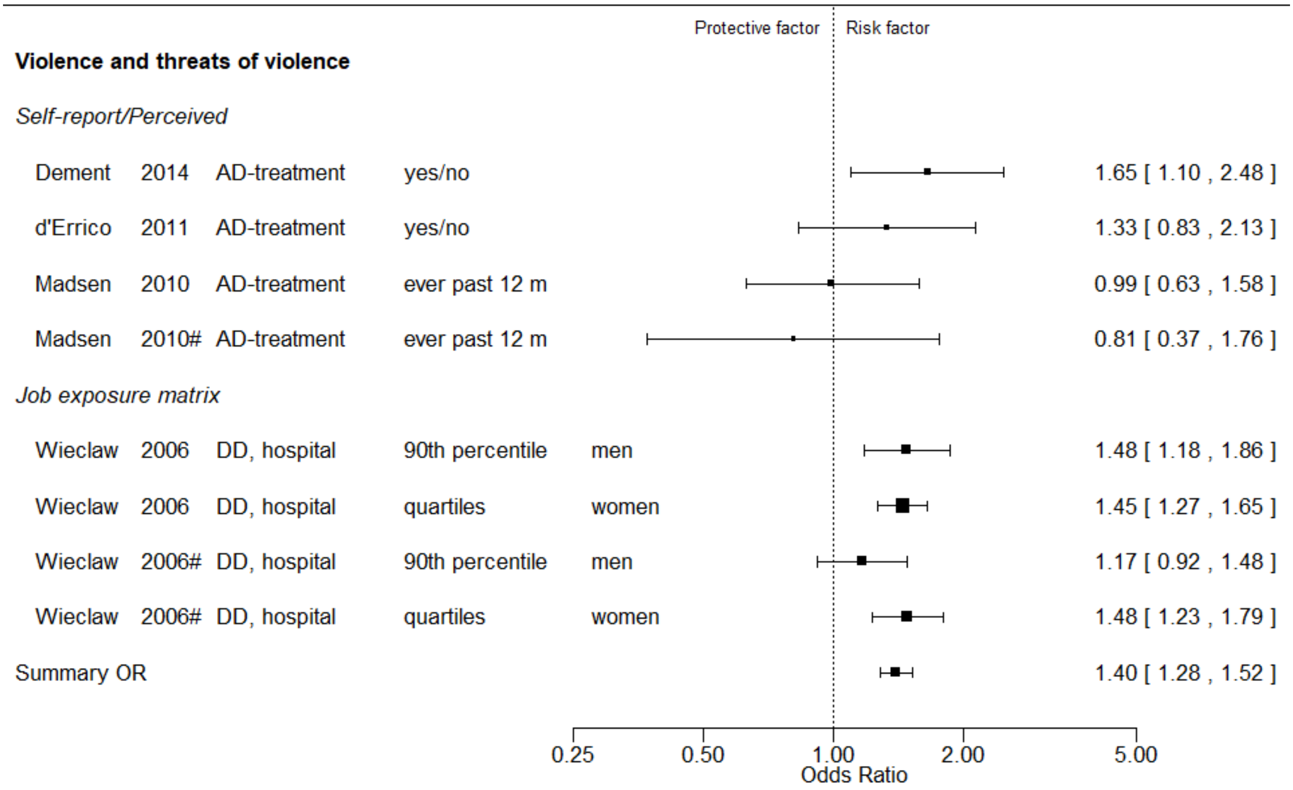

Fig A3.8 Forest plot for violence and threats of violence (abbreviations, see textbox after last forest plot)

means ‘threats of violence’

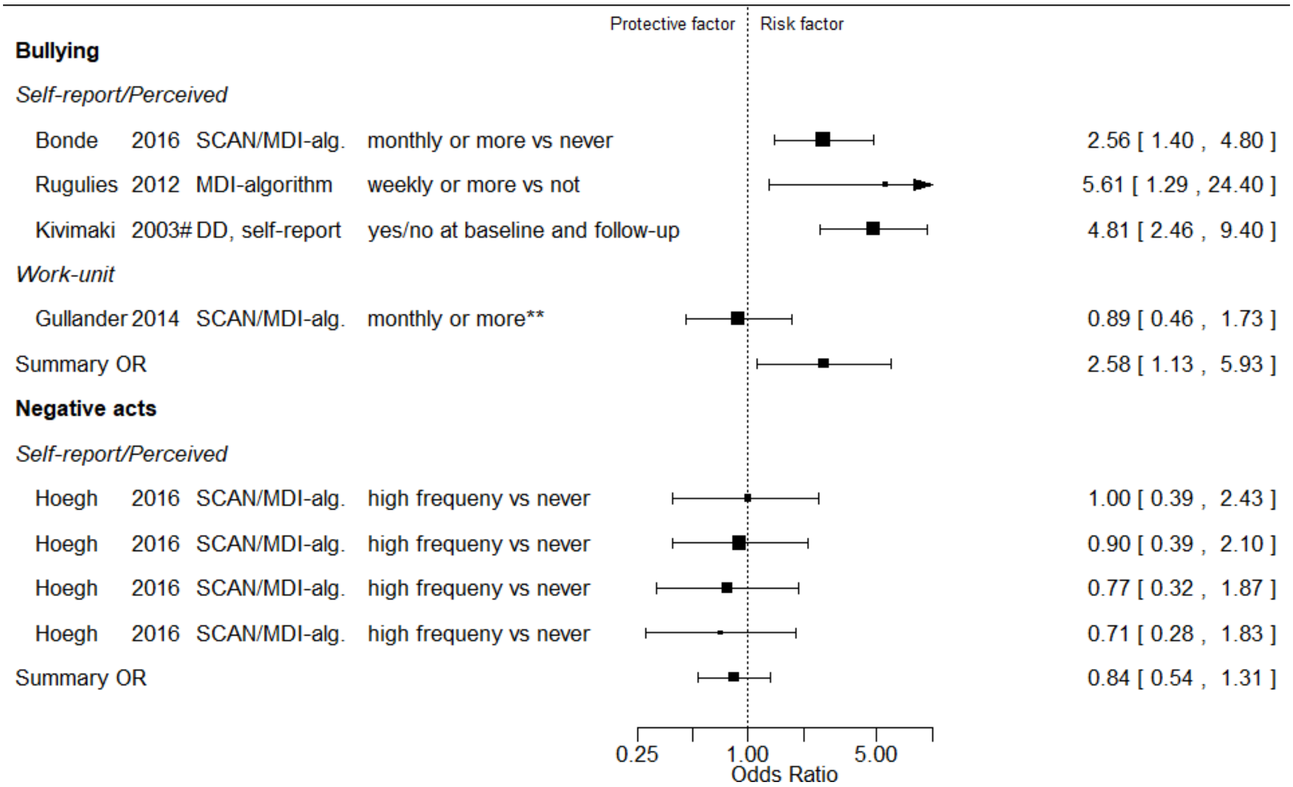

Fig A3.9 Forest plot for bullying and negative acts (abbreviations, see textbox after last forest plot)
risk estimate not truely prospective because it includes cross-sectional associations at follow-up,

**whitnessed by >30% vs 0% of work-unit members

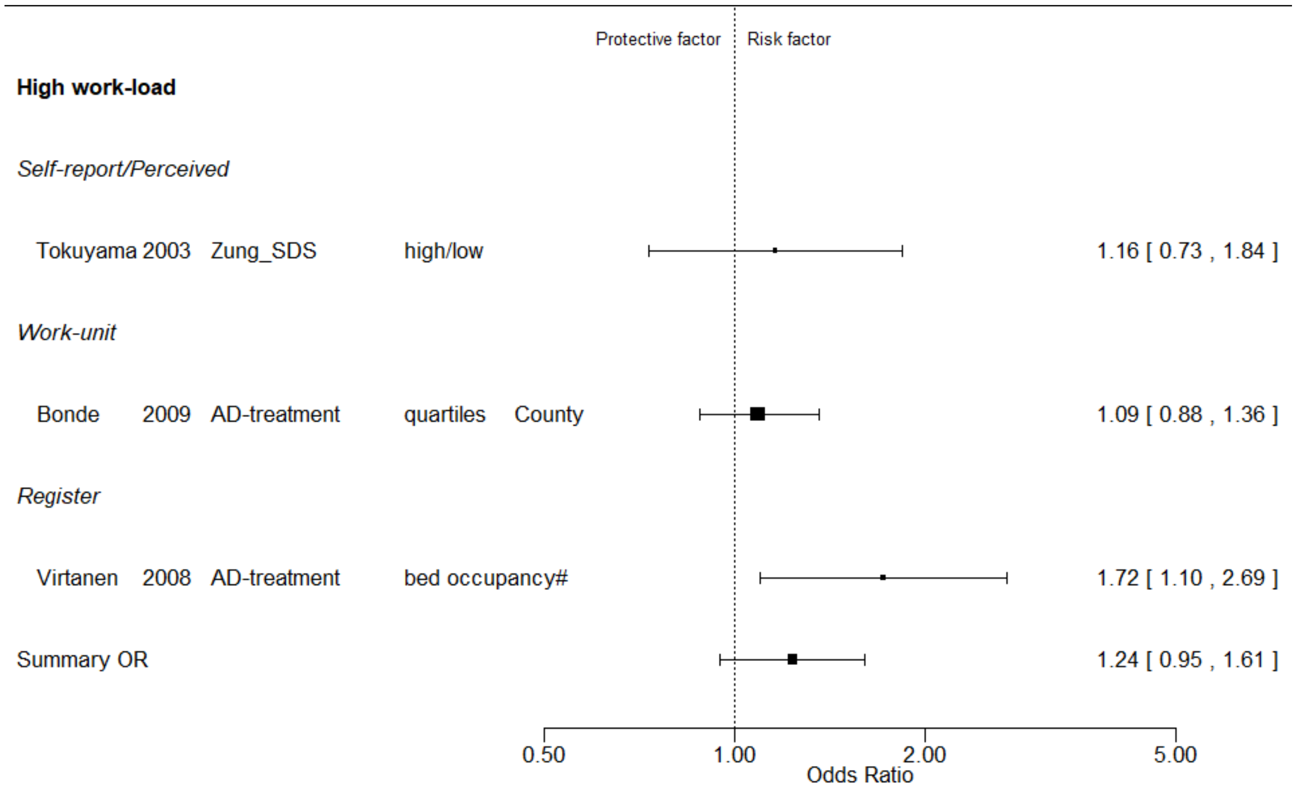

Fig A3.10 Forest plot for work-load (abbreviations, see textbox after last forest plot)**.**

>10% excess hospital bed occupancy compared to the norm during past 6 months vs none

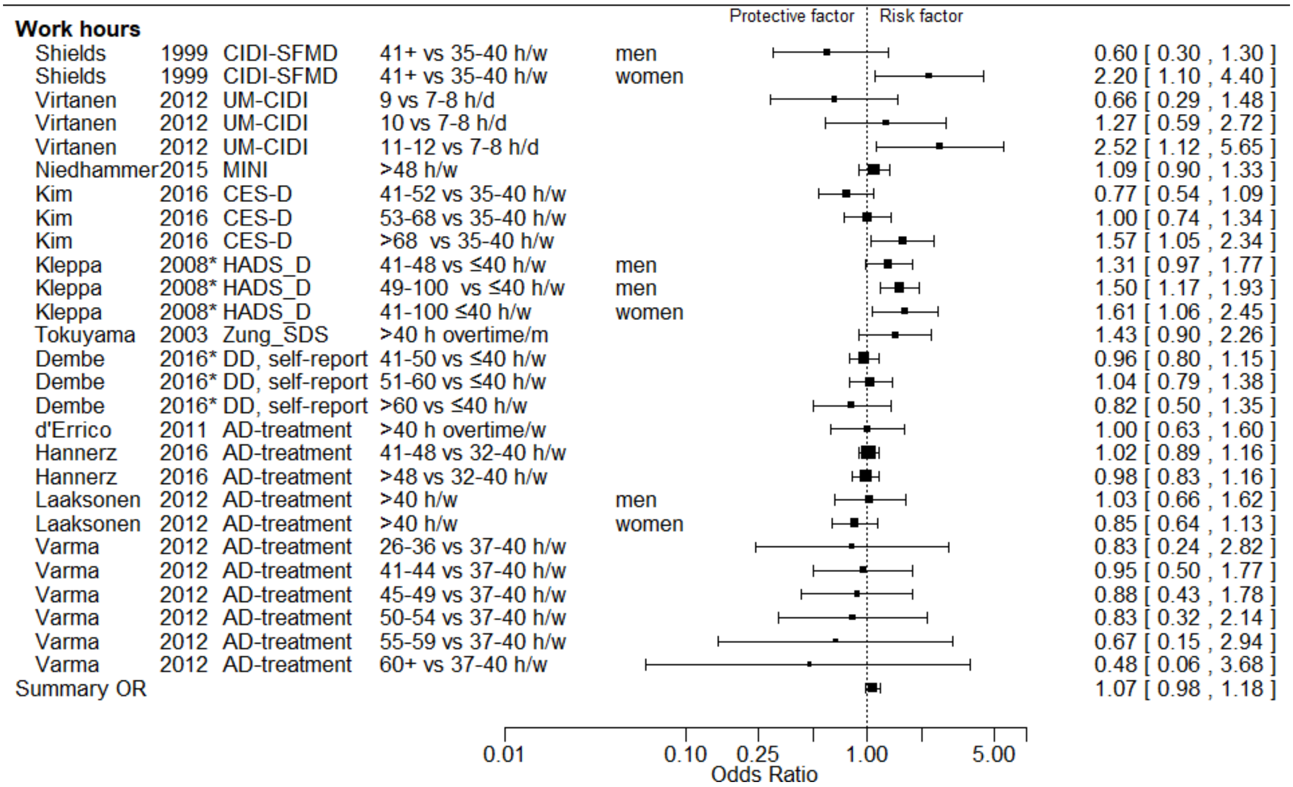

Fig A3.11 Forest plot for work hours (abbreviations, see textbox after last forest plot).

Textbox:
Forest plot abbreviations by column

Column		Abbreviation	Explanation
1	First author	Method of exposure ascertainment and first author	
2	Publication year	Asterisk (*) e.g. 2015*	Unless otherwise specified, a * means that the study is a cross-sectional study
3	Diagnostic instrument	SCAN	Schedules for Clinical Assessment in Neuropsychiatry [142]
		CIDI	Composite International Diagnostic Instrument [143]
		UM-CIDI	University of Michigan version of CIDI [144]
		CIDI-SFMD	Short Form version of CIDI [36, 145], Major Depression module
		CIDI-WMH	WHO Mental Health version of CIDI [146]
		MINI	Mini-International Neuropsychiatric Interview [147]
		CES-D_11	Center for Epidemiologic Studies Depression Scale [148], modified from 20 to 11 items
		HADS_D	Hospital Anxiety and Depression Scale (HADS), [149], Depression subscale
		MDI-algorithm	Major Depression Inventory [150].
		MDI-score	Major Depression Inventory [150].
		MHI-5	Mental Health Inventory [151]
		PHQ-9	Patient Health Questionnaire-9 [152]
		Zung SDS	Zung Selfrating Depression Scale [153]
		AD-treatment	Antidepressant treatment
		DD-self-report	Doctors diagnosis, self-reported
		DD-hospital	Doctors diagnosis, hospital records
4	Exposure contrast	cont. 1-4/1	Continuous on a scale from 1 to 4, by 1 scale unit
		cont./1 SD	Continuous by 1 SD
		median	Above vs below the median
		tertiles	Upper tertile vs lowest tertile
		tertile*	Upper tertile vs other tertiles combined
		quartiles	Upper quartile vs lowest quartile
		quartile*	Upper quartile vs other quartiles combined
		low/high or high/low	Dichotomies defined in various ways
		quadrant	See legend to job-strain Forest plot
5	Substudy/sub material		
6	Plots of risk estimates and their 95% confidence intervals		Risk estimates are relative risks, odds ratios and hazard ratios for measures of depression related to the highest exposure contrast and from the most adjusted analytical model. Since the outcome prevalence was low (<10%) we do not distinguish between relative risks, odds ratios and hazard ratios.
7	Figures corresponding to column 6		

Appendix 4. Validity of measures of depression

There is no *objective* 'gold' standard for psychiatric diagnoses. Instead, the standard against which case definitions are assessed is best evidence consensus of experts, based on structured clinical interviews, the longitudinal course of the illness and all other relevant information, the so-called LEAD-standard (Longitudinal, Expert, All Data) [154]. This standard is feasible in a clinical context, but not in large epidemiological studies of the general or working population.

In such studies we considered that the occurrence of one or more depressive episodes, as determined from a semi-structured diagnostic interview (if available) should be the standard against which the accuracy of other measures of depression should be assessed.

Fully structured interviews, especially the WHO Composite International Diagnostic Interview (CIDI) [143], are sometimes treated as an alternative standard. However, the CIDI is essentially a questionnaire, which originally was administered by an interviewer because of complex skip and decision rules but has since been adapted for self-administration in computerized versions. The CIDI exists in many versions, sometimes only validated in relation to other CIDI versions. Prevalence estimates of depression generated by the CIDI depression module have been criticised for being inflated by false-positive [155]. For these reasons we did not consider diagnoses of depressive episodes, or recurrent or major depressive disorders based on fully structured interviews as a standard comparable to semi-structured interviews.

We looked for reports on the validity of depression measures that had been used in the studies that were reviewed, both in the reference list of each study and through a search in PubMed with search terms for title and abstracts covering the specific depression measure (e.g. CIDI or 'primary care' and related terms), terms for validation, sensitivity and specificity, and terms for semi-structured interview methods (e.g. SCAN, SCID). Hits were screened by title, if relevant also by abstract, and finally by full text reading, and the reference lists of usable studies were checked for further relevant reports. These searches and the selection of relevant studies were carried out by one of the authors (SM)

Fully structured diagnostic interviews and questionnaire instruments

We estimated the validity of measures of depression based on fully structured interviews or questionnaire instruments, taking depressive episodes diagnosed by semi-structured interview as the standard. If available, we used sensitivities and specificities from studies of general or working populations to calculate false positive and false negative rates in a population with a true depressive episode prevalence of 5%, considered to be close to the true prevalence in a general population. If we found no studies of general or working populations, we used data from unselected patients in primary care or unselected in- or out-patients from non-psychiatric hospital settings.

The results showed that false positive rates varied between 60% and 89%, and false negative rates between 0.2% and 3% (Table A4.1).

Primary care diagnoses

Studies among representative samples of adult primary care patients to assess the validity of diagnostic criteria for depression in comparison with diagnoses based on semi-structured clinical interviews (taken as

a standard) have reported sensitivities ranging from 20% to 100% and specificities ranging from 69% to 94% [156-160]. In a population with a true prevalence of 5%, these sensitivities and specificities would imply false positive rates in the order of 80%-90% and false negative rates of <5%.

Self-report of depression diagnosed by a doctor

None of the studies included in the review focused directly on primary care diagnoses of depression. However, the outcomes in seven studies were based on self-report of depression diagnosed by a doctor, and 15 investigations controlled for previous depression using such self-report. Since the large majority of depression diagnoses are made in primary care, these outcome measures will suffer from the same inaccuracies as primary care diagnoses of depression, and in addition from limitations of recall of doctor- diagnoses many years back in time.

Population false positive and negative rates for *self-reported doctor's diagnoses of any previous depression* compared to lifetime prevalence of depressive episodes diagnosed by a semi-structured diagnostic interview, have been estimated in one study to be 25% and 19%, respectively, with a true prevalence of 26% [161]. However, the use of semi-structured interviews to assess lifetime prevalence is problematic because of the potential for error in recall of depressive symptoms many years in the past [162].

Furthermore, population studies indicate that fewer than half of participants with a depressive episode access the formal health care system about their depressive symptoms [163, 164], which will reduce the sensitivity of recalled doctor's diagnosis.

Thus, while the validity of self-reported doctor's diagnosis of depression as a measure of previous depressive episodes is uncertain, available data suggest that it may suffer from substantial inaccuracy.

Primary care diagnoses of depression are important determinants of antidepressant treatment and of referral for hospital examination and treatment. Thus, limitations and inaccuracies of primary care diagnoses of depression will extend also to these measures of depression.

Antidepressant treatment

Antidepressant treatment is mainly prescribed in primary care by general practitioners. The indications for prescribing antidepressant treatment were systematically examined in a nationwide Dutch study [165]. The study showed that depression/feeling depressed was the indication in only 45% of prescriptions Furthermore, a large European study has shown that only 30% of patients with a depressive episode, diagnosed by a semi-structured interview, were treated with antidepressants [166].

Routine hospital diagnoses

Studies on the validity of *routine hospital diagnoses* of depressive disorders and major depression, compared to diagnoses based on LEAD-criteria and best evidence consensus, including semi-

structured clinical interviews, have demonstrated false positive rates between 16% and 42%, and false negative rates between 5% and 31% [167-170]. In a systematic review of the accuracy of administratively collected diagnoses Davis et al.,2016 [171] found that the average false positive rate for affective disorders and unipolar depression (depressive episodes and dysthymia) was 25%. In a Danish study including routine discharge diagnoses of first-onset depressive episodes (ICD-10) from all psychiatric departments in eastern Denmark, the false positive rate was 24% compared to a subsequent semi-structured diagnostic interview [172].
Only a small proportion of persons with depressive episodes is treated in hospitals.

Diagnostic misclassification and bias

The null hypothesis of this review was that psychosocial exposures at work have no association with depressive episodes diagnosed by an acceptable standard (a semi-structured interview). However, studies used diagnostic methods that will have caused some misclassification with false positives and false negatives. If the true outcome is not associated with the exposure and if the misclassified cases are positively associated with exposure, then the risk estimate for a diagnosis of depression defined by the test will exceed unity. The size of this bias can be calculated for different circumstances involving specifications of the true prevalence of depressive episodes, the prevalence of a positive diagnostic test, the sensitivity of the test, the prevalence of exposure and the true relative risk of the association between misclassified cases and exposure.

As an example for circumstances similar to those of many studies included in this review: we set the true prevalence of depressive episodes to 3%, the prevalence of a positive diagnostic test to 5%, the sensitivity of the test to 75%, the prevalence of exposure to 25% and the true relative risk for the association between misclassified cases and exposure to 2.00. Under these circumstances, the relative risk of being diagnosed with depression by the test will be 1.49 even if the true relative risk is equal to unity. If the true relative risk of the association between misclassified cases and exposure is set to 1.50, then the relative risk of being diagnosed with depression by the test will be 1.26 even if the true relative risk is equal to unity. In both examples the false positive rate was 55%.

Meta-analytic estimates of relative risks of depressive *symptoms* by psychosocial factors at work have recently been examined in a systematic review, and many were found to be in the order of 1.5 to 2.0 for adverse high levels of exposure compared to low levels [173].

Against this background it seems quite plausible that relative risks up to between 1.25 and 1.50 could be explained by bias due to diagnostic false positives with depressive symptoms.

**Table A4.1 Sensitivity and specificity of diagnostic methods used to assess current to 12-month occurrence of depressive episode or mood disorders/depressive disorders compared to a semi-structured diagnostic interview, and calculated false positive and negative rates for a true prevalence of 5%.** (DE: depressive episode ICD-classification, MDE: major depressive episode DSM-classification; nr: not reported)								
**Diagnostic method, First author, year**	**Material**	**Outcome**	**Test method**	**Re-ference method**	**Sensiti-vity**	**Specifi-city**	**False positive rate**	**False negative rate**
CIDI								
Brugha 2001 [174]	General population	DE	CIDI-Auto 1.1	SCAN	0.50	0.90	0.83	0.03
Kessler 2003 [175]	General population	MDE	CIDI	SCID	0.55	0.95	0.65	0.02
Aalto-Setälä 2002 [176]	Working population	DE	CIDI-SF	SCAN	0.73	0.82	0.83	0.02
MINI								
de Azevedo Marques 2008 [177]	Primary care patients	MDE	MINI	SCID	0.90	0.93	0.60	0.01
CES-D								
Jahn 2018 [178]	General population	DE	CES-D≥10	SCAN	0.81	0.74	0.86	0.01
Campo-Arias 2007, cited from Vilagut 2016 [179]	General population	MDE	CES-D (nr)	SCID	0.98	0.58	0.89	0.002
HADS-D								
Abiodun 1994 [180]	General population	DE	HADS-D≥8	PSE*	0.90	0.91	0.66	0.01
Kjærgaard 2014 [181]	General population	MDE	HADS-D≥4	SCID	0,.85	0.79	0.81	0.01
MDI								
Forsell 2005 [182]	General population	DE	MDI-algorithm	SCAN	0.67	0.81	0.84	0.02
MHI-5								
Rumpf et. al. 2001 [183]	General population	Mood disorders	MHI-5<52	SCID	0.72	0.86	0.79	0.02
PHQ-9								
Kroenke 2001 [152]	Primary care patients	MDE	PHQ-9≥10	Adapted SCID	0.88	0.88	0.72	0.01
Patten 2015 [184]	Out-patients, multiple sclerosis clinic	MDE	PHQ-9≥10	SCID	0.95	0.88	0.71	0.003
Lowe 2004 [185]	Outpatients medical hospital and primary care	MDE	PHQ-9≥12	SCID	0.95	0.84	0.76	0.003
Zung SDS								
Campo-Arias 2006 [186]	General population	MDE	Zung SDS ≥40	SCID	88.6	74.8	0.84	0.01

*Present State Examination (a forerunner for SCAN)

References

1. Barrech A, Riedel N, Li J, Herr RM, Mortl K, Angerer P et al. The long-term impact of a change in Effort-Reward imbalance on mental health-results from the prospective MAN-GO study. European Journal of Public Health. 2017;27(6):1021-6. doi:10.1093/eurpub/ckx068.
2. de Lange AH, Taris TW, Kompier MA, Houtman IL, Bongers PM. Effects of stable and changing demand-control histories on worker health. Scand J Work Environ Health. 2002;28(2):94-108. doi:653 [pii].
3. Ferrie JE, Shipley MJ, Newman K, Stansfeld SA, Marmot M. Self-reported job insecurity and health in the Whitehall II study: potential explanations of the relationship. Social Science & Medicine. 2005;60(7):1593-602. doi:10.1016/j.socscimed.2004.08.006.
4. Greenglass ER, Burke RJ. Stress and the effects of hospital restructuring in nurses. CJNR: Canadian Journal of Nursing Research. 2001;33(2):93-108.
5. Idris MA, Dollard MF, Yulita. Psychosocial safety climate, emotional demands, burnout, and depression: A longitudinal multilevel study in the Malaysian private sector. Journal of Occupational Health Psychology. 2014;19(3):291-302. doi:10.1037/a0036599.
6. Lamy S, De Gaudemaris R, Lepage B, Sobaszek A, Caroly S, Kelly-Irving M et al. The organizational work factors' effect on mental health among hospital workers is mediated by perceived effort–reward imbalance: Result of a longitudinal study. Journal of Occupational and Environmental Medicine. 2013;55(7):809-16. doi:10.1097/JOM.0b013e31828acb19.
7. Lee HY, Kim MS, Kim O, Lee IH, Kim HK. Association between shift work and severity of depressive symptoms among female nurses: the Korea Nurses' Health Study. J Nurs Manag. 2016;24(2):192-200. doi:10.1111/jonm.12298.
8. Magnusson Hanson LL, Chungkham HS, Akerstedt T, Westerlund H. The role of sleep disturbances in the longitudinal relationship between psychosocial working conditions, measured by work demands and support, and depression. Sleep. 2014;37(12):1977-85. doi:10.5665/sleep.4254.
9. Moore S, Grunberg L, Greenberg E. Repeated downsizing contact: The effects of similar and dissimilar layoff experiences on work and well-being outcomes. Journal of Occupational Health Psychology. 2004;9(3):247-57. doi:10.1037/1076-8998.9.3.247.
10. Muntaner C, Li Y, Xue XN, Thompson T, O'Campo P, Chung H et al. County level socioeconomic position, work organization and depression disorder: A repeated measures cross-classified multilevel analysis of low-income nursing home workers. Health & Place. 2006;12(4):688-700. doi:10.1016/j.healthplace.2005.09.004.
11. Nyberg A, Peristera P, Westerlund H, Johansson G, Hanson LLM. Does job promotion affect men's and women's health differently? Dynamic panel models with fixed effects. International Journal of Epidemiology. 2017;46(4):1137-46. doi:10.1093/ije/dyw310.
12. Paterniti S, Niedhammer I, Lang T, Consoli SM. Psychosocial factors at work, personality traits and depressive symptoms. Longitudinal results from the GAZEL Study. Br J Psychiatry. 2002;181:111-7.
13. Peter R, March S, du Prel JB. Are status inconsistency, work stress and work-family conflict associated with depressive symptoms? Testing prospective evidence in the lidA study. Soc Sci Med. 2016;151:100-9. doi:10.1016/j.socscimed.2016.01.009.
14. Shirangi A, Fritschi L, Holman CD, Morrison D. Mental health in female veterinarians: effects of working hours and having children. Aust Vet J. 2013;91(4):123-30. doi:10.1111/avj.12037.
15. Takahashi M, Iwasaki K, Sasaki T, Kubo T, Mori I, Otsuka Y. Sleep, fatigue, recovery, and depression after change in work time control: a one-year follow-up study. J Occup Environ Med. 2012;54(9):1078-85. doi:10.1097/JOM.0b013e31826230b7.
16. Taris TW. The mutual effects between job resources and mental health: A prospective study among Dutch youth. Genetic, Social, and General Psychology Monographs. 1999;125(4):433-50.
17. Theorell T, Hammarstrom A, Gustafsson PE, Hanson LM, Janlert U, Westerlund H. Job strain and depressive symptoms in men and women: a prospective study of the working population in Sweden. Journal of Epidemiology and Community Health. 2014;68(1):78-82. doi:10.1136/jech-2012-202294.

18. Tornroos M, Elovainio M, Keltikangas-Jarvinen L, Hintsa T, Pulkki-Raback L, Hakulinen C et al. Is There a Two-Way Relationship Between Cynicism and Job Strain? Evidence From a Prospective Population-Based Study. Journal of Occupational and Environmental Medicine. 2015;57(5):479-84. doi:10.1097/jom.0000000000000430.
19. Uchida M, Morita H. Association between long work hours and depressive state: a pilot study of propensity score matched Japanese white-collar workers. Industrial Health. 2018;56(3):207-11. doi:10.2486/indhealth.2017-0139.
20. Woodward CA, Shannon HS, Cunningham C, McIntosh J, Lendrum B, Rosenbloom D et al. The impact of re-engineering and other cost reduction strategies on the staff of a large teaching hospital - A longitudinal study. Medical Care. 1999;37(6):556-69. doi:10.1097/00005650-199906000-00005.
21. Waage S, Pallesen S, Moen BE, Magerøy N, Flo E, Di Milia L et al. Predictors of shift work disorder among nurses: A longitudinal study. Sleep Medicine. 2014;15(12):1449-55. doi:10.1016/j.sleep.2014.07.014.
22. Özdemir PG, Selvi Y, Özkol H, Aydın A, Tülüce Y, Boysan M et al. The influence of shift work on cognitive functions and oxidative stress. Psychiatry Research. 2013;210(3):1219-25. doi:10.1016/j.psychres.2013.09.022.
23. Airila A, Hakanen JJ, Luukkonen R, Lusa S, Punakallio A, Leino-Arjas P. Developmental trajectories of multisite musculoskeletal pain and depressive symptoms: The effects of job demands and resources and individual factors. Psychology & Health. 2014;29(12):1421-41. doi:10.1080/08870446.2014.945929.
24. Andrea H, Bültmann U, van Amelsvoort LGPM, Kant Y. The incidence of anxiety and depression among employees—The role of psychosocial work characteristics. Depression and Anxiety. 2009;26(11):1040-8. doi:10.1002/da.20516.
25. Berthelsen M, Pallesen S, Mageroy N, Tyssen R, Bjorvatn B, Moen BE et al. Effects of Psychological and Social Factors in Shiftwork on Symptoms of Anxiety and Depression in Nurses: A 1-Year Follow-Up. J Occup Environ Med. 2015;57(10):1127-37. doi:10.1097/jom.0000000000000532.
26. Burns RA, Butterworth P, Anstey KJ. An examination of the long-term impact of job strain on mental health and wellbeing over a 12-year period. Social Psychiatry and Psychiatric Epidemiology: The International Journal for Research in Social and Genetic Epidemiology and Mental Health Services. 2016;51(5):725-33. doi:10.1007/s00127-016-1192-9.
27. Butterworth P, Leach LS, Kiely KM. Why it's important for it to stop: Examining the mental health correlates of bullying and ill-treatment at work in a cohort study. Aust N Z J Psychiatry. 2016;50(11):1085-95. doi:10.1177/0004867415622267.
28. Einarsen S, Nielsen MB. Workplace bullying as an antecedent of mental health problems: a five-year prospective and representative study. Int Arch Occup Environ Health. 2015;88(2):131-42. doi:10.1007/s00420-014-0944-7.
29. Emdad R, Alipour A, Hagberg J, Jensen IB. The impact of bystanding to workplace bullying on symptoms of depression among women and men in industry in Sweden: an empirical and theoretical longitudinal study. Int Arch Occup Environ Health. 2013;86(6):709-16. doi:10.1007/s00420-012-0813-1.
30. Geiger-Brown J, Muntaner C, McPhaul K, Lipscomb J, Trinkoff A. Abuse and violence during home care work as predictor of worker depression. Home Health Care Services Quarterly. 2007;26(1):59-77. doi:10.1300/J027v26n01_05.
31. Kivimaki M, Vahtera J, Elovainio M, Virtanen M, Siegrist J. Effort-reward imbalance, procedural injustice and relational injustice as psychosocial predictors of health: complementary or redundant models? Occupational and Environmental Medicine. 2007;64(10):659-65. doi:10.1136/oem.2006.031310.
32. Nyberg A, Hanson LLM, Leineweber C, Hammarstrom A, Theorell T. Occupational gender composition and mild to severe depression in a Swedish cohort: The impact of psychosocial work factors. Scandinavian Journal of Public Health. 2018;46(3):425-32. doi:10.1177/1403494817745736.
33. Stansfeld SA, Head J, Fuhrer R, Wardle J, Cattell V. Social inequalities in depressive symptoms and physical functioning in the Whitehall II study: exploring a common cause explanation. Journal of Epidemiology and Community Health. 2003;57(5):361-7. doi:10.1136/jech.57.5.361.

34. Waldenstrom K, Ahlberg G, Bergman P, Forsell Y, Stoetzer U, Waldenstrom M et al. Externally assessed psychosocial work characteristics and diagnoses of anxiety and depression. Occup Environ Med. 2008;65(2):90-6. doi:10.1136/oem.2006.031252.
35. Virtanen M, Koskinen S, Kivimaki M, Honkonen T, Vahtera J, Ahola K et al. Contribution of non-work and work-related risk factors to the association between income and mental disorders in a working population: the Health 2000 Study. Occupational and Environmental Medicine. 2008;65(3):171-8. doi:10.1136/oem.2007.033159.
36. Kessler RC, McGonagle KA, Zhao S, Nelson CB, Hughes M, Eshleman S et al. Lifetime and 12-month prevalence of DSM-III-R psychiatric disorders in the United States. Results from the National Comorbidity Survey. Arch Gen Psychiatry. 1994;51(1):8-19.
37. Pedersen CB, Mors O, Bertelsen A, Waltoft BL, Agerbo E, McGrath JJ et al. A comprehensive nationwide study of the incidence rate and lifetime risk for treated mental disorders. JAMA Psychiatry. 2014;71(5):573-81. doi:10.1001/jamapsychiatry.2014.16.
38. Joensuu M, Vaananen A, Koskinen A, Kivimaki M, Virtanen M, Vahtera J. Psychosocial work environment and hospital admissions due to mental disorders: a 15-year prospective study of industrial employees. J Affect Disord. 2010;124(1-2):118-25. doi:S0165-0327(09)00485-6 [pii];10.1016/j.jad.2009.10.025 [doi].
39. Plaisier I, de Bruijn JG, de Graaf R, ten Have M, Beekman AT, Penninx BW. The contribution of working conditions and social support to the onset of depressive and anxiety disorders among male and female employees. Soc Sci Med. 2007;64(2):401-10. doi:10.1016/j.socscimed.2006.09.008.
40. Wieclaw J, Agerbo E, Mortensen PB, Burr H, Tuchsen F, Bonde JP. Psychosocial working conditions and the risk of depression and anxiety disorders in the Danish workforce. BMC Public Health. 2008;8:280. doi:10.1186/1471-2458-8-280.
41. Wieclaw J, Agerbo E, Mortensen PB, Burr H, Tuchsen F, Bonde JP. Work related violence and threats and the risk of depression and stress disorders. J Epidemiol Community Health. 2006;60(9):771-5. doi:10.1136/jech.2005.042986.
42. Ahlin JK, Rajaleid K, Jansson-Frojmark M, Westerlund H, Magnusson Hanson LL. Job demands, control and social support as predictors of trajectories of depressive symptoms. J Affect Disord. 2018;235:535-43. doi:10.1016/j.jad.2018.04.067.
43. Ahlin JK, Westerlund H, Griep Y, Hanson LLM. Trajectories of job demands and control: risk for subsequent symptoms of major depression in the nationally representative Swedish Longitudinal Occupational Survey of Health (SLOSH). International Archives of Occupational and Environmental Health. 2018;91(3):263-72. doi:10.1007/s00420-017-1277-0.
44. Albrecht SC, Kecklund G, Rajaleid K, Leineweber C. The longitudinal relationship between control over working hours and depressive symptoms: Results from SLOSH, a population-based cohort study. Journal of Affective Disorders. 2017;215:143-51. doi:10.1016/j.jad.2017.03.010.
45. Andreeva E, Hanson LLM, Westerlund H, Theorell T, Brenner MH. Depressive symptoms as a cause and effect of job loss in men and women: evidence in the context of organisational downsizing from the Swedish Longitudinal Occupational Survey of Health. BMC Public Health. 2015;15. doi:10.1186/s12889-015-2377-y.
46. Andreeva E, Brenner MH, Theorell T, Goldberg M. Risk of psychological ill health and methods of organisational downsizing: a cross-sectional survey in four European countries. BMC Public Health. 2017;17(1):758. doi:10.1186/s12889-017-4789-3.
47. Brenner MH, Andreeva E, Theorell T, Goldberg M, Westerlund H, Leineweber C et al. Organizational downsizing and depressive symptoms in the European recession: the experience of workers in France, Hungary, Sweden and the United kingdom. PLoS ONE. 2014;9(5):e97063. doi:10.1371/journal.pone.0097063.
48. Hanson LLM, Chungkham HS, Ferrie J, Sverke M. Threats of dismissal and symptoms of major depression: a study using repeat measures in the Swedish working population. Journal of Epidemiology and Community Health. 2015;69(10):963-9. doi:10.1136/jech-2014-205405.

49. Amagasa T, Nakayama T. Relationship between long working hours and depression: A 3-year longitudinal study of clerical workers. Journal of Occupational and Environmental Medicine. 2013;55(8):863-72. doi:10.1097/JOM.0b013e31829b27fa.
50. Driesen K, Jansen NW, van Amelsvoort LG, Kant I. The mutual relationship between shift work and depressive complaints--a prospective cohort study. Scand J Work Environ Health. 2011;37(5):402-10. doi:10.5271/sjweh.3158.
51. Kim I, Kim H, Lim S, Lee M, Bahk J, Ja June K et al. Working hours and depressive symptomatology among full-time employees: Results from the fourth Korean National Health and Nutrition Examination Survey (2007–2009). Scandinavian Journal of Work, Environment & Health. 2013;39(5):515-20. doi:10.5271/sjweh.3356.
52. Weigl M, Hornung S, Petru R, Glaser J, Angerer P. Depressive symptoms in junior doctors: a follow-up study on work-related determinants. Int Arch Occup Environ Health. 2012;85(5):559-70. doi:10.1007/s00420-011-0706-8.
53. Fujiwara T, Kawachi I. A prospective study of individual-level social capital and major depression in the United States. Journal of Epidemiology and Community Health. 2008;62(7):627-33. doi:10.1136/jech.2007.064261.
54. Lee HE, Kim HR, Kong JO, Jang TW, Myong JP, Koo JW et al. Weekend work and depressive symptoms among Korean employees. Chronobiol Int. 2015;32(2):262-9. doi:10.3109/07420528.2014.965826.
55. Sen S, Kranzler HR, Krystal JH, Speller H, Chan G, Gelernter J et al. A prospective cohort study investigating factors associated with depression during medical internship. Arch Gen Psychiatry. 2010;67(6):557-65. doi:10.1001/archgenpsychiatry.2010.41.
56. Godin I, Kornitzer M, Clumeck N, Linkowski P, Valente F, Kittel F. Gender specificity in the prediction of clinically diagnosed depression: Results of a large cohort of Belgian workers. Social Psychiatry and Psychiatric Epidemiology: The International Journal for Research in Social and Genetic Epidemiology and Mental Health Services. 2009;44(7):592-600. doi:10.1007/s00127-008-0465-3.
57. DeSanto Iennaco J, Cullen MR, Cantley L, Slade MD, Fiellin M, Kasl SV. Effects of externally rated job demand and control on depression diagnosis claims in an industrial cohort. Am J Epidemiol. 2010;171(3):303-11. doi:10.1093/aje/kwp359.
58. Alterman T, Grosch J, Chen X, Chrislip D, Petersen M, Krieg E et al. Examining Associations Between Job Characteristics and Health: Linking Data From the Occupational Information Network (O*NET) to Two US National Health Surveys. Journal of Occupational and Environmental Medicine. 2008;50(12):1401-13. doi:10.1097/JOM.0b013e318188e882.
59. Dragano N, He Y, Moebus S, Jockel KH, Erbel R, Siegrist J. Two models of job stress and depressive symptoms. Results from a population-based study. Soc Psychiatry Psychiatr Epidemiol. 2008;43(1):72-8. doi:10.1007/s00127-007-0267-z.
60. Mausner-Dorsch H, Eaton WW. Psychosocial work environment and depression: Epidemiologic assessment of the demand-control model. American Journal of Public Health. 2000;90(11):1765-70. doi:10.2105/AJPH.90.11.1765.
61. Melchior M, Caspi A, Milne BJ, Danese A, Poulton R, Moffitt TE. Work stress precipitates depression and anxiety in young, working women and men. Psychological Medicine. 2007;37(8):1119-29. doi:10.1017/S0033291707000414.
62. Mezuk B, Bohnert AS, Ratliff S, Zivin K. Job strain, depressive symptoms, and drinking behavior among older adults: results from the health and retirement study. J Gerontol B Psychol Sci Soc Sci. 2011;66(4):426-34. doi:10.1093/geronb/gbr021.
63. Nakao M, Yano E. A comparative study of behavioural, physical and mental health status between term-limited and tenure-tracking employees in a population of Japanese mate researchers. Public Health. 2006;120(4):373-9. doi:10.1016/j.puhe.2005.10.012.
64. Stansfeld SA, Clark C, Caldwell T, Rodgers B, Power C. Psychosocial work characteristics and anxiety and depressive disorders in midlife: the effects of prior psychological distress. Occup Environ Med. 2008;65(9):634-42. doi:10.1136/oem.2007.036640.

65. Ibrahim S, Smith P, Muntaner C. A multi-group cross-lagged analyses of work stressors and health using Canadian national sample. Social Science & Medicine. 2009;68(1):49-59. doi:10.1016/j.socscimed.2008.10.019.
66. Ahola K, Vuori J, Toppinen-Tanner S, Mutanen P, Honkonen T. Resource-enhancing group intervention against depression at workplace: who benefits? A randomised controlled study with a 7-month follow-up. Occup Environ Med. 2012;69(12):870-6. doi:10.1136/oemed-2011-100450.
67. Ahola K, Hakanen J. Job strain, burnout, and depressive symptoms: A prospective study among dentists. Journal of Affective Disorders. 2007;104(1-3):103-10. doi:10.1016/j.jad.2007.03.004.
68. Amagasa T, Nakayama T. Relationship between long working hours and depression in two working populations: A structural equation model approach. Journal of Occupational and Environmental Medicine. 2012;54(7):868-74. doi:10.1097/JOM.0b013e318250ca00.
69. Clays E, De Bacquer D, Eynen FL, Kornitzer M, Kittel F, De Backer G. Job stress and depression symptoms in middle-aged workers - prospective results from the Belstress study. Scandinavian Journal of Work Environment & Health. 2007;33(4):252-9. doi:10.5271/sjweh.1140.
70. Griffin JM, Fuhrer R, Stansfeld SA, Marmot M. The importance of low control at work and home on depression and anxiety: do these effects vary by gender and social class? Soc Sci Med. 2002;54(5):783-98.
71. Godin I, Kittel F, Coppieters Y, Siegrist J. A prospective study of cumulative job stress in relation to mental health. BMC Public Health. 2005;5. doi:10.1186/1471-2458-5-67.
72. Kato R, Haruyama Y, Endo M, Tsutsumi A, Muto T. Heavy overtime work and depressive disorder among male workers. Occup Med (Lond). 2014;64(8):622-8. doi:10.1093/occmed/kqu120.
73. Kim MS, Hong YC, Yook JH, Kang MY. Effects of perceived job insecurity on depression, suicide ideation, and decline in self-rated health in Korea: a population-based panel study. Int Arch Occup Environ Health. 2017;90(7):663-71. doi:10.1007/s00420-017-1229-8.
74. Kim Y, Kim SS. Job insecurity and depression among automobile sales workers: A longitudinal study in South Korea. American Journal of Industrial Medicine. 2018;61(2):140-7. doi:10.1002/ajim.22805.
75. Meneton P, Lemogne C, Herquelot E, Bonenfant S, Czernichow S, Menard J et al. Primary Cardiovascular Disease Risk Factors Predicted by Poor Working Conditions in the GAZEL Cohort. American Journal of Epidemiology. 2017;186(7):815-23. doi:10.1093/aje/kwx152.
76. Nakata A. Work hours, sleep sufficiency, and prevalence of depression among full-time employees: a community-based cross-sectional study. J Clin Psychiatry. 2011;72(5):605-14. doi:10.4088/JCP.10m06397gry.
77. Netterstrom B, Blond M, Nielsen M, Rugulies R, Eskelinen L. Development of depressive symptoms and depression during organizational change--a two-year follow-up study of civil servants. Scand J Work Environ Health. 2010;36(6):445-8.
78. Niedhammer I, Goldberg M, Leclerc A, Bugel I, David S. Psychosocial factors at work and subsequent depressive symptoms in the Gazel cohort. Scand J Work Environ Health. 1998;24(3):197-205.
79. Tomioka K, Morita N, Saeki K, Okamoto N, Kurumatani N. Working hours, occupational stress and depression among physicians. Occupational Medicine. 2011;61(3):163-70. doi:10.1093/occmed/kqr004.
80. Virtanen M, Ferrie JE, Batty GD, Elovainio M, Jokela M, Vahtera J et al. Socioeconomic and psychosocial adversity in midlife and depressive symptoms post retirement: a 21-year follow-up of the Whitehall II study. Am J Geriatr Psychiatry. 2015;23(1):99-109.e1. doi:10.1016/j.jagp.2014.04.001.
81. Fushimi M, Saito S, Shimizu T. Prevalence of depressive symptoms and related factors in Japanese employees as measured by the Center for Epidemiologic Studies Depression Scale (CES-D). Community mental health journal. 2013;49(2):236-42. doi:10.1007/s10597-012-9542-x.
82. Magnusson Hanson LL, Peristera P, Chungkham HS, Westerlund H. Longitudinal Mediation Modeling of Unhealthy Behaviors as Mediators between Workplace Demands/Support and Depressive Symptoms. PLoS ONE. 2016;11(12):e0169276. doi:10.1371/journal.pone.0169276.
83. Magnusson Hanson LL, Peristera P, Chungkham HS, Westerlund H. Psychosocial work characteristics, sleep disturbances and risk of subsequent depressive symptoms: a study of time-varying effect modification. J Sleep Res. 2017;26(3):266-76. doi:10.1111/jsr.12494.

84. Madsen IE, Jorgensen AF, Borritz M, Nielsen ML, Rugulies R. Is the association between high strain work and depressive symptoms modified by private life social support: a cohort study of 1,074 Danish employees? BMC Public Health. 2014;14:698. doi:10.1186/1471-2458-14-698.
85. Lunau T, Wahrendorf M, Muller A, Wright B, Dragano N. Do resources buffer the prospective association of psychosocial work stress with depression? Longitudinal evidence from ageing workers. Scand J Work Environ Health. 2018;44(2):183-91. doi:10.5271/sjweh.3694.
86. Wege N, Li J, Siegrist J. Are there gender differences in associations of effort-reward imbalance at work with self-reported doctor-diagnosed depression? Prospective evidence from the German Socio-Economic Panel. Int Arch Occup Environ Health. 2018;91(4):435-43. doi:10.1007/s00420-018-1293-8.
87. Rugulies R, Aust B, Madsen IE, Burr H, Siegrist J, Bultmann U. Adverse psychosocial working conditions and risk of severe depressive symptoms. Do effects differ by occupational grade? Eur J Public Health. 2013;23(3):415-20. doi:10.1093/eurpub/cks071.
88. Siegrist J, Lunau T, Wahrendorf M, Dragano N. Depressive symptoms and psychosocial stress at work among older employees in three continents. Global Health. 2012;8:27. doi:10.1186/1744-8603-8-27.
89. Dragano N, Siegrist J, Wahrendorf M. Welfare regimes, labour policies and unhealthy psychosocial working conditions: A comparative study with 9917 older employees from 12 European countries. Journal of Epidemiology and Community Health. 2011;65(9):793-9. doi:10.1136/jech.2009.098541.
90. Lunau T, Wahrendorf M, Dragano N, Siegrist J. Work stress and depressive symptoms in older employees: impact of national labour and social policies. BMC Public Health. 2013;13:1086. doi:10.1186/1471-2458-13-1086.
91. Oksanen T, Kouvonen A, Vahtera J, Virtanen M, Kivimaki M. Prospective study of workplace social capital and depression: are vertical and horizontal components equally important? Journal of Epidemiology and Community Health. 2010;64(8):684-9. doi:10.1136/jech.2008.086074.
92. Kouvonen A, Oksanen T, Vahtera J, Stafford M, Wilkinson R, Schneider J et al. Low workplace social capital as a predictor of depression: the Finnish Public Sector Study. Am J Epidemiol. 2008;167(10):1143-51. doi:10.1093/aje/kwn067.
93. Sinokki M, Hinkka K, Ahola K, Koskinen S, Kivimaki M, Honkonen T et al. The association of social support at work and in private life with mental health and antidepressant use: the Health 2000 Study. J Affect Disord. 2009;115(1-2):36-45. doi:10.1016/j.jad.2008.07.009.
94. Sinokki M, Hinkka K, Ahola K, Koskinen S, Klaukka T, Kivimaki M et al. The association between team climate at work and mental health in the Finnish Health 2000 Study. Occupational and Environmental Medicine. 2009;66(8):523-8. doi:10.1136/oem.2008.043299.
95. Virtanen M, Ferrie JE, Singh-Manoux A, Shipley MJ, Stansfeld SA, Marmot MG et al. Long working hours and symptoms of anxiety and depression: a 5-year follow-up of the Whitehall II study. Psychol Med. 2011;41(12):2485-94. doi:10.1017/s0033291711000171.
96. Virtanen M, Stansfeld SA, Fuhrer R, Ferrie JE, Kivimaki M. Overtime work as a predictor of major depressive episode: a 5-year follow-up of the Whitehall II study. PLoS ONE. 2012;7(1):e30719. doi:10.1371/journal.pone.0030719.
97. Wang J, Schmitz N. Does job strain interact with psychosocial factors outside of the workplace in relation to the risk of major depression? The Canadian National Population Health Survey. Social Psychiatry and Psychiatric Epidemiology: The International Journal for Research in Social and Genetic Epidemiology and Mental Health Services. 2011;46(7):577-84. doi:10.1007/s00127-010-0224-0.
98. Shields M. Stress and depression in the employed population. Health Rep. 2006;17(4):11-29.
99. Stoetzer U, Ahlberg G, Johansson G, Bergman P, Hallsten L, Forsell Y et al. Problematic interpersonal relationships at work and depression: a Swedish prospective cohort study. J Occup Health. 2009;51(2):144-51.
100. Fandino-Losada A, Forsell Y, Lundberg I. Demands, skill discretion, decision authority and social climate at work as determinants of major depression in a 3-year follow-up study. Int Arch Occup Environ Health. 2013;86(5):591-605. doi:10.1007/s00420-012-0791-3.

101. Gullander M, Hogh A, Hansen AM, Persson R, Rugulies R, Kolstad HA et al. Exposure to workplace bullying and risk of depression. J Occup Environ Med. 2014;56(12):1258-65. doi:10.1097/jom.0000000000000339.
102. Hogh A, Conway PM, Grynderup MB, Gullander M, Willert MV, Mikkelsen EG et al. Negative Acts at Work as Potential Bullying Behavior and Depression: Examining the Direction of the Association in a 2-Year Follow-Up Study. J Occup Environ Med. 2016;58(3):e72-9. doi:10.1097/jom.0000000000000622.
103. Bonde JP, Gullander M, Hansen AM, Grynderup M, Persson R, Hogh A et al. Health correlates of workplace bullying: a 3-wave prospective follow-up study. Scand J Work Environ Health. 2016;42(1):17-25. doi:10.5271/sjweh.3539.
104. Shields M. Long working hours and health. Health Rep. 1999;11(2):33-48.
105. Wang J. Perceived Work Stress and Major Depressive Episodes in a Population of Employed Canadians Over 18 Years Old. Journal of Nervous and Mental Disease. 2004;192(2):160-3. doi:10.1097/01.nmd.0000110242.97744.bc.
106. Wang J, Schmitz N, Dewa C, Stansfeld S. Changes in perceived job strain and the risk of major depression: results from a population-based longitudinal study. Am J Epidemiol. 2009;169(9):1085-91. doi:10.1093/aje/kwp037.
107. Smith PM, Bielecky A. The impact of changes in job strain and its components on the risk of depression. American Journal of Public Health. 2012;102(2):352-8. doi:10.2105/AJPH.2011.300376.
108. Wang J, Patten SB, Currie S, Sareen J, Schmitz N. A population-based longitudinal study on work environmental factors and the risk of major depressive disorder. Am J Epidemiol. 2012;176(1):52-9. doi:10.1093/aje/kwr473.
109. Nigatu YT, Wang J. The combined effects of job demand and control, effort-reward imbalance and work-family conflicts on the risk of major depressive episode: a 4-year longitudinal study. Occup Environ Med. 2018;75(1):6-11. doi:10.1136/oemed-2016-104114.
110. Madsen IE, Diderichsen F, Burr H, Rugulies R. Person-related work and incident use of antidepressants: relations and mediating factors from the Danish work environment cohort study. Scand J Work Environ Health. 2010;36(6):435-44.
111. Hanson LLM, Madsen IEH, Westerlund H, Theorell T, Burr H, Rugulies R. Antidepressant use and associations with psychosocial work characteristics. A comparative study of Swedish and Danish gainfully employed. Journal of Affective Disorders. 2013;149(1-3):38-45. doi:10.1016/j.jad.2012.08.023.
112. Stansfeld SA, Shipley MJ, Head J, Fuhrer R. Repeated job strain and the risk of depression: Longitudinal analyses from the Whitehall II Study. American Journal of Public Health. 2012;102(12):2360-6. doi:10.2105/AJPH.2011.300589.
113. Bonde JPE, Munch-Hansen T, Wieclaw J, Westergaard-Nielsen N, Agerbo E. Psychosocial work environment and antidepressant medication: a prospective cohort study. BMC Public Health. 2009;9. doi:10.1186/1471-2458-9-262.
114. Dembe AE, Yao XX. Chronic Disease Risks From Exposure to Long-Hour Work Schedules Over a 32-Year Period. Journal of Occupational and Environmental Medicine. 2016;58(9):861-7. doi:10.1097/jom.0000000000000810.
115. Dement JM, Lipscomb HJ, Schoenfisch AL, Pompeii LA. Impact of hospital Type II violent events: Use of psychotropic drugs and mental health services. American Journal of Industrial Medicine. 2014;57(6):627-39. doi:10.1002/ajim.22306.
116. d'Errico A, Cardano M, Landriscina T, Marinacci C, Pasian S, Petrelli A et al. Workplace stress and prescription of antidepressant medications: a prospective study on a sample of Italian workers. International Archives of Occupational and Environmental Health. 2011;84(4):413-24. doi:10.1007/s00420-010-0586-3.
117. Grynderup MB, Mors O, Hansen AM, Andersen JH, Bonde JP, Kaergaard A et al. A two-year follow-up study of risk of depression according to work-unit measures of psychological demands and decision latitude. Scand J Work Environ Health. 2012;38(6):527-36. doi:10.5271/sjweh.3316.

118. Grynderup MB, Mors O, Hansen AM, Andersen JH, Bonde JP, Kaergaard A et al. Work-unit measures of organisational justice and risk of depression--a 2-year cohort study. Occup Environ Med. 2013;70(6):380-5. doi:10.1136/oemed-2012-101000.
119. Hall AL, Franche RL, Koehoorn M. Examining Exposure Assessment in Shift Work Research: A Study on Depression Among Nurses. Annals of Work Exposures and Health. 2018;62(2):182-94. doi:10.1093/annweh/wxx103.
120. Hannerz H, Albertsen K. Long working hours and use of psychotropic medicine: a follow-up study with register linkage. Scandinavian Journal of Work Environment & Health. 2016;42(2):153-61. doi:10.5271/sjweh.3550.
121. Kim W, Park EC, Lee TH, Kim TH. Effect of working hours and precarious employment on depressive symptoms in South Korean employees: a longitudinal study. Occup Environ Med. 2016;73(12):816-22. doi:10.1136/oemed-2016-103553.
122. Kivimaki M, Virtanen M, Vartia M, Elovainio M, Vahtera J, Keltikangas-Jarvinen L. Workplace bullying and the risk of cardiovascular disease and depression. Occup Environ Med. 2003;60(10):779-83.
123. Kivimaki M, Honkonen T, Wahlbeck K, Elovainio M, Pentti J, Klaukka T et al. Organisational downsizing and increased use of psychotropic drugs among employees who remain in employment. Journal of Epidemiology and Community Health. 2007;61(2):154-8. doi:10.1136/jech.2006.050955.
124. Kleppa E, Sanne B, Tell GS. Working overtime is associated with anxiety and depression: the Hordaland Health Study. J Occup Environ Med. 2008;50(6):658-66. doi:10.1097/JOM.0b013e3181734330.
125. Laaksonen M, Lallukka T, Lahelma E, Partonen T. Working conditions and psychotropic medication: A prospective cohort study. Social Psychiatry and Psychiatric Epidemiology: The International Journal for Research in Social and Genetic Epidemiology and Mental Health Services. 2012;47(4):663-70. doi:10.1007/s00127-011-0372-x.
126. Madsen IEH, Nyberg ST, Magnusson Hanson LL, Ferrie JE, Ahola K, Alfredsson L et al. Job strain as a risk factor for clinical depression: systematic review and meta-analysis with additional individual participant data. Psychol Med. 2017;47(8):1342-56. doi:10.1017/s003329171600355x.
127. Niedhammer I, Malard L, Chastang JF. Occupational factors and subsequent major depressive and generalized anxiety disorders in the prospective French national SIP study. BMC Public Health. 2015;15:200. doi:10.1186/s12889-015-1559-y.
128. Nielsen MB, Madsen IE, Aust B, Burr H, Rugulies R. Effort-reward imbalance at work and the risk of antidepressant treatment in the Danish workforce. J Affect Disord. 2016;196:248-51. doi:10.1016/j.jad.2016.02.038.
129. Oyane NM, Pallesen S, Moen BE, Akerstedt T, Bjorvatn B. Associations between night work and anxiety, depression, insomnia, sleepiness and fatigue in a sample of Norwegian nurses. PLoS ONE. 2013;8(8):e70228. doi:10.1371/journal.pone.0070228.
130. Rugulies R, Bultmann U, Aust B, Burr H. Psychosocial work environment and incidence of severe depressive symptoms: prospective findings from a 5-year follow-up of the Danish work environment cohort study. Am J Epidemiol. 2006;163(10):877-87. doi:10.1093/aje/kwj119.
131. Rugulies R, Thieien K, Nygaard E, Diderichsen F. Job insecurity and the use of antidepressant medication among Danish employees with and without a history of prolonged unemployment: A 3.5-year follow-up study. Journal of Epidemiology and Community Health. 2010;64(1):75-81. doi:10.1136/jech.2008.078493.
132. Rugulies R, Madsen IEH, Hjarsbech PU, Hogh A, Borg V, Carneiro IG et al. Bullying at work and onset of a major depressive episode among Danish female eldercare workers. Scandinavian Journal of Work Environment & Health. 2012;38(3):218-27. doi:10.5271/sjweh.3278.
133. Thielen K, Nygaard E, Rugulies R, Diderichsen F. Job stress and the use of antidepressant medicine: a 3.5-year follow-up study among Danish employees. Occup Environ Med. 2011;68(3):205-10. doi:10.1136/oem.2010.057943.

134. Tokuyama M, Nakao K, Seto M, Watanabe A, Takeda M. Predictors of first-onset major depressive episodes among white-collar workers. Psychiatry Clin Neurosci. 2003;57(5):523-31. doi:10.1046/j.1440-1819.2003.01158.x.
135. Vammen MA, Mikkelsen S, Hansen AM, Bonde JP, Grynderup MB, Kolstad H et al. Emotional Demands at Work and the Risk of Clinical Depression: A Longitudinal Study in the Danish Public Sector. J Occup Environ Med. 2016;58(10):994-1001. doi:10.1097/jom.0000000000000849.
136. Varma A, Marott JL, Stoltenberg CDG, Wieclaw J, Kolstad HA, Bonde JP. With long hours of work, might depression then lurk? A nationwide prospective follow-up study among Danish senior medical consultants. Scandinavian Journal of Work Environment & Health. 2012;38(5):418-26. doi:10.5271/sjweh.3268.
137. Virtanen M, Honkonen T, Kivimaki M, Ahola K, Vahtera J, Aromaa A et al. Work stress, mental health and antidepressant medication findings from the Health 2000 Study. J Affect Disord. 2007;98(3):189-97. doi:10.1016/j.jad.2006.05.034.
138. Virtanen M, Pentti J, Vahtera J, Ferrie JE, Stansfeld SA, Helenius H et al. Overcrowding in hospital wards as a predictor of antidepressant treatment among hospital staff. The American Journal of Psychiatry. 2008;165(11):1482-6. doi:10.1176/appi.ajp.2008.07121929.
139. Wege N, Angerer P, Li J. Effects of Lifetime Unemployment Experience and Job Insecurity on Two-Year Risk of Physician-Diagnosed Incident Depression in the German Working Population. Int J Environ Res Public Health. 2017;14(8). doi:10.3390/ijerph14080904.
140. Wirth MD, Shivappa N, Burch JB, Hurley TG, Hebert JR. The Dietary Inflammatory Index, shift work, and depression: Results from NHANES. Health Psychol. 2017;36(8):760-9. doi:10.1037/hea0000514.
141. Ylipaavalniemi J, Kivimaki M, Elovainio M, Virtanen M, Keltikangas-Jarvinen L, Vahtera J. Psychosocial work characteristics and incidence of newly diagnosed depression: a prospective cohort study of three different models. Soc Sci Med. 2005;61(1):111-22. doi:10.1016/j.socscimed.2004.11.038.
142. Wing JK, Sartorius N, Üstun TB. WHO diagnosis and clinical measurement in psychiatry. A reference manual for SCAN. Cambridge: Cambridge University Press; 1998.
143. Robins LN, Wing J, Wittchen HU, Helzer JE, Babor TF, Burke J et al. The Composite International Diagnostic Interview. An epidemiologic Instrument suitable for use in conjunction with different diagnostic systems and in different cultures. Arch Gen Psychiatry. 1988;45(12):1069-77.
144. Kessler RC, Wittchen HU, Abelson J, McGonagle KA, Schwarz N, Kendler KS et al. Methodological studies of the Composite International Diagnostic Interview (CIDI) in the US National Comorbidity Survey (NCS). International Journal of Methods in Psychiatric Research. 1998;7(1):33-55.
145. Kessler RC, Andrews G, Mroczek D, Ûstün B, Wittchen HU. The World Health Organization Composite International Diagnostic Interview Short-Form (CIDI-SF). International Journal of Methods in Psychiatric Research. 1998;7(4):171-84.
146. Kessler RC, Ustun TB. The World Mental Health (WMH) Survey Initiative Version of the World Health Organization (WHO) Composite International Diagnostic Interview (CIDI). Int J Methods Psychiatr Res. 2004;13(2):93-121.
147. Sheehan DV, Lecrubier Y, Sheehan KH, Amorim P, Janavs J, Weiller E et al. The Mini-International Neuropsychiatric Interview (M.I.N.I.): the development and validation of a structured diagnostic psychiatric interview for DSM-IV and ICD-10. J Clin Psychiatry. 1998;59 Suppl 20:22-33.
148. Radloff L. The CES-D Scale: A self-report depression scale for research in the general population. Applied Psychological Measurement. 1977;1(3):385-401.
149. Zigmond AS, Snaith RP. The Hospital Anxiety and Depression Scale. Acta psychiatrica Scandinavica. 1983;67:361-70.
150. Bech P, Olsen LR, Kjoller M, Rasmussen NK. Measuring well-being rather than the absence of distress symptoms: a comparison of the SF-36 Mental Health subscale and the WHO-Five Well-Being Scale. Int J Methods Psychiatr Res. 2003;12(2):85-91.
151. Berwick DM, Murphy JM, Goldman PA, Ware JE, A.J. B, Weinstein MC. Performance of a five-item mental health screening test. Medical Care. 1991;29:169-76.

152. Kroenke K, Spitzer RL, Williams JB. The PHQ-9: validity of a brief depression severity measure. Journal of general internal medicine. 2001;16(9):606-13.
153. Zung WW, Magruder-Habib K, Velez R, Alling W. The comorbidity of anxiety and depression in general medical patients: a longitudinal study. J Clin Psychiatry. 1990;51 Suppl:77-80; discussion 1.
154. Spitzer RL. Psychiatric diagnosis: are clinicians still necessary? Comprehensive psychiatry. 1983;24(5):399-411.
155. Kurdyak PA, Gnam WH. Small signal, big noise: performance of the CIDI depression module. Canadian journal of psychiatry Revue canadienne de psychiatrie. 2005;50(13):851-6. doi:10.1177/070674370505001308.
156. Klinkman MS, Coyne JC, Gallo S, Schwenk TL. False positives, false negatives, and the validity of the diagnosis of major depression in primary care. Arch Fam Med. 1998;7(5):451-61. doi:10.1001/archfami.7.5.451 [doi].
157. Christensen KS, Toft T, Frostholm L, Ornbol E, Fink P, Olesen F. The FIP study: a randomised, controlled trial of screening and recognition of psychiatric disorders. The British journal of general practice : the journal of the Royal College of General Practitioners. 2003;53(495):758-63.
158. Aragones E, Pinol JL, Labad A. The overdiagnosis of depression in non-depressed patients in primary care. Family practice. 2006;23(3):363-8. doi:cmi120 [pii]
10.1093/fampra/cmi120 [doi].
159. Fernandez A, Pinto-Meza A, Bellon JA, Roura-Poch P, Haro JM, Autonell J et al. Is major depression adequately diagnosed and treated by general practitioners? Results from an epidemiological study. Gen Hosp Psychiatry. 2010;32(2):201-9. doi:S0163-8343(09)00243-6 [pii];10.1016/j.genhosppsych.2009.11.015 [doi].
160. Gerber PD, Barrett J, Barrett J, Manheimer E, Whiting R, Smith R. Recognition of depression by internists in primary care: a comparison of internist and "gold standard" psychiatric assessments. Journal of general internal medicine. 1989;4(1):7-13. doi:10.1007/bf02596483 [doi].
161. Sanchez-Villegas A, Schlatter J, Ortuno F, Lahortiga F, Pla J, Benito S et al. Validity of a self-reported diagnosis of depression among participants in a cohort study using the Structured Clinical Interview for DSM-IV (SCID-I). BMC Psychiatry. 2008;8:43. doi:1471-244X-8-43 [pii];10.1186/1471-244X-8-43 [doi].
162. Andrews G, Anstey K, Brodaty H, Issakidis C, Luscombe G. Recall of depressive episode 25 years previously. Psychol Med. 1999;29(4):787-91.
163. Alonso J, Angermeyer MC, Bernert S, Bruffaerts R, Brugha TS, Bryson H et al. Use of mental health services in Europe: results from the European Study of the Epidemiology of Mental Disorders (ESEMeD) project. Acta psychiatrica Scandinavica Supplementum. 2004(420):47-54. doi:10.1111/j.1600-0047.2004.00330.x [doi] ACP330 [pii].
164. Mack S, Jacobi F, Gerschler A, Strehle J, Hofler M, Busch MA et al. Self-reported utilization of mental health services in the adult German population--evidence for unmet needs? Results of the DEGS1-Mental Health Module (DEGS1-MH). International journal of methods in psychiatric research. 2014;23(3):289-303. doi:10.1002/mpr.1438 [doi].
165. Gardarsdottir H, Heerdink ER, van DL, Egberts AC. Indications for antidepressant drug prescribing in general practice in the Netherlands. J Affect Disord. 2007;98(1-2):109-15. doi:S0165-0327(06)00313-2 [pii];10.1016/j.jad.2006.07.003 [doi].
166. Demyttenaere K, Bruffaerts R, Posada-Villa J, Gasquet I, Kovess V, Lepine JP et al. Prevalence, severity, and unmet need for treatment of mental disorders in the World Health Organization World Mental Health Surveys. JAMA. 2004;291(21):2581-90. doi:10.1001/jama.291.21.2581 [doi];291/21/2581 [pii].
167. Ramirez Basco M, Bostic JQ, Davies D, Rush AJ, Witte B, Hendrickse W et al. Methods to improve diagnostic accuracy in a community mental health setting. Am J Psychiatry. 2000;157(10):1599-605. doi:10.1176/appi.ajp.157.10.1599.
168. Miller PR, Dasher R, Collins R, Griffiths P, Brown F. Inpatient diagnostic assessments: 1. Accuracy of structured vs. unstructured interviews. Psychiatry Res. 2001;105(3):255-64.

169. Taiminen T, Ranta K, Karlsson H, Lauerma H, Leinonen KM, Wallenius E et al. Comparison of clinical and best-estimate research DSM-IV diagnoses in a Finnish sample of first-admission psychosis and severe affective disorder. Nord J Psychiatry. 2001;55(2):107-11. doi:10.1080/08039480151108507 [doi].
170. Andreas S, Theisen P, Mestel R, Koch U, Schulz H. Validity of routine clinical DSM-IV diagnoses (Axis I/II) in inpatients with mental disorders. Psychiatry Res. 2009;170(2-3):252-5. doi:S0165-1781(08)00348-X [pii];10.1016/j.psychres.2008.09.009 [doi].
171. Davis KA, Sudlow CL, Hotopf M. Can mental health diagnoses in administrative data be used for research? A systematic review of the accuracy of routinely collected diagnoses. BMC psychiatry. 2016;16:263. doi:10.1186/s12888-016-0963-x.
172. Bock C, Bukh JD, Vinberg M, Gether U, Kessing LV. Validity of the diagnosis of a single depressive episode in a case register. Clinical practice and epidemiology in mental health : CP & EMH. 2009;5:4. doi:10.1186/1745-0179-5-4.
173. Theorell T, Hammarstrom A, Aronsson G, Traskman Bendz L, Grape T, Hogstedt C et al. A systematic review including meta-analysis of work environment and depressive symptoms. BMC Public Health. 2015;15:738. doi:10.1186/s12889-015-1954-4.
174. Brugha TS, Jenkins R, Taub N, Meltzer H, Bebbington PE. A general population comparison of the Composite International Diagnostic Interview (CIDI) and the Schedules for Clinical Assessment in Neuropsychiatry (SCAN). Psychol Med. 2001;31(6):1001-13.
175. Kessler RC, Berglund P, Demler O, Jin R, Koretz D, Merikangas KR et al. The epidemiology of major depressive disorder: results from the National Comorbidity Survey Replication (NCS-R). JAMA. 2003;289(23):3095-105. doi:10.1001/jama.289.23.3095 [doi];289/23/3095 [pii].
176. Aalto-Setala T, Haarasilta L, Marttunen M, Tuulio-Henriksson A, Poikolainen K, Aro H et al. Major depressive episode among young adults: CIDI-SF versus SCAN consensus diagnoses. Psychol Med. 2002;32(7):1309-14.
177. de Azevedo Marques JM, Zuardi AW. Validity and applicability of the Mini International Neuropsychiatric Interview administered by family medicine residents in primary health care in Brazil. General hospital psychiatry. 2008;30(4):303-10. doi:S0163-8343(08)00030-3 [pii] 10.1016/j.genhosppsych.2008.02.001 [doi].
178. Jahn R, Baumgartner JS, van den Nest M, Friedrich F, Alexandrowicz RW, Wancata J. [Criterion Validity of the German Version of the CES-D in the General Population]. Psychiatrische Praxis. 2018;45(8):434-42. doi:10.1055/a-0584-9803.
179. Vilagut G, Forero CG, Barbaglia G, Alonso J. Screening for Depression in the General Population with the Center for Epidemiologic Studies Depression (CES-D): A Systematic Review with Meta-Analysis. PLoS One. 2016;11(5):e0155431. doi:10.1371/journal.pone.0155431.
180. Abiodun O. A validity study of the Hospital Anxiety and Depression Scale in general hospital units and a community sample in Nigeria. British Journal of Psychiatry. 1994;165(5):669-72.
181. Kjærgaard M, Wang CEA, Waterloo K, Jorde R. A study of the psychometric properties of the Beck Depression Inventory-II, the Montgomery and Åsberg Depression Rating Scale, and the Hospital Anxiety and Depression Scale in a sample from a healthy
population. Scandinavian journal of psychology. 2014;55:83-9.
182. Forsell Y. The Major Depression Inventory versus Schedules for Clinical Assessment in Neuropsychiatry in a population sample. Soc Psychiatry Psychiatr Epidemiol. 2005;40(3):209-13. doi:10.1007/s00127-005-0876-3 [doi].
183. Rumpf HJ, Meyer C, Hapke U, John U. Screening for mental health: validity of the MHI-5 using DSM-IV Axis I psychiatric disorders as gold standard. Psychiatry Res. 2001;105(3):243-53.
184. Patten SB, Burton JM, Fiest KM, Wiebe S, Bulloch AG, Koch M et al. Validity of four screening scales for major depression in MS. Multiple sclerosis (Houndmills, Basingstoke, England). 2015;21(8):1064-71. doi:10.1177/1352458514559297.

185. Lowe B, Spitzer RL, Grafe K, Kroenke K, Quenter A, Zipfel S et al. Comparative validity of three screening questionnaires for DSM-IV depressive disorders and physicians' diagnoses. J Affect Disord. 2004;78(2):131-40. doi:S0165032702002379 [pii].
186. Campo-Arias A, Diaz-Martinez L, Eduardo G, Jaimes R, Cadena L, Hernandez N. Validation of Zung's self-rating depression scale among the Colombian general population. Social Behavior and Personality. 2006;34(1):87-94.
